# Automated image analysis of stained cytospins to quantify Schwann cell purity and proliferation

**DOI:** 10.1371/journal.pone.0233647

**Published:** 2020-05-22

**Authors:** Tamara Weiss, Lorenz Semmler, Flavia Millesi, Anda Mann, Maximilian Haertinger, Manuel Salzmann, Christine Radtke

**Affiliations:** 1 Research Laboratory of the Division of Plastic and Reconstructive Surgery, Department of Surgery, Medical University of Vienna, Vienna, Austria; 2 Institute of Vascular Biology and Thrombosis Research, Medical University of Vienna, Vienna, Austria; 3 Division of Plastic and Reconstructive Surgery, Department of Surgery, Medical University of Vienna, Vienna, Austria; Universidade Federal do Rio de Janeiro, BRAZIL

## Abstract

In response to injury, adult Schwann cells (SCs) re-enter the cell cycle, change their expression profile, and exert repair functions important for wound healing and the re-growth of axons. While this phenotypical instability of SCs is essential for nerve regeneration, it has also been implicated in cancer progression and de-myelinating neuropathies. Thus, SCs became an important research tool to study the molecular mechanisms involved in repair and disease and to identify targets for therapeutic intervention. A high purity of isolated SC cultures used for experimentation must be demonstrated to exclude that novel findings are derived from a contaminating fibroblasts population. In addition, information about the SC proliferation status is an important parameter to be determined in response to different treatments. The evaluation of SC purity and proliferation, however, usually depends on the time consuming, manual assessment of immunofluorescence stainings or comes with the sacrifice of a large amount of SCs for flow cytometry analysis. We here show that rat SC culture derived cytospins stained for SC marker SOX10, proliferation marker EdU, intermediate filament vimentin and DAPI allowed the determination of SC identity and proliferation by requiring only a small number of cells. Furthermore, the CellProfiler software was used to develop an automated image analysis pipeline that quantified SCs and proliferating SCs from the obtained immunofluorescence images. By comparing the results of total cell count, SC purity and SC proliferation rate between manual counting and the CellProfiler output, we demonstrated applicability and reliability of the established pipeline. In conclusion, we here combined the cytospin technique, a multi-colour immunofluorescence staining panel, and an automated image analysis pipeline to enable the quantification of SC purity and SC proliferation from small cell aliquots. This procedure represents a solid read-out to simplify and standardize the quantification of primary SC culture purity and proliferation.

## Introduction

Schwann cells (SCs) are the principal glia of the peripheral nervous system. Myelinating and Remark (non-myelinating) SCs ensure the structural and functional integrity of axons, whereas terminal SCs regulate synaptic homeostasis at the neuromuscular junctions. Despite being important for the correct development and preservation of peripheral nerve fibers, SCs increasingly gain recognition as a highly plastic cell type involved in various pathophysiological processes. SC plasticity refers to their reactive nature, which allows adult SCs to respond adaptively to peripheral nerve damage [1]. Thereby, SCs undergo trans-differentiation into a dedicated repair cell, which is essential to promote regeneration of the injured nerve [2, 3]. However, extended loss of axon contact decreases the support of repair SCs, which is considered a main reason for regeneration failure [4–7]. Understanding how the repair SC state can be sustained or how its repair functions can be exploited therapeutically is crucial to enhance regenerative approaches for nerve repair. Moreover, SC plasticity is also implicated in cancer progression and peripheral neuropathies. The interaction of cancer cells with SCs was shown to induce repair SC characteristics and promote perineural invasion; the ability of cancer cells to invade and grow along nerves [8, 9]. Furthermore, the upregulation of repair SC associated receptors could play a role in neuropathic pain and inflammatory neuropathies [10–13]. Thus, investigating the molecular mechanisms of SC plasticity in injury and disease is important to reveal therapeutic targets for the development of novel treatment strategies.

Refined isolation and culture techniques have been established to study the functions, interactions, and molecular processes of repair SCs *in vitro* [14–19]. Importantly, peripheral nerve derived SC cultures reflect the repair SC phenotype emerging in injured nerves as they express repair SC associated molecules and execute repair specific tasks such as myelin clearance [3, 20]. In addition, tumour-associated SCs or SCs from patients with neuropathies can be isolated from biopsies [21, 22]. However, primary SC cultures also include a fibroblast (FB) population derived from the surrounding connective tissue. Several methods are available to enrich SCs from FBs [14, 17, 23–28], which contaminate the SC cultures and are likely to distort the results obtained from sensitive analysis technologies such as transcriptomics and proteomics. Hence, information about the achieved SC purity has to be routinely demonstrated for each SC culture used for experimentation. Furthermore, the proliferation status is an important parameter to evaluate the effect of different treatments on SCs *in vitro*. The purity and proliferation rate of SC cultures is most commonly determined by flow cytometry or immunocytochemistry, but both methods come with certain disadvantages. Analysis of SCs via flow cytometry demands a high number of valuable cells while immunofluorescence staining results of sister cultures not necessarily reflect the main culture properties. Additionally, manual evaluation of stained (proliferating) SCs is a time-consuming procedure.

To meet the need for a fast and reliable quantification of SC culture purity and proliferation from a small number of cells, we here combined the cytospin technique, a multi-colour immunofluorescence staining panel, and automated image analysis. Cytospins refer to the concentration of cells on a microscope slide via centrifugation and can be prepared from small cell aliquots. The multi-colour immunofluorescence panel enables to provide information about SC identity and their proliferation status at once, which further reduced the numbers of cells to be analysed. The open-source software CellProfiler is well established to measure and quantify cellular features [29, 30] and was used to develop an automated image analysis pipeline that retrieves information about SC culture purity and proliferation based on stained cytospins.

## Methods

### Animals

Female Sprague-Dawley rats were housed with nesting material and environmental enrichment in group cages in a temperature‐controlled environment, exposed to a 12 hour light–dark cycle and supplied with food and water ad libitum. The animals were sacrificed using a rising CO_2_ atmosphere according to the guidelines of Austrian legislator.

### Primary rat Schwann cell culture

Rat SC cultures were established from adult 12–14 weeks old female Sprague-Dawley rat derived sciatic nerves. Therefore, we adapted a published protocol for the culture of human SCs [17]. Briefly, the excised nerve tissue was washed in fresh 1X Dulbecco’s Phosphate Buffered Saline (PBS, GIBCO) + 1% antibiotic-antimycotic (GIBCO) and transferred to an autoclaved glass dish filled with supplemented MEM∝ consisting of MEM∝ GlutaMAX^TM^-I (GIBCO) enriched with 2.5% HEPES (GIBCO), 1% Penicillin-Streptomycin (P/S) (GIBCO), 10% fetal calf serum (FCS, LINARIS) and 1% Sodium Pyruvate (GIBCO). The fascicles were pulled out of the epineurium and cut into 1–3 mm long pieces. The fascicle pieces were transferred into a well of a 6-well plate (Cellstar) and incubated with 2 ml digestion solution consisting of supplemented MEM∝ + 0.125% (w/v) collagenase type IV (GIBCO), 1.25 U/ml Dispase II (SIGMA) and 3 mM Calcium chloride (Merck) overnight (15-20h) at 37°C and 5% CO_2_.

On the next day, the enzymatic digest was stopped and the pellet was resuspended in 2 ml SC expansion medium (SCEM) consisting of MEM∝ enriched with 1% P/S, 1% Sodium Pyruvate, 2.5% HEPES, 5% FCS, 10 ng/ml recombinant Heregulinβ-1 (PeproTech), 0.5% N-2 Supplement (GIBCO), 2 μM forskolin (SIGMA), 10 ng/ml recombinant FGF basic (PeproTech) and 5 ng/ml PDGFAA (PeproTech). The cell suspension was seeded on a 0.01% poly-L-lysine hydrobromide (PLL, SIGMA) and 5 μg/ml laminin (SIGMA) coated well of a 6-well plate. Half of the medium was changed three times a week. Cell cultures derived from peripheral nerves consist of SCs and FBs. SCs were purified from FBs according to the two-step enrichment procedure that is based on the different adhesion properties of SCs and FBs [17, 20]. After enrichment, SCs were re-seeded and expanded in wells of a 6-well plate (1.25x10^5^ cells/well).

### Cytospin preparation

Cytocentrifugation refers to the concentration of cells on a microscope slide via centrifugation. Cell aliquots derived from the main SC culture were used for cytospin preparations established with the SHANDON Cytospin 3 cytocentrifuge according to the manufacturer’s protocol and as described previously [17]. Briefly, the cytofunnels, filter cards and microscope slides were assembled and pre-wet with 50 μl NaCl solution by centrifugation at 600 rpm for 1 min. 10,000 SCs in a volume of 50 μl supplemented MEM∝ were added to each cytofunnel and centrifuged at 450 rpm for 7 min. The microscope slide with cytospun cells was carefully detached from the cytofunnel and filter card and left to dry for 1 h at room temperature (RT). The cytospins were immediately processed or stored in a microscope slide box at -20°C until immunofluorescence analysis.

### Immunocytochemistry

The procedure is carried out at RT. The washing involves a sequential incubation with 1XPBS for 5 min each. All antibody details are listed in **Table 1**. For immunofluorescence staining analysis of grown SC cultures, 1x10^4^ SCs were seeded per well of a PLL/laminin coated 8-well chamber slide (ibidi) and cultured in SCEM until a confluency of about 70–80% was reached. SC cultures were washed before fixation. Grown SC cultures and cytospins were fixed with 4.5% formaldehyde solution (SAV Liquid Production GmbH) for 15 minutes. Cells were washed again followed by blocking and permeabilization with 1XPBS + 1% bovine serum albumin (BSA, SIGMA), + 0.3% Triton-X (SIGMA) + 5% goat serum (DAKO) for 10 minutes. If required, the EdU staining was performed after permeabilization according to the manufacturer’s protocol (Click-iT® Plus EdU Imaging Kit). Subsequently, the cells were incubated with respective primary antibodies in 1XPBS + 1% BSA + 0.1% TritonX-100 + 1% goat serum for 2 hours. After washing, the cells were incubated with the antibody mix containing respective secondary antibodies in 1XPBS + 1% BSA + 1% goat serum for one hour. Then, cells were washed and incubated with 50 μg/ml 4',6-Diamidino-2-Phenylindole (DAPI) solution (ThermoScientific) for 10 minutes and washed again. Grown SC cultures were embedded in FluoromountG mounting medium (Invitrogen). Cytospun cells were embedded in Vectashield mounting medium (Vector Laboratories, Inc.), carefully covered with a coverslip and sealed with glue. Immunofluorescence images of grown cells were taken using a confocal laser scanning microscope (LEICA SP8X). Images are depicted as maximum projection of total z-stacks. Immunofluorescence images of cytospins, covering around 800 cells per donor, were taken with the NIKON Eclipse Ti microscope. Brightness and contrast of images were adjusted in a homogenous manner. For CellProfiler analysis, raw images of single DAPI, VIME, and SOX10 channels were used.

**Table 1:** Antibodies.

**1**^**st**^ **Antibodies**			
**Reactivity**	**Species**	**company**	**dilution**
S100	rabbit	DAKO	1:200
SOX10	mouse	Santa Cruz	1:50
vimentin	chicken	Invitrogen	1:200
**2**^**nd**^ **Antibodies**			
**Reactivity**	Fluorophore	company	dilution
rabbit	AF488P	Invitrogen	1:600
mouse	AF594	Invitrogen	1:300
mouse	AF488	Invitrogen	1:300
chicken	DL650	Invitrogen	1:300

### Manual counting of cells

Manual counting of cytospin images including the staining for SOX10/VIME/EdU/DAPI of 3 donors was performed by three independent researchers using the CellCounter plug-in of the FIJI software. At least 600 DAPI^+^ nuclei were counted per donor by excluding burst nuclei and nuclei cut by the image boarder. Cells without a defined VIME^+^ cell body were also excluded, which resulted in the total number of *real cells*. From the real cells, SOX10^+^ SCs and SOX10^+^/EdU^+^ proliferating SCs were counted.

### Automated image analysis using CellProfiler

The CellProfiler software provides pipelines with configurable image processing modules that are run consecutively for automated image analysis [29]. Here, the CellProfiler (3.0.0) [31] was used to extract information about SC identity and the proliferation status from cytospin images that included stainings for SOX10, EdU, VIME and DAPI. The settings and function of applied image processing modules are summarized in **Table 2**, the pipeline is provided for download (**S1 File**). In the following, the main analysis steps are described:

**Table 2:** CellProfiler pipeline with used modules and settings.

	Function	Module	Setting
**Identification of cell nuclei**	*Identifies DAPI*^*+*^ *cell nuclei based on the DAPI stain image*	1) IdentifyPrimaryObjects	Object diameter in pixel units: 16–60
			Global threshold strategy
			two-classes Otsu threshold method
			smoothening scale: 1.3488
			correction factor: 0.7
			lower and upper bound threshold: 0.0 and 1.0
			distinguish clumped objects: shape
			dividing line between clumped objects: propagate
	*Measures shape parameters of identified nuclei*	2) MeasureObjectSizeShape	Use defaults
	*Excludes burst nuclei by filtering identified nuclei according to shape parameters*	3) FilterObjects	AreaShape measurement by FormFactor with a minimum value of 0.599
**Identification of SC nuclei**	*Measures SOX10 intensity of nuclei in SOX10 stain image*	4) MeasureObjectIntensity	Use defaults
	*Displays mean SOX10 intensity on SOX10 stain image to aid setting of correct threshold*	5) DisplayDataOnImage	Use defaults
	*Filters identified nuclei for SOX10 positivity using the manually defined threshold for SOX10 positivity to identify SCs (= SOX10*^*+*^ *nuclei)*	6) FilterObjects	filtering mode: Measurement
			Filtering method: Limit
			MeanIntensity with a minimum value of the defined threshold
**Identification of proliferating SC nuclei**	*Measures EdU intensity of nuclei in the EdU stain image*	7) MeasureObjectIntensity	Use defaults
	*Displays mean EdU intensity on EdU stain image to aid setting of correct threshold*	8) DisplayDataOnImage	Use defaults
	*Filters nuclei for EdU positivity according to the manually defined threshold for EdU positivity to identify proliferating SCs (= SOX10*^*+*^*/EdU*^*+*^ *nuclei)*	9) FilterObjects	Filtering mode: Measurement
			Filtering method: Limit
			MeanIntensity with a minimum value of the defined threshold
**Identification of intact cells**	*Identifies all cell bodies associated with nuclei based on VIME stain image*	10) IdentifySecondaryObjects	Propagation from identified real nuclei
			Global threshold strategy
			two-classes Otsu threshold method
			smoothening scale: 1.3488
			correction factor: 0.9
			lower and upper bound threshold: 0.0 and 1.0
			regularization factor: 0.1
	*Measures shape parameters of identified cell bodies*	11) MeasureObjectSizeShape	Use defaults
	*Measures VIME intensity of identified cell bodies*	12) MeasureObjectIntensity	Use defaults
	*Filters identified cell bodies according to shape and intensity parameters to exclude identified nuclei*, *SOX10*^*+*^ *nuclei and SOX10*^*+*^*/EdU*^*+*^ *nuclei without an intact cell body*	13) FilterObjects	filtering mode: Measurement
			Filtering method: Limit
			AreaShape Measurement by Area
			Minimum *Area* measurement: 28
			Intensity measurement by MeanIntensity based on VIME stain with a minimal value of 0.000009,
			AreaShape measurement by FormFactor with a minimum value of 0.35
**Visualization of data**	*Displays cell count of identified VIME*^*+*^ *cells on VIME stain image*	14) DisplayDataOnImage	Use defaults
	*Displays cell count of identified SOX10*^*+*^ *cells on SOX10 stain image*	15) DisplayDataOnImage	Use defaults
	*Displays cell count of identified SOX10*^*+*^*/EdU*^*+*^ *cells on EdU stain image*	16) DisplayDataOnImage	Use defaults
	*Displays SOX10*^*+*^ *nuclei in red and SOX10*^*-*^ *nuclei in green on SOX10 stain image*	17) ClassifyObjects	Use defaults
	*Displays SOX10*^*+*^*/EdU*^*+*^ *in red and SOX10*^*+*^*/EdU*^*-*^ *nuclei in green on EdU stain image*	18) ClassifyObjects	Use defaults
**Data export**	*Saves mean SOX10 staining intensity of nuclei on SOX10 stain image*	19) SaveImages	Use defaults
	*Saves mean EdU staining intensity of SOX10*^*+*^ *nuclei on EdU stain image*	20) SaveImages	Use defaults
	*Saves classified SOX10*^*-*^ *nuclei (green) and SOX10*^*+*^ *nuclei (red) on SOX10 stain image*	21) SaveImages	Use defaults
	*Saves classified SOX10*^*+*^*/ EdU*^*-*^ *nuclei (green) and SOX10*^*+*^*/EdU*^*+*^ *nuclei (red) on EdU stain image*	22) SaveImages	Use defaults
	*Saves VIME*^*+*^ *cell count (total cells) on VIME stain image*	23) SaveImages	Use defaults
	*Saves SOX10*^*+*^ *cell count (SCs) on SOX10 stain image*	24) SaveImages	Use defaults
	*Saves SOX10*^*+*^*/EdU*^*+*^ *cell count (proliferating SCs) on EdU stain image*	25) SaveImages	Use defaults
	*Exports counts of VIME*^*+*^ *cells (total cells)*, *SOX10*^*+*^ *cells (SCs) and SOX10*^*+*^*/EdU*^*+*^ *cells (proliferating SCs) to spreadsheet format*	26) ExportToSpreadsheet	Use defaults

### Identification of intact cell nuclei

The CellProfiler algorithm uses a modular strategy to identify objects based on previously published algorithms [32–36]. In a first step, the *IdentifyPrimaryObjects* module was used to identify intact cell nuclei of DAPI input images. Objects outside the defined diameter of 16–60 pixel units were excluded. A global threshold strategy using a two-class Otsu’s method [37] was chosen to automatically calculate a single threshold value based on the unmasked pixels of the DAPI input image. This method is suitable for images with a uniform background. Pixels above the threshold value are classified as DAPI^+^ foreground, pixels below are classified as DAPI^-^ background. If necessary, the input images can be smoothed prior to thresholding to remove unwanted artefacts by adapting the smoothing scale according to the dimensions of the unwanted artefacts. Adjusting the threshold correction factor enables to correct the threshold upwards (more stringent) or downwards (more lenient) by multiplying it by the set value. As the cytospin images might show some variations, e.g. in staining intensities, a threshold correction factor of 0.7 was applied.

The nuclei of cytospun cells are usually rounded and show a definite indentation when clumped together that favours a “shape” based object segmentation. This converts the binary threshold image to a distance-transformed version that determines the object centers as maxima. Each pixel is given a value equal to the nearest pixel below the threshold, indicating the object shape. The dividing line between segmented objects is drawn according to a propagation algorithm that assigns pixels to the objects by repeatedly adding unassigned pixels to immediately adjacent object [38]. To optimize segmentation, a minimal allowed distance between local maxima is automatically applied. Based on the minimum object diameter, local maxima that are closer together than the minimum object diameter are suppressed. Based on the defined object diameter set at the beginning, a smoothing filter is automatically calculated. In case of under- or over-segmentation of objects, the smoothing filter value can be decreased or increased, respectively, to assist proper segmentation. Eventual holes within the identified objects were filled. Objects cut by the image border were excluded as only whole nuclei should be used for downstream measurements. To verify whether the applied settings successfully identified cell nuclei, preliminary segmenting results can be visualized using the *DisplayDataOnImage* module.

After identification of DAPI^+^ cell nuclei, burst nuclei can be excluded from further analysis. First, the circularity (FormFactor) of each object was calculated (4*π*Area/Perimeter^2^) in the *MeasureObjectSizeShape* module [29, 39, 40]. Note that a result of 1 equals a perfectly circular object. As burst nuclei have a reduced circularity when compared to intact cell nuclei, we then filtered for objects with a minimum FormFactor of 0.599 in the *FilterObject* module that resulted in the successful segmentation of real nuclei.

### Identification of SC nuclei

In order to determine whether the identified real nuclei belong to a SC or not, the SOX10 stain mean intensity (average pixel intensity) of each identified real nucleus was calculated using the *MeasureObjectIntensity* module. CellProfiler by default rescales the intensity from 0 to 1 by dividing all pixels by the maximum possible intensity value. As staining intensities can be influenced by the staining procedure or donor variations, the threshold for SOX10 positivity should be manually adapted by the operator per analysed cytospin. Assessment of the threshold was facilitated by displaying the mean SOX10 intensity per identified real nucleus (primary object) using the *DisplayDataOnImage* module. According to the defined threshold, *real nuclei* can be filtered for SOX10 positivity in the *FilterObject* module to identify SOX10^+^ real nuclei.

### Identification of proliferating SC nuclei

Afterwards, the identified SC nuclei (*SOX10*^*+*^ *real nuclei*) were categorized as proliferating and non-proliferating. As for SOX10, the EdU stain mean intensity of each identified real nucleus was calculated using the *MeasureObjectIntensity* module. Displaying the calculated mean EdU intensity per identified real nucleus (*DisplayDataOnImage* module) enabled to define the threshold for EdU positivity and to filter (*FilterObject* module) for SOX10^+^/EdU^+^ real nuclei.

### Identification of intact cells

To include only intact cells, defined as cells having both a nucleus and a cell body, in the analysis, VIME^+^ cell bodies were identified in the *IdentifySecondaryObjects* module. Similar to the *IdentifyPrimaryObjects* module, a global threshold strategy using a two-class Otsu’s method [37] was applied. The threshold correction factor was set to 0.9. Based on the VIME input image, the applied propagation algorithm uses primary objects as ‘seed’ for propagation and, thus, assigns pixels to the nearest real nucleus [38]. Borders are determined by combining information about the distance to the nearest primary object and VIME intensity gradients. The distances are calculated as the sum of absolute differences in a 3x3 (8-connected) image neighbourhood. Borders are set where the images local appearance changes perpendicularly to the border. By adjusting the regularization factor λ, the segmentation of objects can be balanced between by the distance to the nearest primary objects and the intensity of the secondary object. As cytospin samples usually result in a homogenous distribution of cells, we chose a λ value of 0.1, which favoured an increased distance-based segmentation. Eventual holes in objects were filled. Objects touching the border were not excluded. To verify whether the applied settings successfully identified cell bodies, the segmenting results can be visualized using the *DisplayDataOnImage* module.

The cytospin procedure implicates the occasional burst of cell bodies while the cell nucleus remains unharmed. Those cells can be excluded from downstream analysis based on VIME staining intensity or shape. Therefore, intensity and shape parameters of the identified secondary objects were calculated in the *MeasureObjectIntensity and MeasureObjectSizeShape* module, respectively [29, 39, 40]. We then filtered for objects with a minimum FormFactor of 0.35 and a minimum VIME MeanIntesity of 0.000009 in the *FilterObject* module that resulted in the successful segmentation of VIME^+^ cells.

### Visualization of data

CellProfiler provides a user-friendly visualization of calculated object parameters. A consecutive numbering of objects exhibiting certain parameters, e.g. real nuclei, SOX10^+^ real nuclei and SOX10^+^/EdU^+^ real nuclei, can be displayed on the images using the *DisplayDataOnImage* module. In addition, classification of objects according to positive or negative parameters in the *ClassifyObjects* module visualizes parameters using a colour code, e.g. real cells with SOX10^+^/EdU^-^ nuclei in green and SOX10^+^/EdU^+^ nuclei in red.

### Data export

The images of respective analysing steps and preferred output images such as achieved object classification can be saved in the *SaveImage* module. We exported the final counts of parameters of interest 1) *real cells* (intact cells), 2) *SOX10*^*+*^ *real cells* (SCs) and 3) *SOX10*^*+*^*/EdU*^*+*^ *real cells* (proliferating SCs) to a.txt format using the *ExportToSpreadSheet* module.

### Statistical analysis

GraphPad Prism7 was used for statistical analysis. A non-parametric analysis using paired t-test was applied to compare the data obtained from CellProfiler and from manual counting (n = 3). A p-value <0.05 was considered significant.

## Results

### Establishment of a multicolour staining panel for SC identity and proliferation suitable for automated image analysis

**Fig 1:**
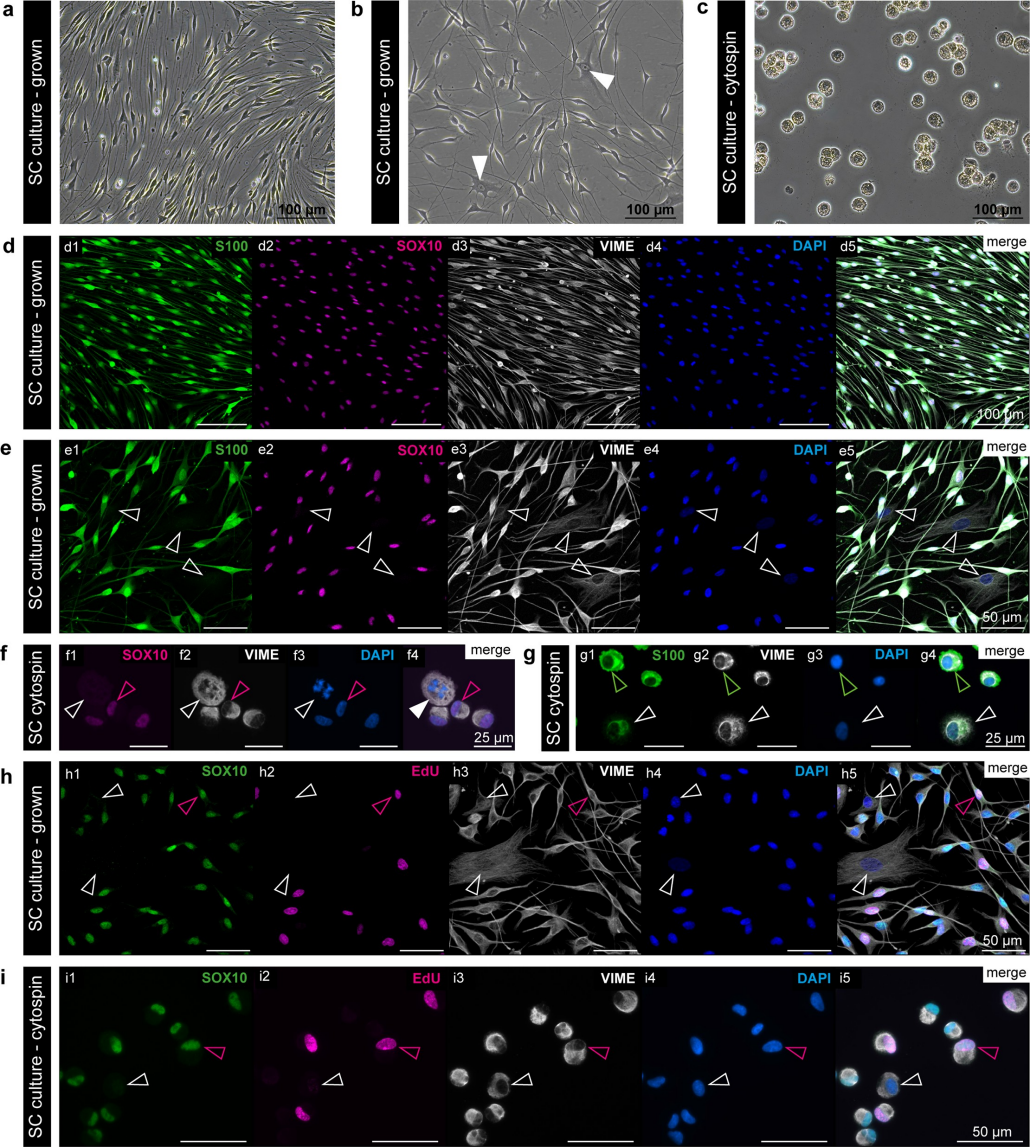
Evaluation of SC and proliferation markers on grown SC cultures and cytospins. (a,b) Phase contrast images of representative rat SC cultures with spindle-shaped SCs and flattened FBs (arrowheads). (c) Phase contrast image of a SC cytospin. (d,e) Immunofluorescence staining of grown SCs for (d1,e1) S100, (e2,e2) SOX10, (d3,e3) VIME, (d4,e4) DAPI and (d5, e5) merged channels; arrowheads mark S100^-^/SOX10^-^/VIME^+^ FBs. (f) Representative cytospin image of SCs stained for (f1) SOX10, (f2) VIME, (f3) DAPI and (f4) merged channels; magenta arrowheads point to a SOX10^+^ cell nucleus, white arrowheads indicate a SOX10^-^ mitotic cell. (g) Representative cytospin image of SCs stained for (g1) S100, (g2) VIME, (g3) DAPI and (g4) merged channels; green arrowheads show a S100^+^/VIME^+^ SC, white arrowheads refer to a VIME^+^ FB with S100 background staining. Immunofluorescence staining of (h) grown SCs and (i) SC cytospins for (h1,i1) SOX10, (h2,i2) EdU, (h3,i3) VIME, (h4,i4) DAPI and (h5, i5) merged channels; white arrowheads mark SOX10^-^/VIME^+^ FBs, magenta arrowheads mark SOX10^+^/EdU^+^/VIME^+^ proliferating SCs.

Cultured rat SCs show the typical spindle-shaped morphology with bi- to multipolar cellular projections (Fig 1A). Although SC cultures are enriched from FBs during passaging, some FBs remain in the cultures and usually exhibit a broad and flattened morphology (Fig 1B, arrowheads). To develop a multicolour staining panel for the determination of SC culture purity and proliferation, SCs were cultured in 8-well chamber slides or underwent cytocentrifugation to prepare cytospins (Fig 1C). For the confirmation of SC identity, we compared the SC associated markers, i.e. protein S100 and transcription factor SOX10, and also co-stained for the intermediate filament vimentin (VIME), expressed by both SCs and FBs. While SOX10 expression was confined to the SC nuclei, S100 signals were found in the SC nuclei and the SC cytoplasm (Fig 1D). VIME expression visualized the long SC processes that tend to align with increased culture time (Fig 1D). To validate whether S100 and SOX10 are specific for SCs, an unpurified SC culture was stained for respective markers. Indeed, FBs were negative for both SOX10 and S100 but positive for VIME (Fig 1E, arrowheads). Next, the SC culture cytospins were either stained for SOX10 and VIME (Fig 1F) or S100 and VIME (Fig 1G) to determine which SC marker is superior for following automated image analysis. On the one hand, we found that not only FBs but also all mitotic cell nuclei lack the expression of SOX10 (Fig 1F, white arrowhead) indicating its downregulation during cell division. On the other hand, we regularly observed a considerable amount of S100 background staining in cytospun FBs (Fig 1G, white arrowhead). To avoid false positive SCs and due to the clear nuclear confinement of fluorescence signals, SOX10 was chosen as SC marker for further analysis.

In a next step, we implemented information about the cellular proliferation status in our staining panel by using EdU as proliferation marker. Therefore, the thymidine analogue EdU was added to the SC cultures for 2 hours. During that time EdU was incorporated in every cell that is synthesizing DNA. Then, grown SCs or prepared SC cytospins were stained for the established multi-colour staining panel including SOX10, EdU, VIME and DAPI (Fig 1H and 1I, respectively). The EdU staining resulted in clear nuclear signals without background (Fig 1H2 and 1I2). Compared to the grown cultures, cytospun cells were more easily distinguishable due to the homogeneous appearance of cells, which encouraged their suitability for automated image analysis.

### Development of an automated image analysis pipeline to determine the purity and proliferation rate of SC cultures

**Fig 2:**
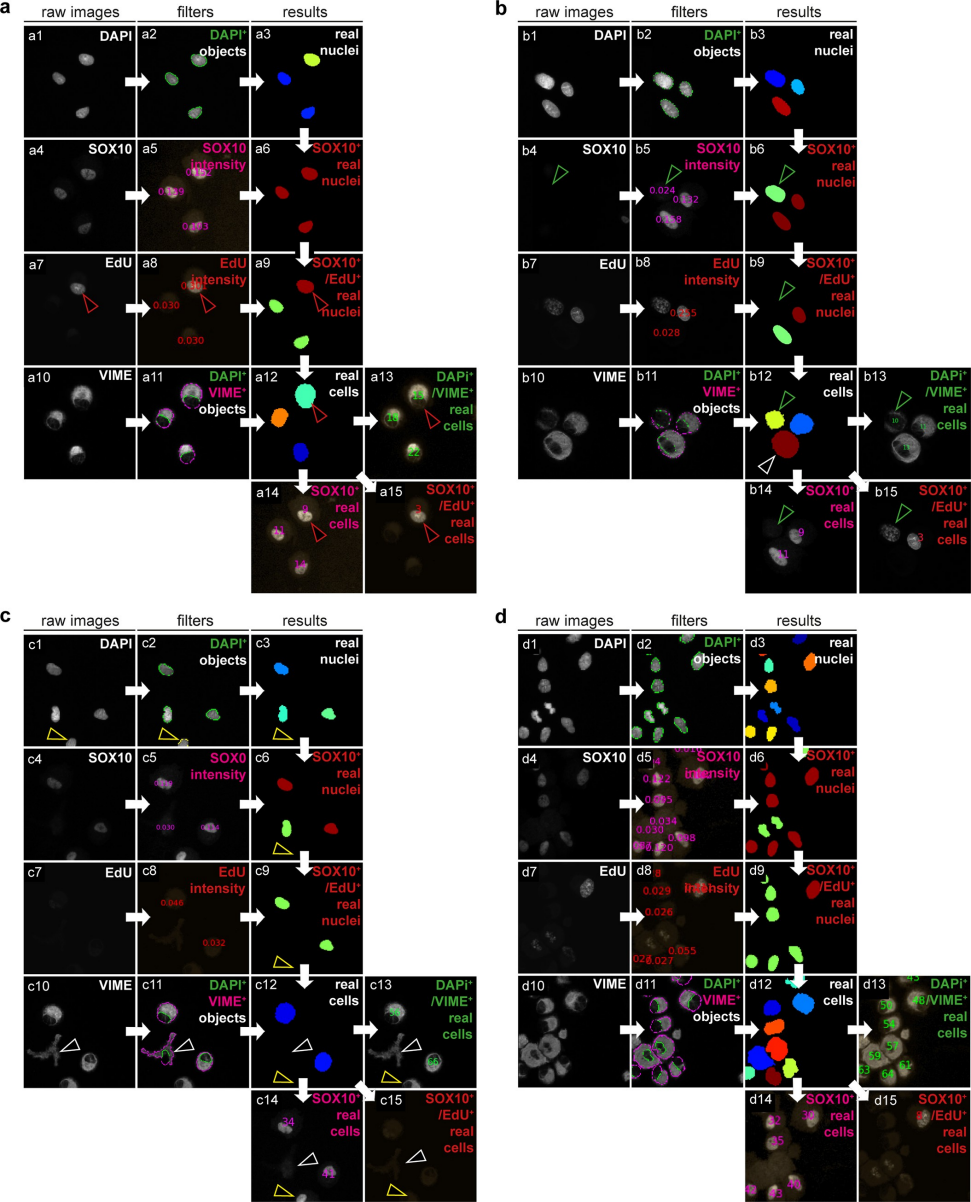
Overview of the automated image analysis segmenting results. (a-d) illustrate representative image analysis steps of the established pipeline using CellProfler. (a-d, 1–3) show the identification of real nuclei based on the DAPI stain, (a-d, 4–6) illustrate the determination of SOX10^+^ real nuclei and (a-d, 7–9) the determination of SOX10^+^/EdU^+^ real nuclei based on the SOX10 and EdU staining intensities, respectively. The VIME stain was used to exclude burst cell bodies and to identify *real cells* (a-d, 9–12) from which the number of *real cells* (a-d, 13), *SOX10*^*+*^ *real cells* (a-d, 14) and *SOX10*^*+*^*/EdU*^*+*^ *real cells* (a-d, 15) were determined.

In order to quantify the number of SCs and proliferating SCs on cytospins co-stained for SOX10, EdU, VIME and DAPI, we adapted the automated image analysis pipeline provided by the CellProfiler software (S1 File). Therefore, raw images of each staining channel were sequentially filtered according to certain processing modules (Table 2). The main image analysis steps and four representative segmenting results of the pipeline are depicted in Fig 2A–2D. Intact (real) cell nuclei were identified by segmenting DAPI^+^ objects and filtering according to established size, shape, and form parameters to exclude burst cell nuclei (Fig 2A–2D, 1–3). The pipeline then analysed the SOX10 stain images and displayed the mean SOX10 staining intensities of identified *real* cell nuclei (Fig 2A–2D, 4–5). This enabled the manual definition of a threshold for SOX10 positivity to determine SOX10^+^ real cell nuclei (Fig 2A–2D, 6). The same procedure was applied to assess EdU positivity and determine SOX10^+^/EdU^+^ real cell nuclei (Fig 2A–2D, 7–9). As some cells do burst during the cytocentrifugation procedure, we also implemented a quality control step to exclude cell nuclei without a cell body or with a deformed cell body. Intact (real) cells were identified by segmenting VIME^+^ objects (cell bodies) associated with a real cell nucleus and filtering according to established size, shape, form, and intensity parameters (Fig 2A–2D, 10–12). The pipeline then quantified the number of *VIME*^*+*^ *cells* (real cells) (Fig 2A–2D, 13), *SOX10*^*+*^ *real cells* (SCs) (Fig 2A–2D,14) as well as *SOX10*^*+*^*/EdU*^*+*^ *real cells* (proliferating SCs) (Fig 2A–2D, 15) and exported the results to a spreadsheet.

### Validation of the established CellProfiler pipeline

**Fig 3:**
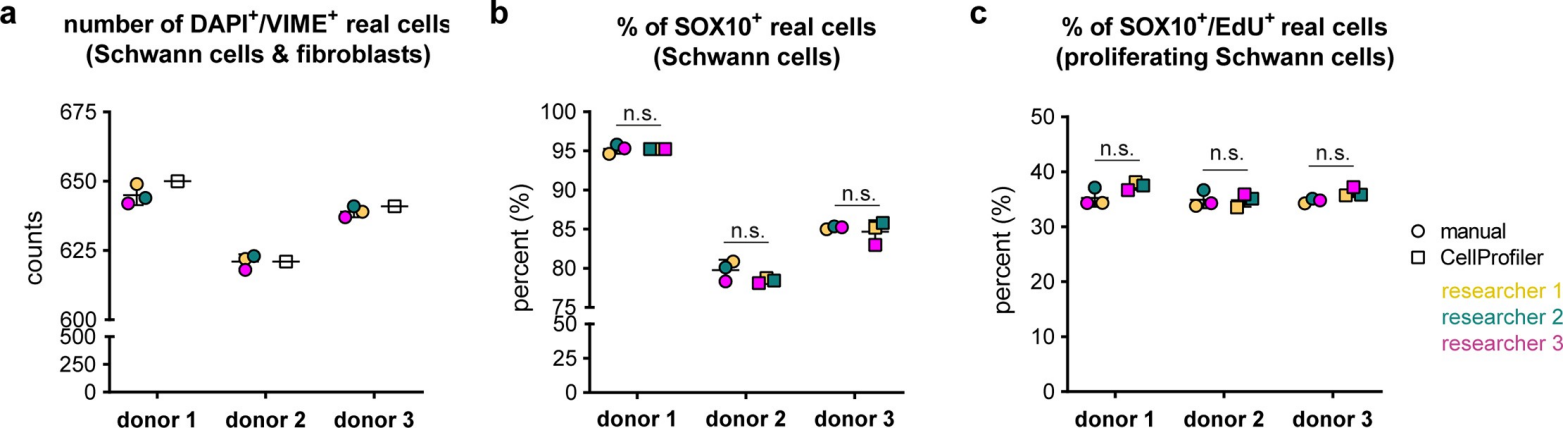
Comparison of CellProfiler and manual counting results. The diagrams show the calculated (a) numbers of DAPI^+^/VIME^+^ *real cells*, (b) percentages of *SOX10*^*+*^ *real cells* (= SC culture purity) and (c) percentage of *SOX10*^*+*^*/EdU*^*+*^ *real cells* (= proliferating SCs) obtained by either manual counting (circles) or the CellProfiler pipeline (squares) ±SD in 3 donors; n.s. … not significant.

To validate the CellProfiler pipeline, we compared the CellProfiler results with those obtained by manual counting. Therefore, stained SC culture cytospins of three donors were analysed by the CellProfiler pipeline and by three independent researchers for 1) *real cells* (DAPI^+^/VIME^+^), 2) *SOX10*^*+*^ *real cells* (= SCs) and 3) *SOX10*^*+*^*/EdU*^*+*^ *real cells* (= proliferating SCs). To challenge the applicability of the pipeline, each researcher independently determined a threshold for SOX10 and EdU positivity. The filter parameters for *real cells* based on the VIME stain remained unchanged. We found that the amount of *real cells* per donor identified by the CellProfiler pipeline was highly concordant to those identified by the 3 researchers (Fig 3A). Further, the results of both methods showed no significant differences in the determined SC culture purity calculated as percent of *SOX10*^*+*^ *real cells* (Fig 3B). Manual and CellProfiler results also demonstrated similar results for the SC proliferation rate calculated as percent of *SOX10*^*+*^*/EdU*^*+*^ *real cells* and revealed a consistent SC proliferation rate of about 35% in all three donors (Fig 3C). These results illustrate the suitability and applicability of the established CellProfiler pipeline for automated quantification of SC purity and proliferation of stained cytospins.

## Discussion

SC cultures derived from nerves, skin or tumour biopsies are highly valuable to study molecular mechanisms involved in repair or disease as well as to validate their response to novel treatments *in vitro*. Morphological assessment of SC cultures enables a brief overview about the number of contaminating FBs but a reliable evaluation demands the verification of SC associated markers. Alongside with purity, the proliferation status of SCs is an important indicator of the culture condition. Flow cytometry or immunocytochemistry are commonly used to retrieve information about the purity and proliferation rate of SC cultures. Flow cytometry is a precise method to quantify marker expression but requires about 1x10^5^ cells for proper analysis. Considering that a 6-well at 80% confluency contains about 1x10^6^ rat SCs, a tenth of the culture would have to be sacrificed. For immunofluorescence analysis, it is sufficient to grow an aliquot of about 1x10^4^ SCs in a well of an 8-well chamber slide parallel to the main culture. This sister culture is expected to reflect the main culture characteristics, however, differences cannot be excluded.

Aiming to reduce the number of SCs needed for the determination of purity and proliferation, we used cytocentrifugation, a method for fast and easy preparation of microscopic specimens from cell suspensions [41, 42]. Notably, only about 1x10^4^cells are required per cytospin. This method further enables the analysis of the main SC culture properties, for example, during passaging, after enrichment or after experimentation. Another advantage is the short to long term storage of derived cytospin slides at -20°C, which allows preserving information about the culture. In this way culture characteristics such as the protein expression status can be retrieved months after the actual culture by immunostainings. Indeed, morphological features of adherent cells are lost during the cytospin procedure. However, the uniformly rounded shape and separation of cells facilitate marker expression analysis on a single cell level.

For the establishment of an immunofluorescence staining panel including a SC marker and a proliferation marker, we first compared two intracellular SC associated proteins, the S100 calcium binding protein B (S100) and the transcription factor SOX10 [43, 44]. We chose intracellular markers as permeabilization is required for the staining of proliferation marker EdU. Combining an EdU staining with an extracellular SC marker such as the tumour necrosis factor receptor superfamily member 16, also known as NGFR or p75^NTR^, would imply a sequential staining procedure causing increased hands-on time. After S100 and SOX10 validation, we found that the clear, nuclear SOX10 staining is preferable to S100, as S100 can cause high background signals in cytospun FBs. It shall be noted that the cytospin procedure sometimes lead to the burst of cells. To filter for cells with an intact cell body, we included the intermediate filament VIME in our staining panel, which successfully provided information about the cytoskeletal (cell body) integrity.

Next, we replaced the tedious and time-consuming manual counting of (proliferating) SCs by an algorithm based method. Cytospins have previously been demonstrated to be suitable for automated image analysis using Matlab-based custom-made pipelines [20, 45]. To provide a public available pipeline for the quantification of SC purity and proliferation that is independent of programming skills, we here decided to use the open-source software CellProfiler [29]. Immunofluorescence images of rat SC culture derived cytospins stained for SOX10, EdU, VIME and DAPI served as input for CellProfiler analysis. The established pipeline identified intact cells (containing a nucleus and a cell body) present in the images and calculated cellular features according to staining intensity, size and shape. Comparison of the total cell count, SC purity, and SC proliferation rate between manual counting (gold standard) and the established pipeline showed no significant differences in the three SC donors analysed. Thus, we demonstrated that the pipeline accurately identified SCs and proliferating SCs on stained cytospin slides.

In conclusion, we here provide a reliable read-out for the quantification of SC culture purity and proliferation from a small number of cells using stained cytospins combined with automated image analysis. Thus, our method saves valuable Schwann cells and facilitates to effectively study their constantly increasing roles in nerve regeneration, neuropathies, and perineural invasion of cancer cells.

## Supporting information

S1 File CellProfiler library. (CPPROJ)

S2 File (CPPROJ)

S3 File (XLSX)

S1 Data (ZIP)

S2 Data (ZIP)

S3 Data (ZIP)
