# Supplementary figures and images for: Automated image analysis of stained cytospins to quantify Schwann cell purity and proliferation
Source: PLoS One. 2020 May 22;15(5):e0233647. doi: 10.1371/journal.pone.0233647 (PMC7244157; doi:10.1371/journal.pone.0233647)

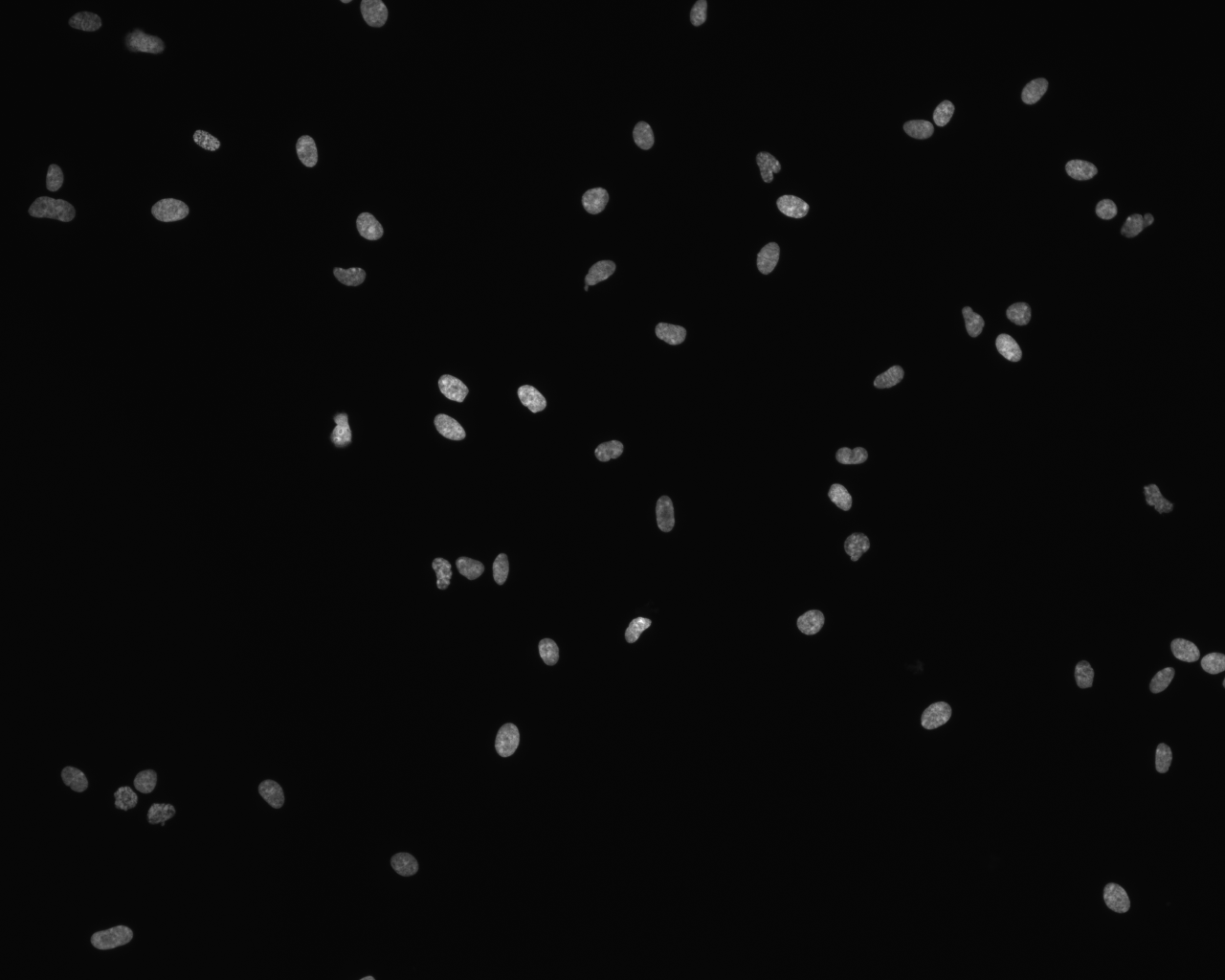

Supplement: S1 Data — (ZIP) [file pone.0233647.s004.zip › rSC48/rSC48_SOX10AF488_EdUAF555_VIMEDL650_DAPI_20x_1_DAPI 1.tif]

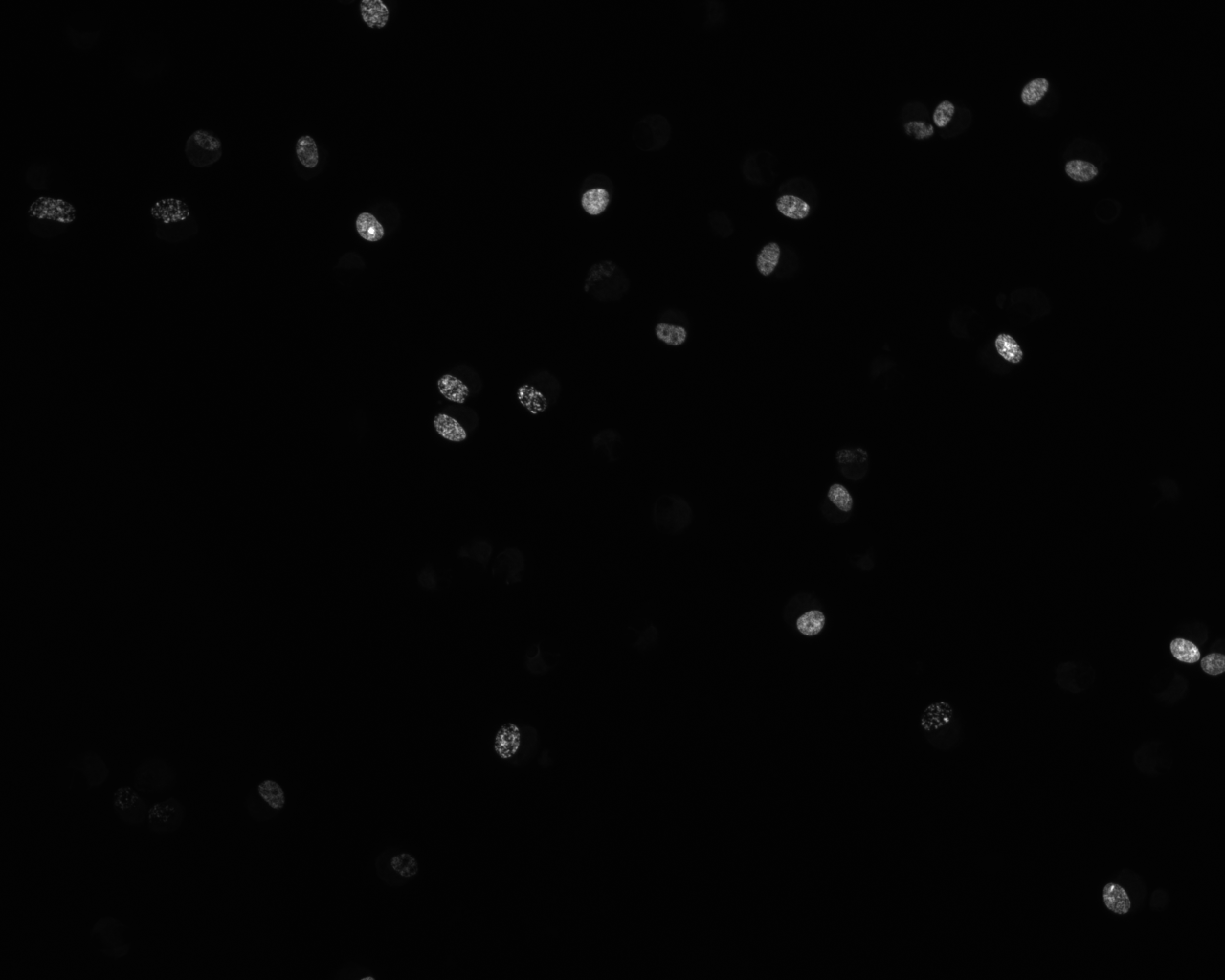

Supplement: S1 Data — (ZIP) [file pone.0233647.s004.zip › rSC48/rSC48_SOX10AF488_EdUAF555_VIMEDL650_DAPI_20x_1_EdU 4.tif]

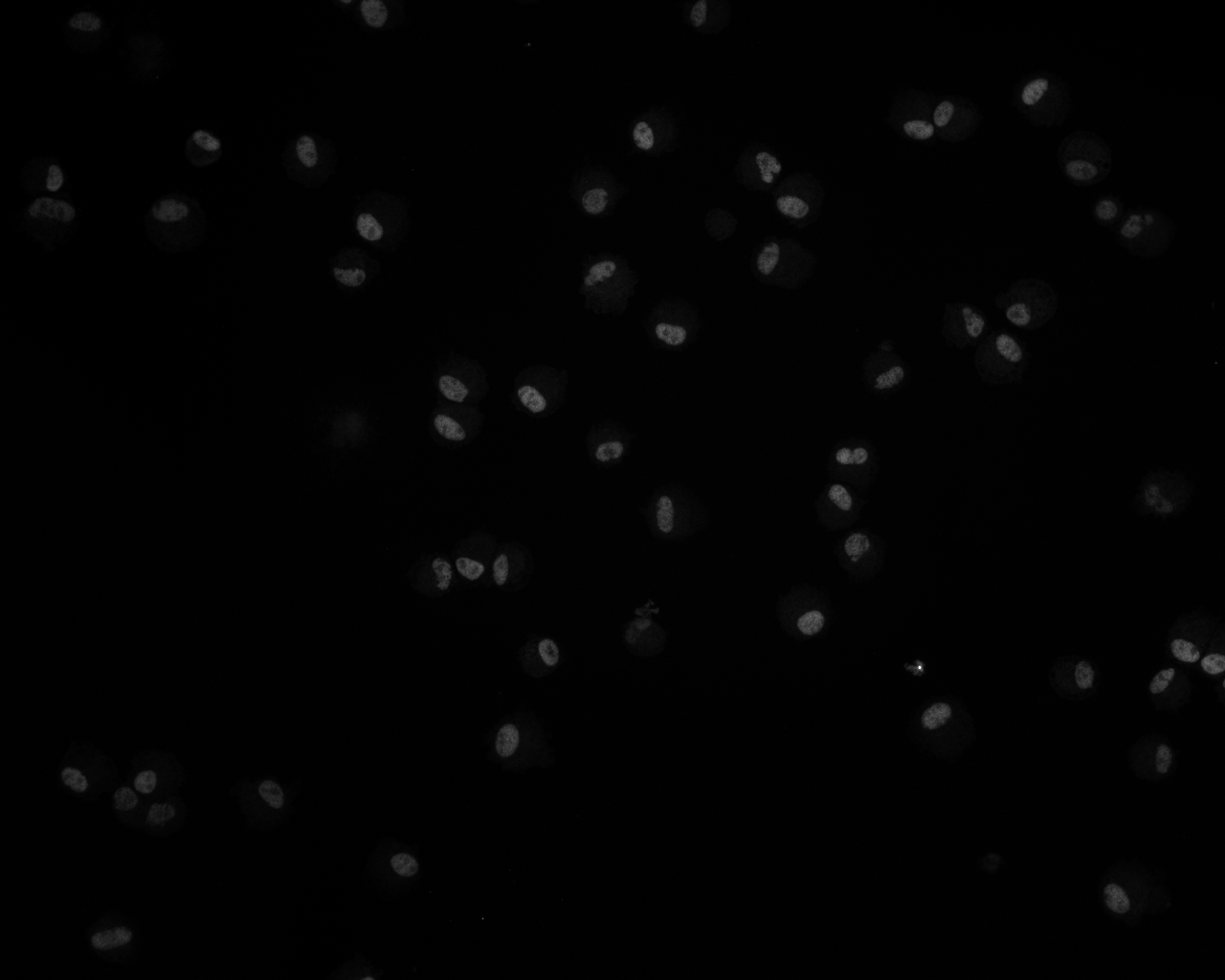

Supplement: S1 Data — (ZIP) [file pone.0233647.s004.zip › rSC48/rSC48_SOX10AF488_EdUAF555_VIMEDL650_DAPI_20x_1_SOX10 2.tif]

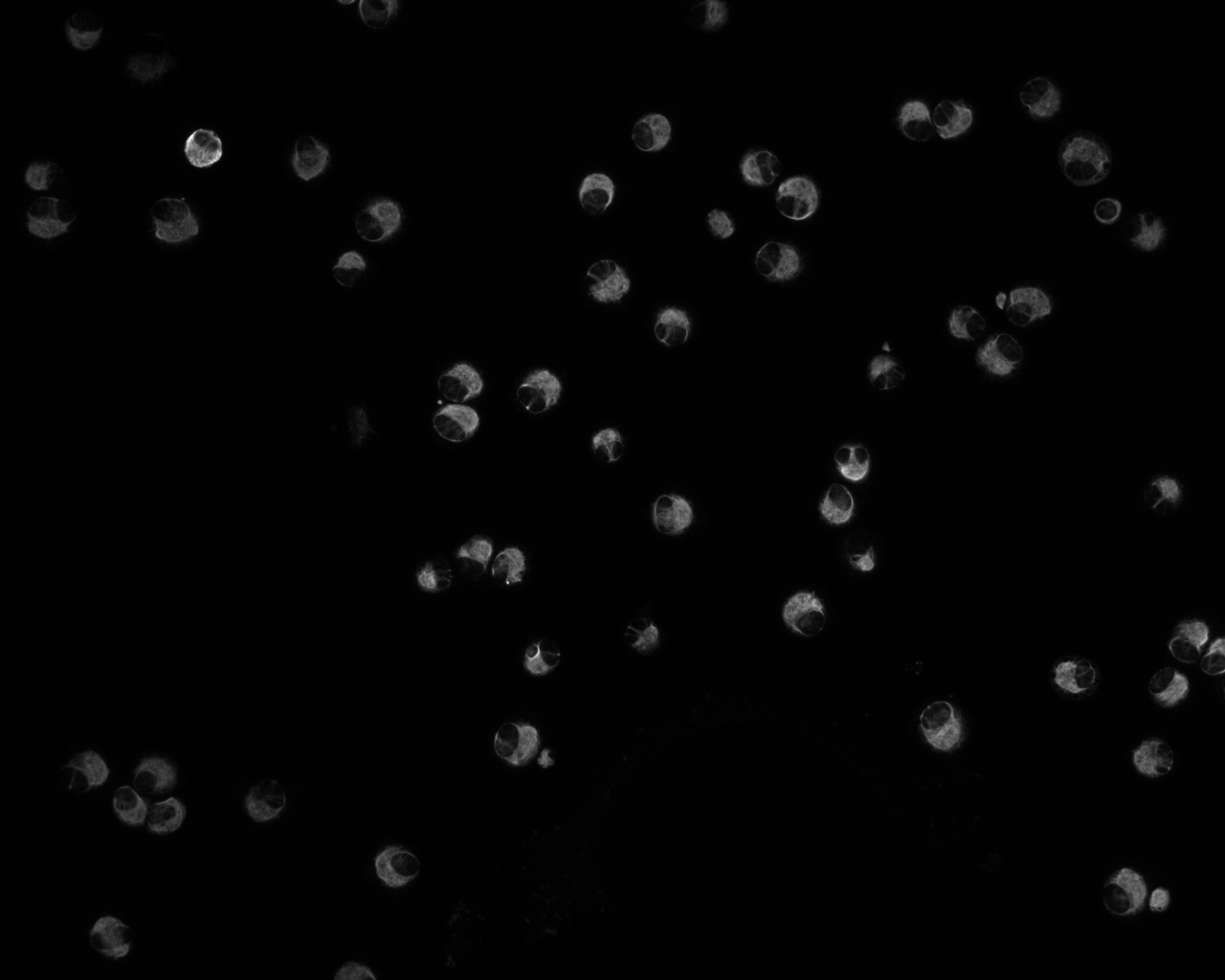

Supplement: S1 Data — (ZIP) [file pone.0233647.s004.zip › rSC48/rSC48_SOX10AF488_EdUAF555_VIMEDL650_DAPI_20x_1_VIME 3.tif]

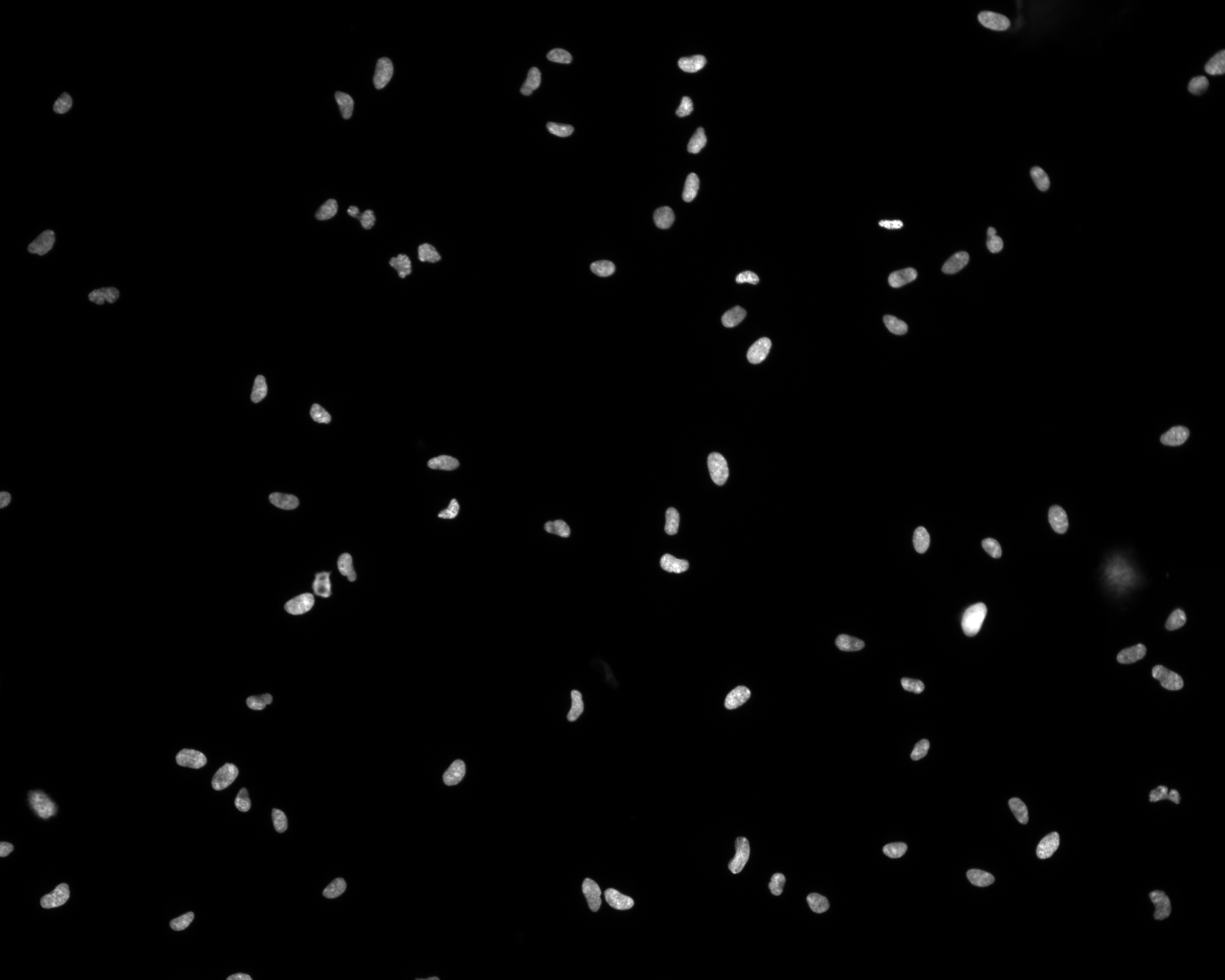

Supplement: S1 Data — (ZIP) [file pone.0233647.s004.zip › rSC48/rSC48_SOX10AF488_EdUAF555_VIMEDL650_DAPI_20x_10_DAPI 1.tif]

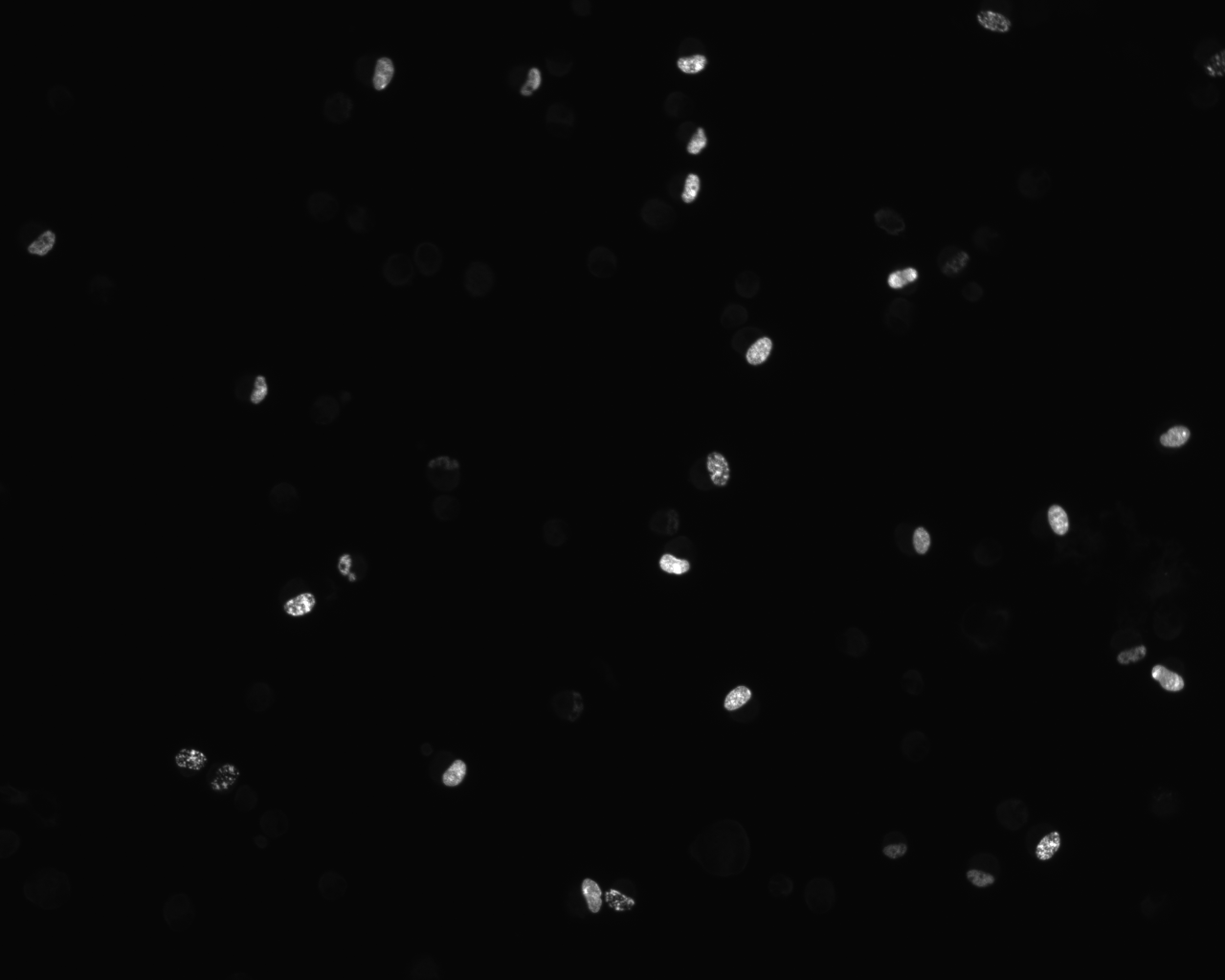

Supplement: S1 Data — (ZIP) [file pone.0233647.s004.zip › rSC48/rSC48_SOX10AF488_EdUAF555_VIMEDL650_DAPI_20x_10_EdU 4.tif]

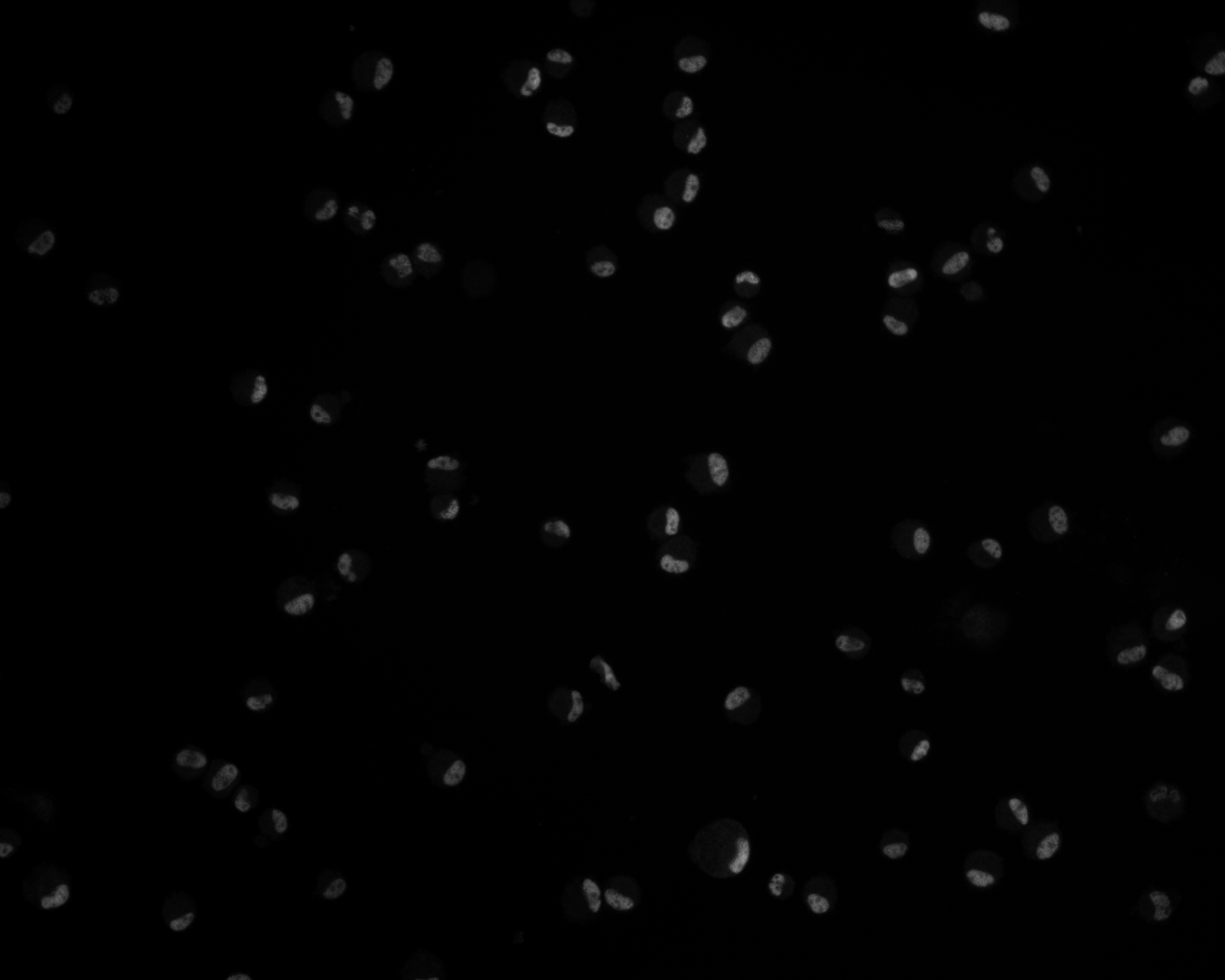

Supplement: S1 Data — (ZIP) [file pone.0233647.s004.zip › rSC48/rSC48_SOX10AF488_EdUAF555_VIMEDL650_DAPI_20x_10_SOX10 2.tif]

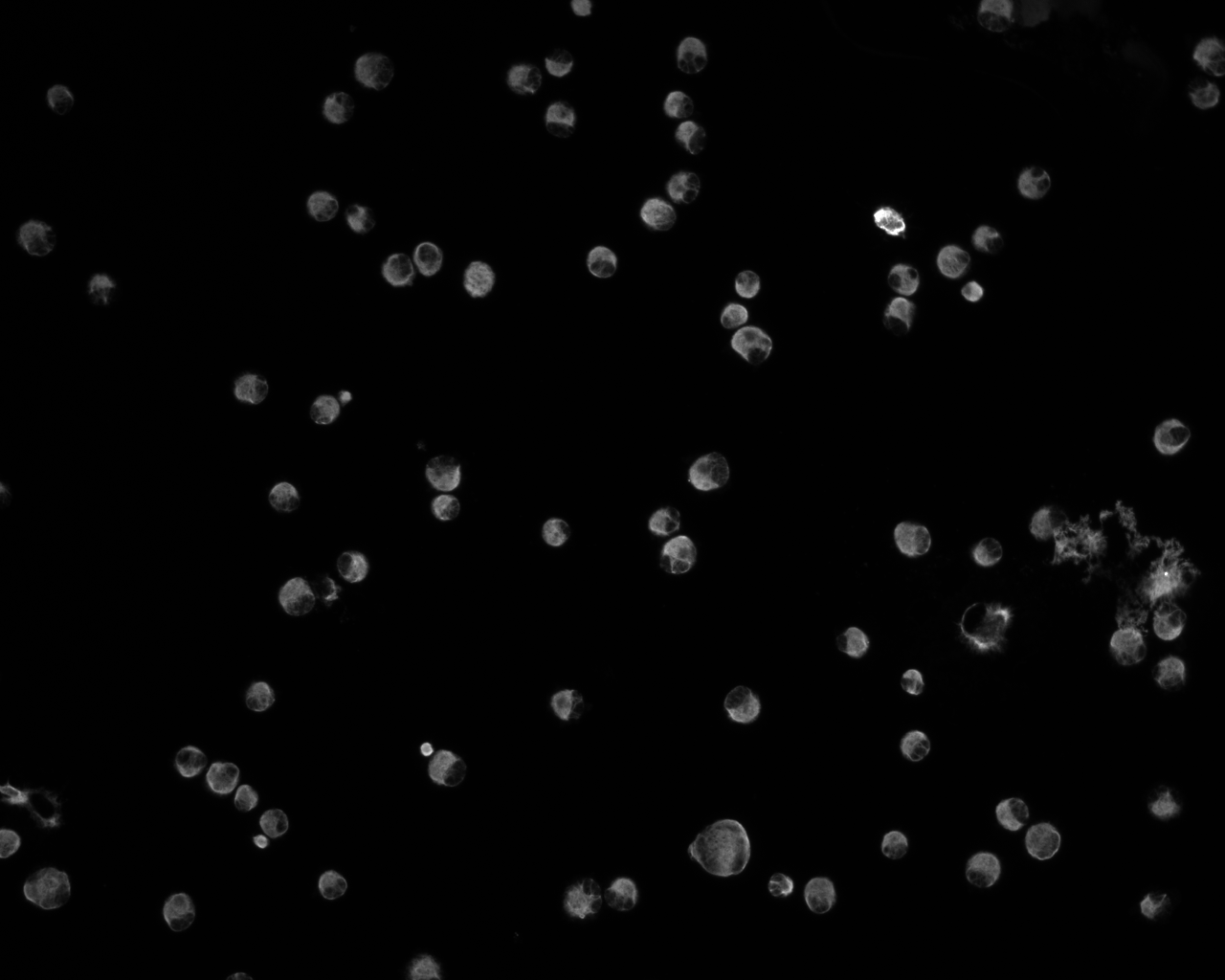

Supplement: S1 Data — (ZIP) [file pone.0233647.s004.zip › rSC48/rSC48_SOX10AF488_EdUAF555_VIMEDL650_DAPI_20x_10_VIME 3.tif]

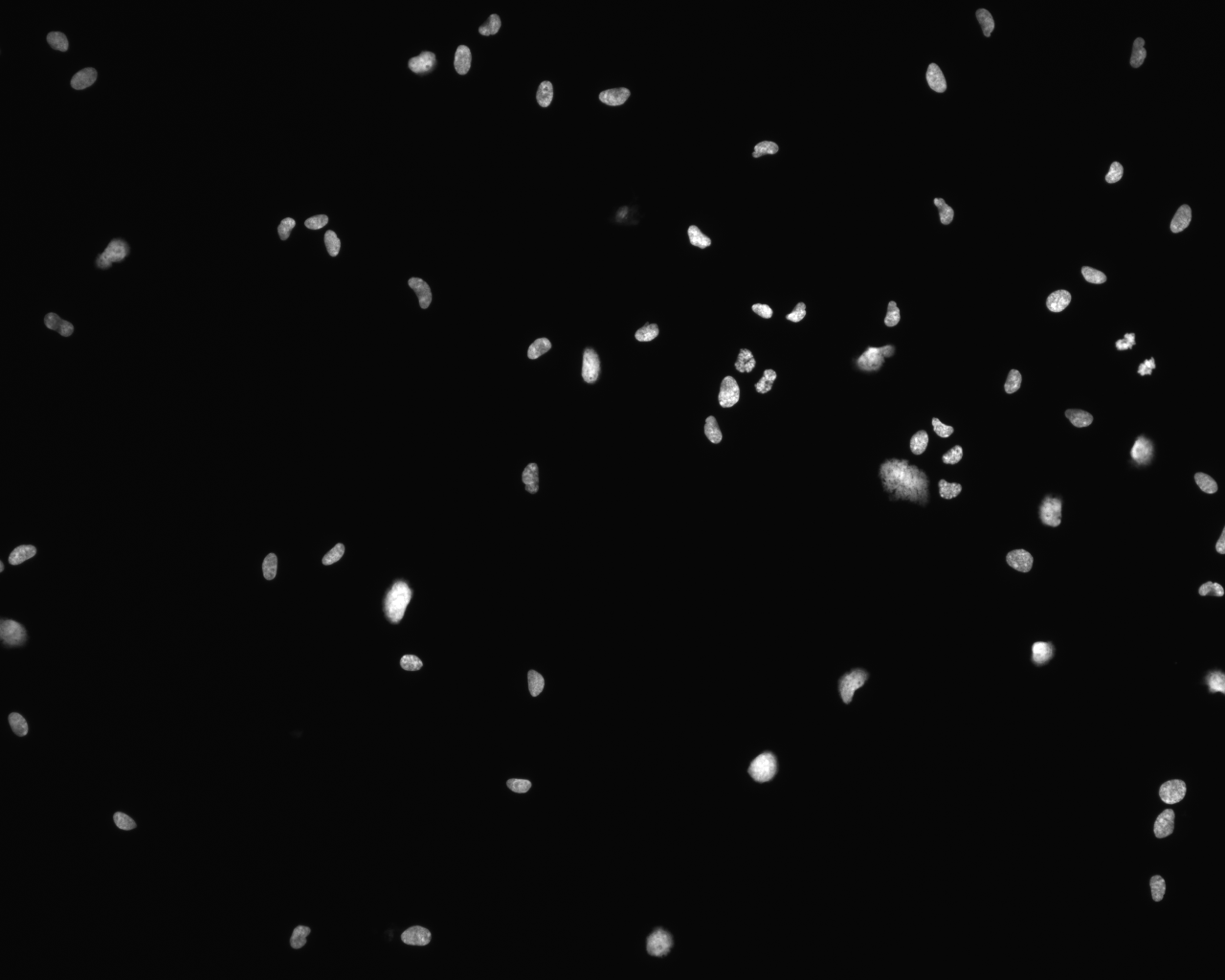

Supplement: S1 Data — (ZIP) [file pone.0233647.s004.zip › rSC48/rSC48_SOX10AF488_EdUAF555_VIMEDL650_DAPI_20x_11_DAPI 1.tif]

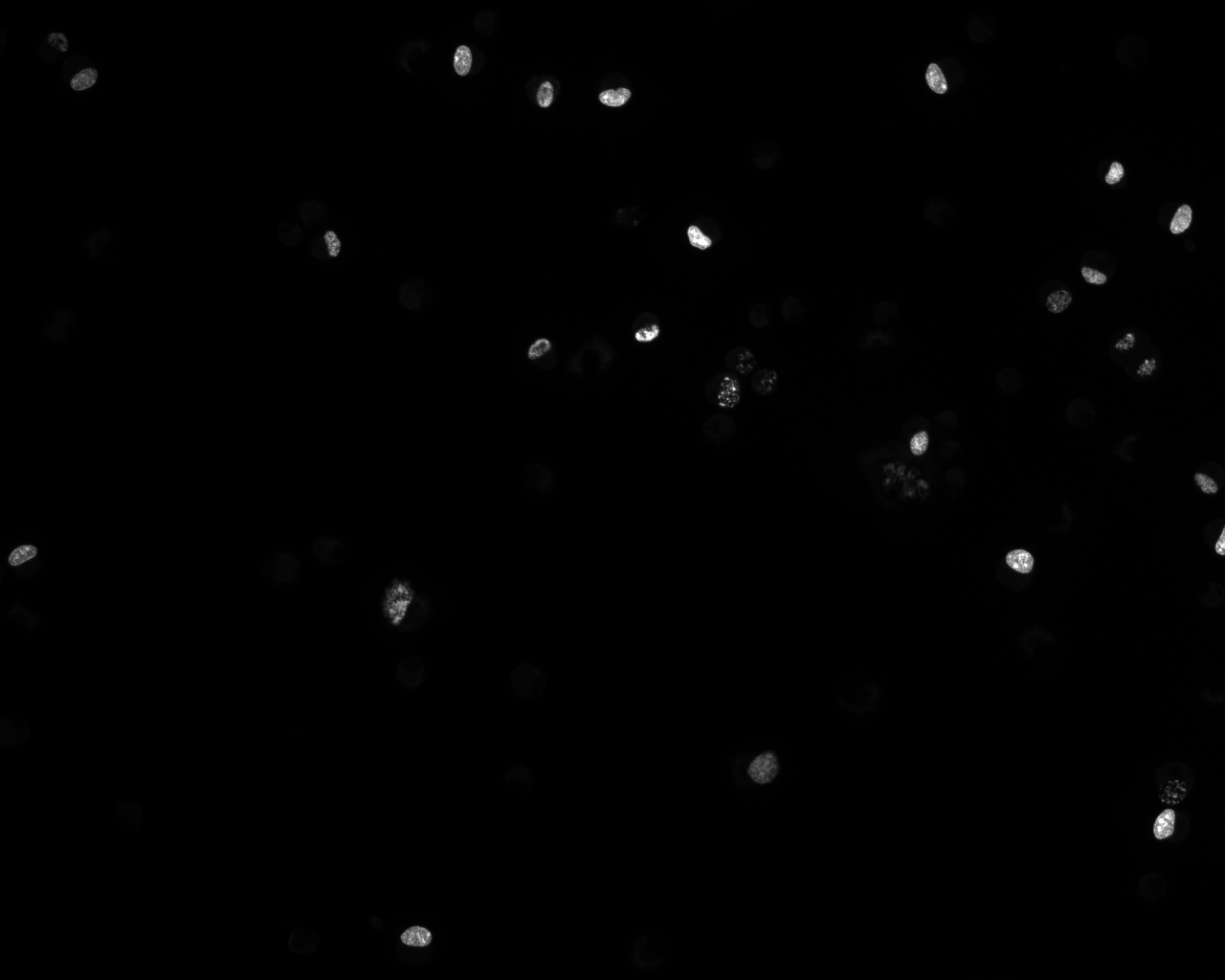

Supplement: S1 Data — (ZIP) [file pone.0233647.s004.zip › rSC48/rSC48_SOX10AF488_EdUAF555_VIMEDL650_DAPI_20x_11_EdU 4.tif]

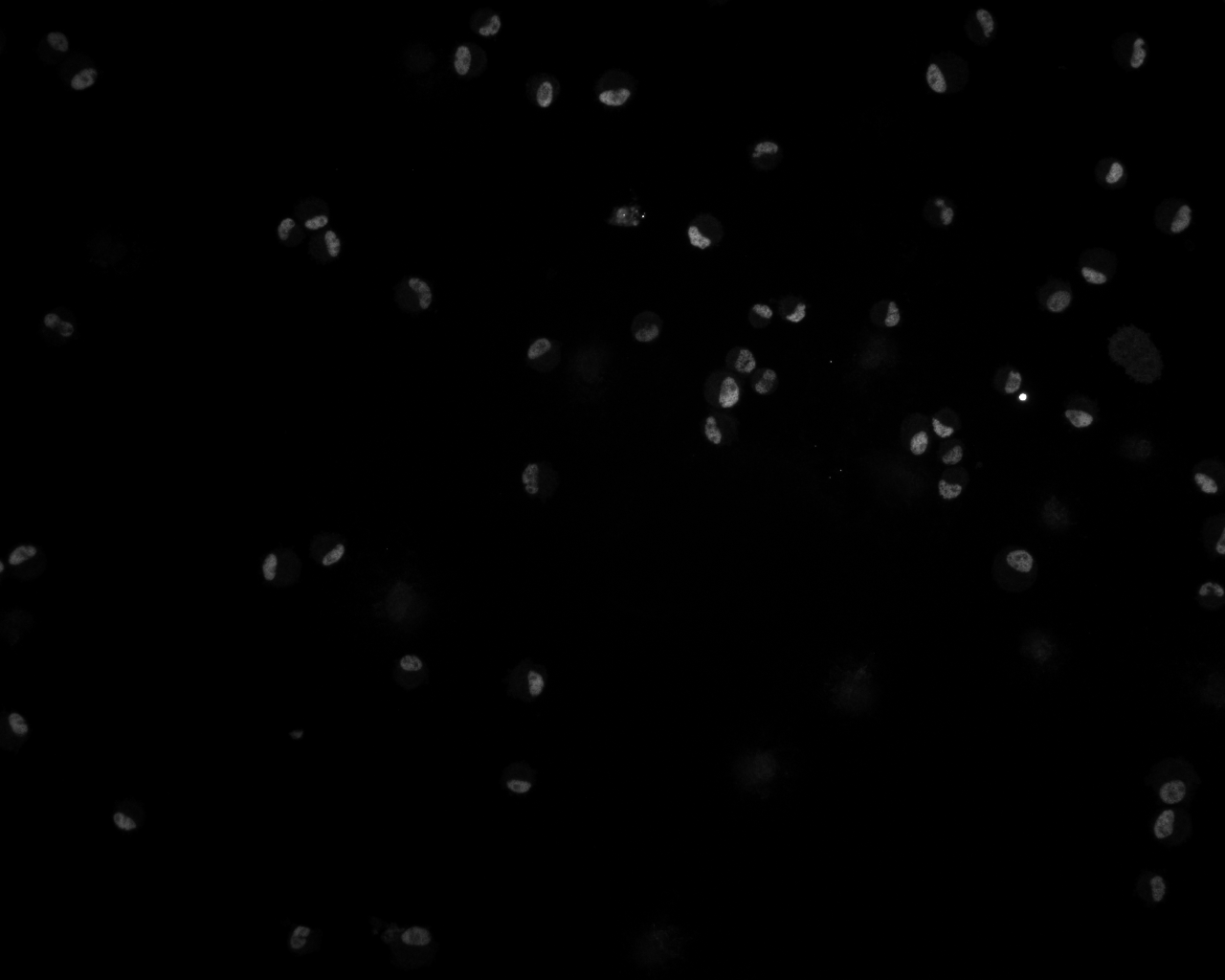

Supplement: S1 Data — (ZIP) [file pone.0233647.s004.zip › rSC48/rSC48_SOX10AF488_EdUAF555_VIMEDL650_DAPI_20x_11_SOX10 2.tif]

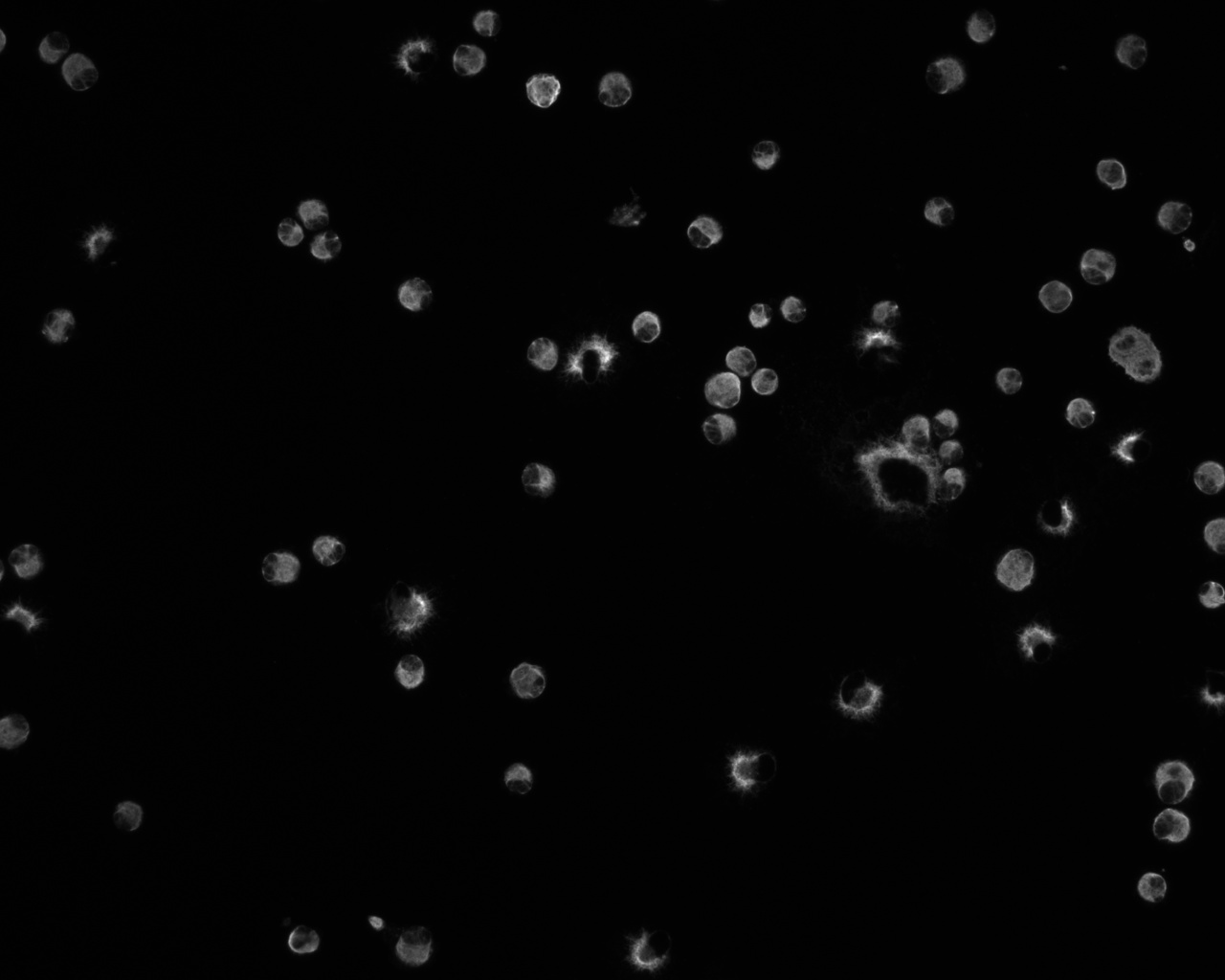

Supplement: S1 Data — (ZIP) [file pone.0233647.s004.zip › rSC48/rSC48_SOX10AF488_EdUAF555_VIMEDL650_DAPI_20x_11_VIME 3.tif]

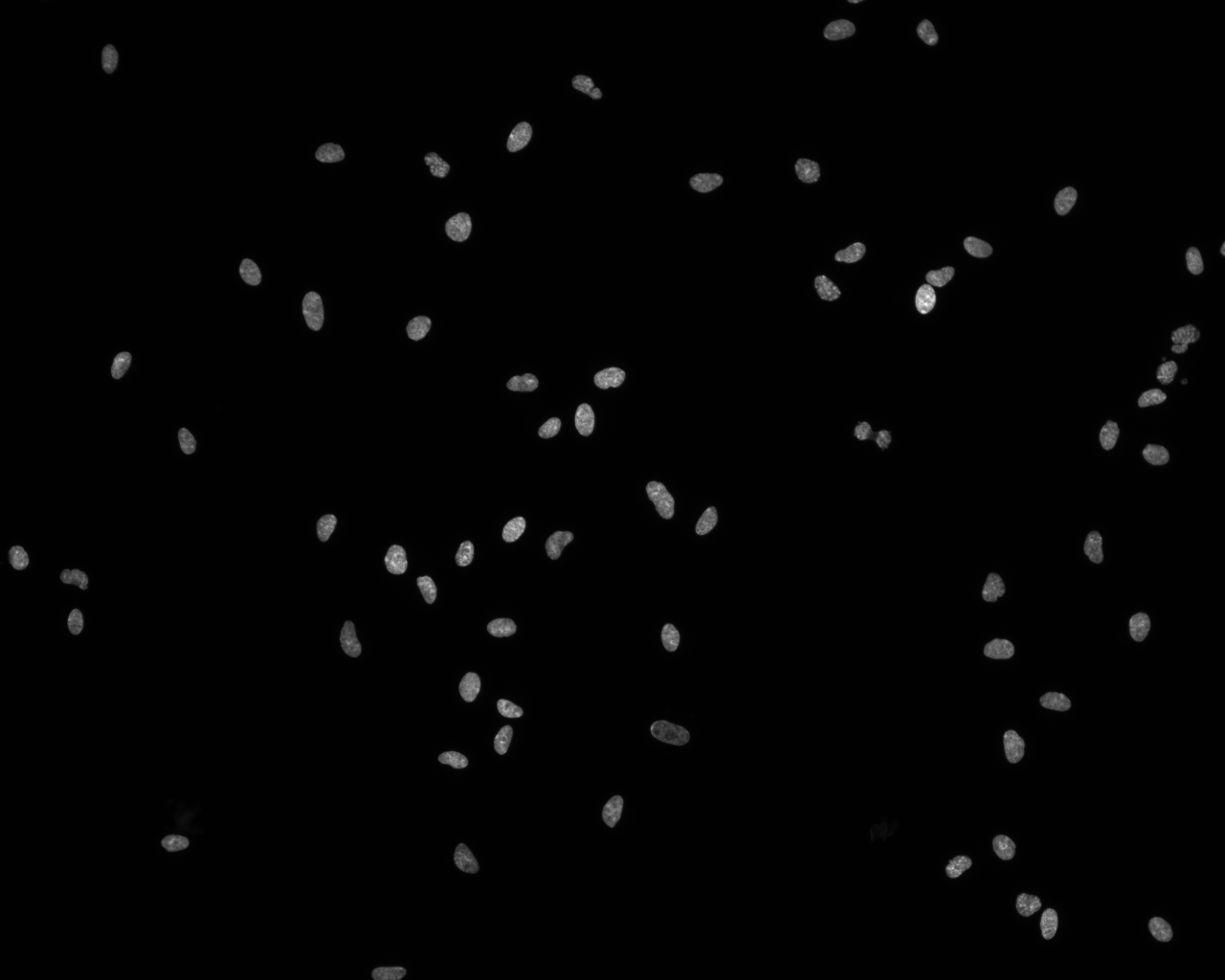

Supplement: S1 Data — (ZIP) [file pone.0233647.s004.zip › rSC48/rSC48_SOX10AF488_EdUAF555_VIMEDL650_DAPI_20x_2_DAPI 1.tif]

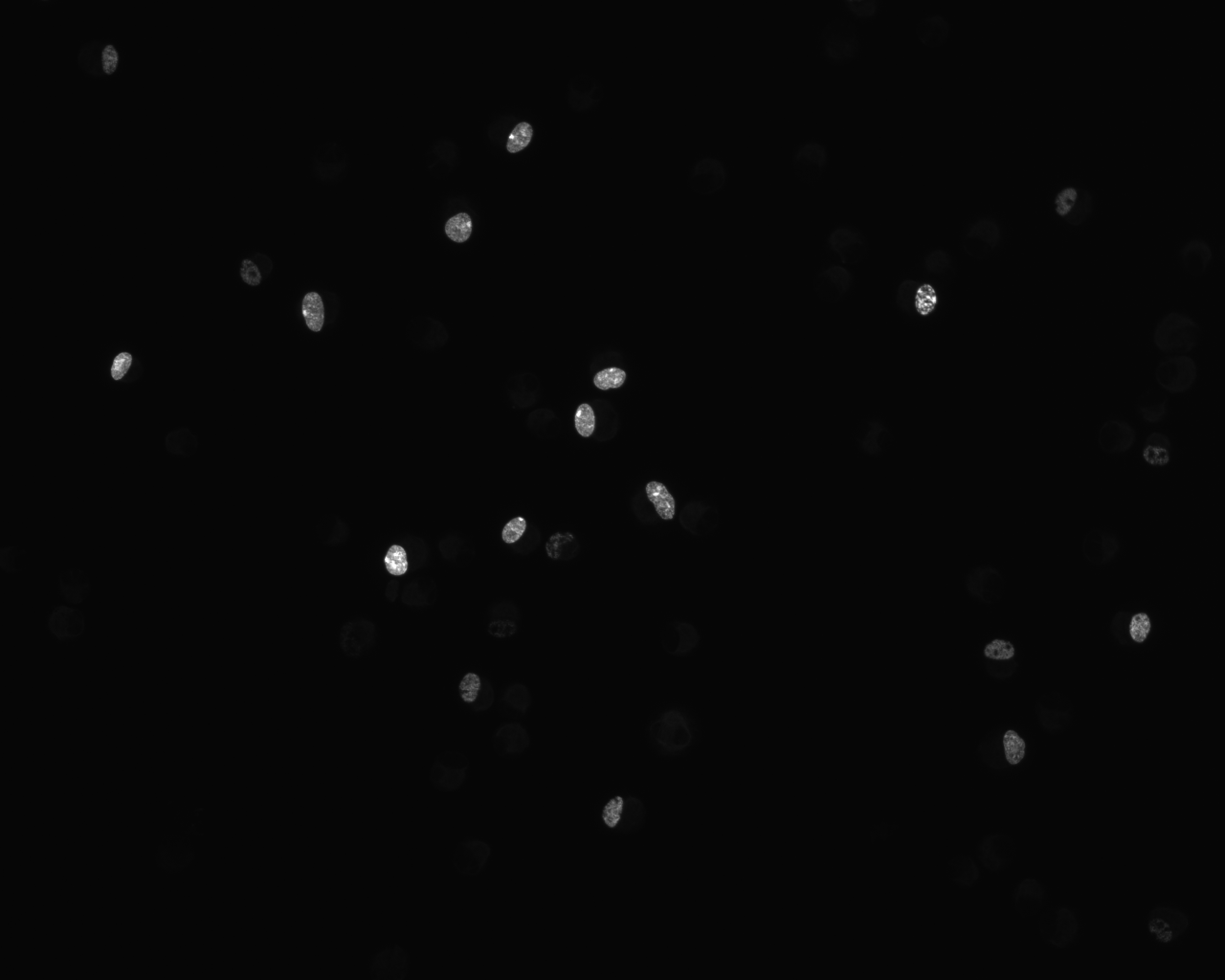

Supplement: S1 Data — (ZIP) [file pone.0233647.s004.zip › rSC48/rSC48_SOX10AF488_EdUAF555_VIMEDL650_DAPI_20x_2_EdU 4.tif]

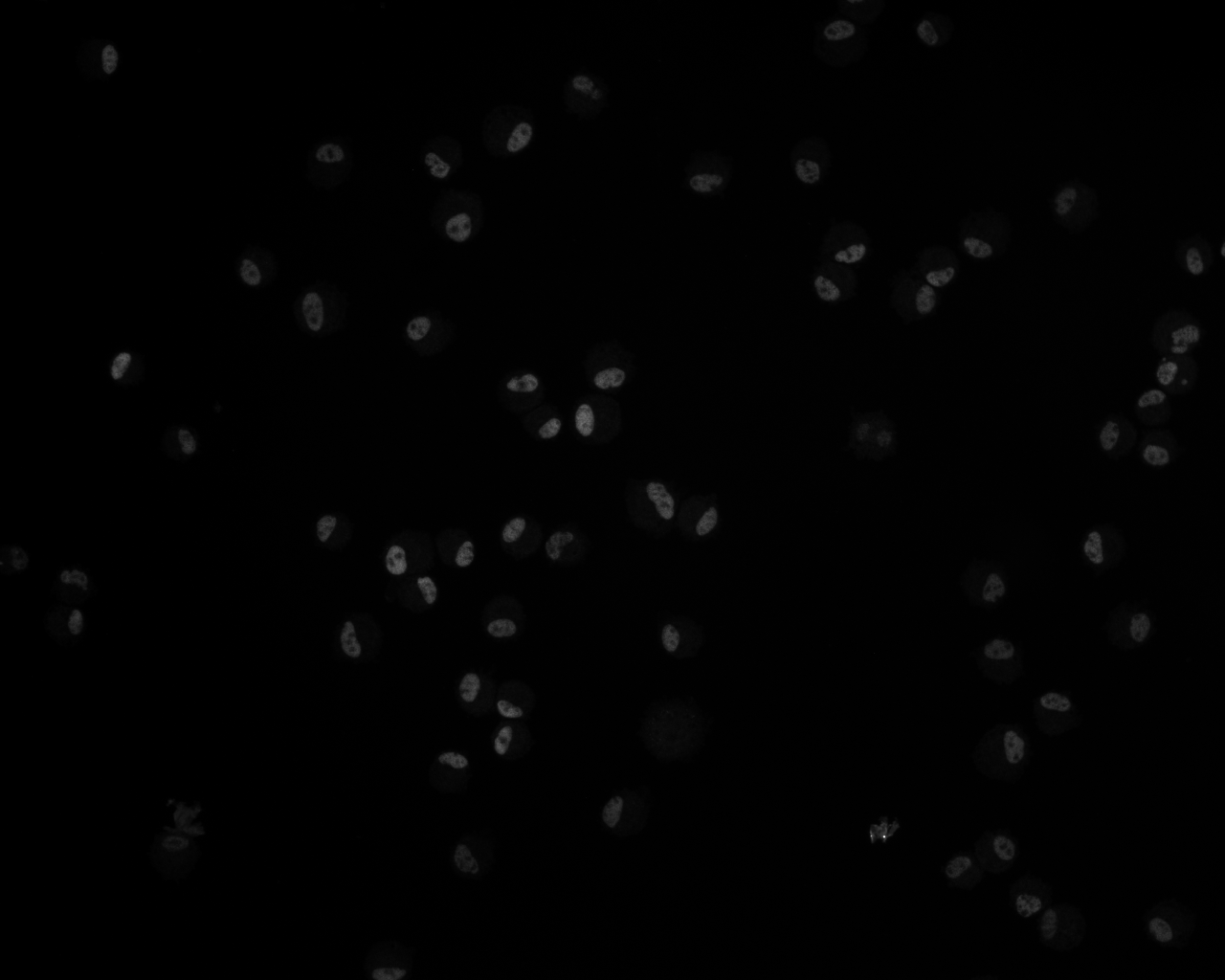

Supplement: S1 Data — (ZIP) [file pone.0233647.s004.zip › rSC48/rSC48_SOX10AF488_EdUAF555_VIMEDL650_DAPI_20x_2_SOX10 2.tif]

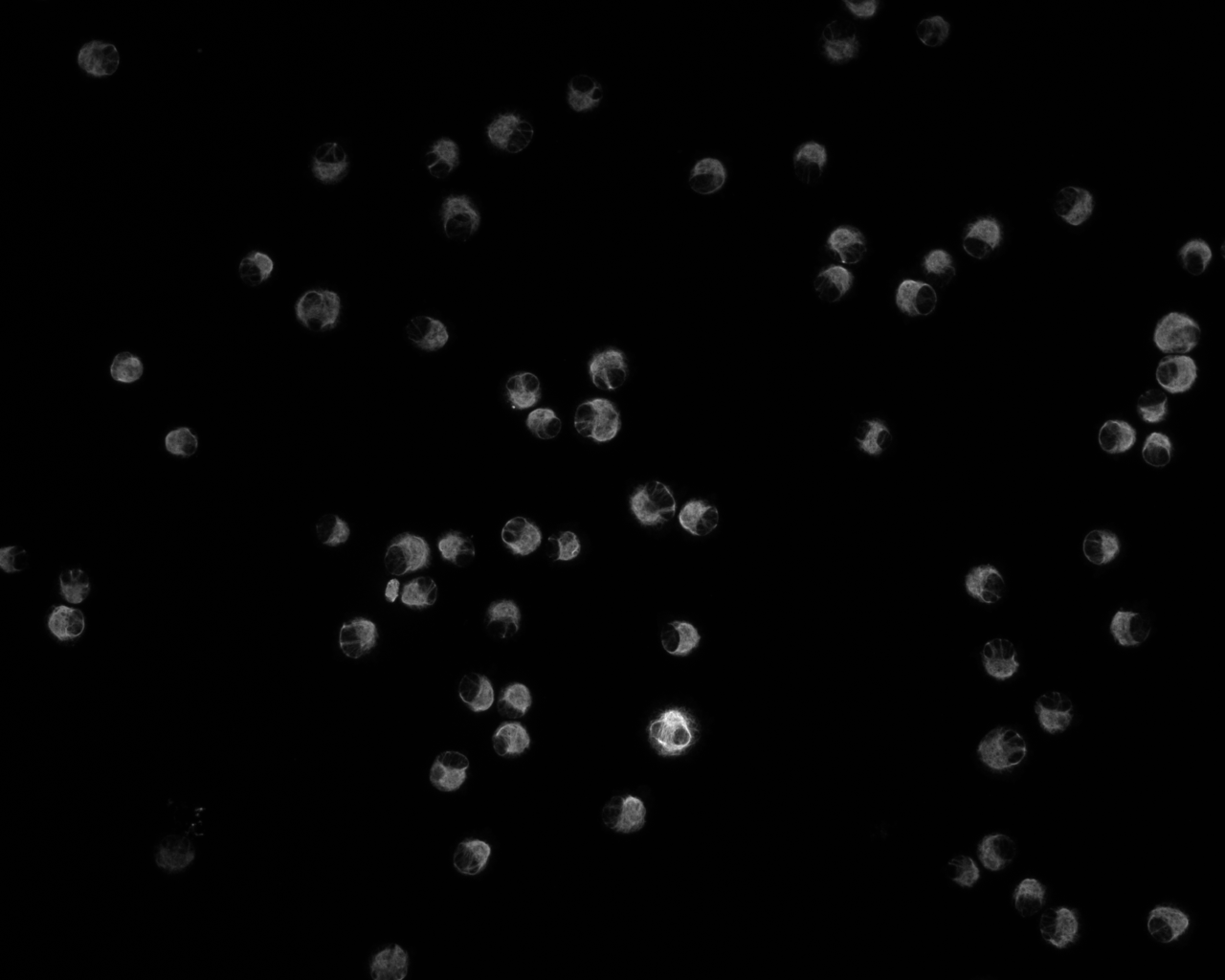

Supplement: S1 Data — (ZIP) [file pone.0233647.s004.zip › rSC48/rSC48_SOX10AF488_EdUAF555_VIMEDL650_DAPI_20x_2_VIME 3.tif]

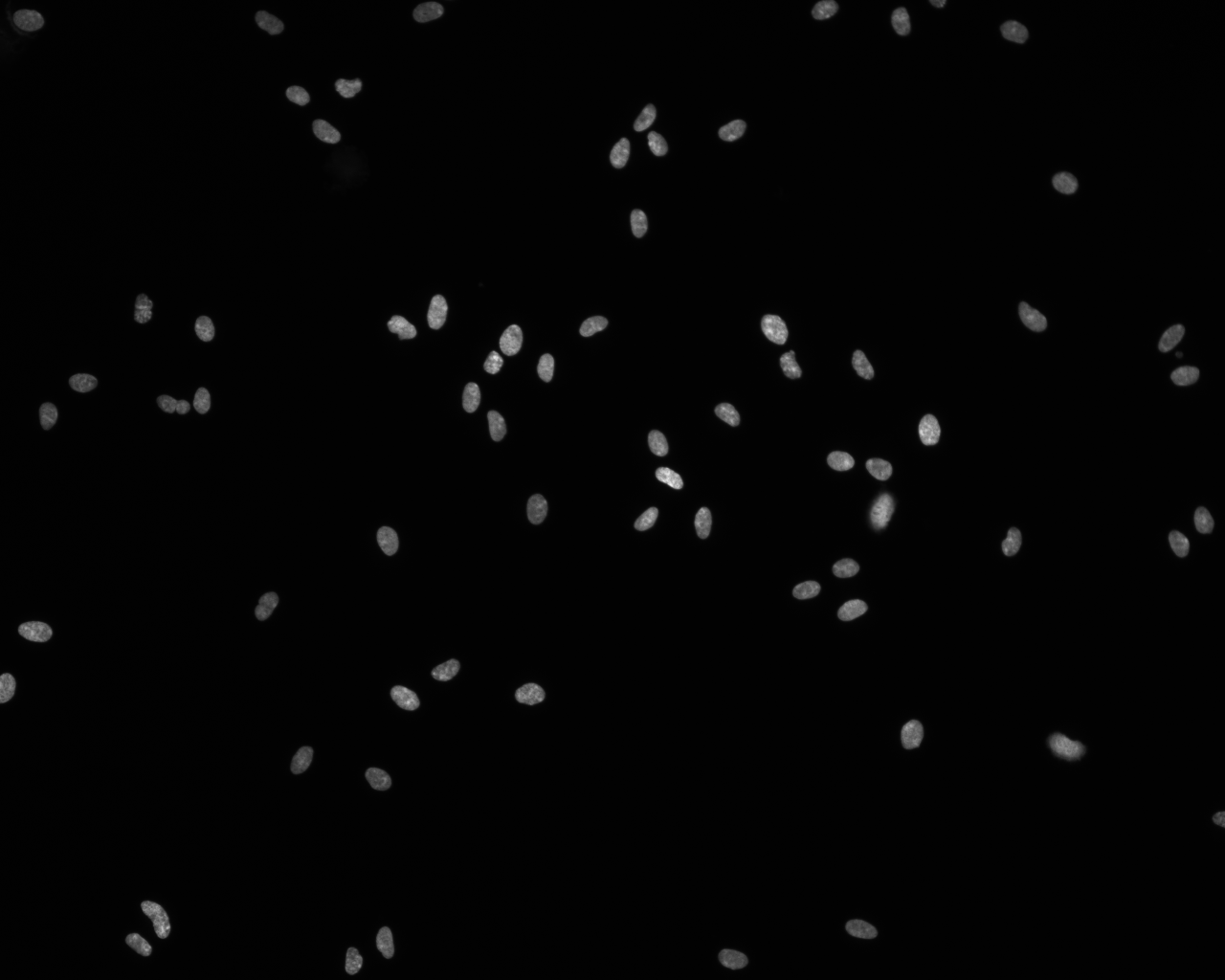

Supplement: S1 Data — (ZIP) [file pone.0233647.s004.zip › rSC48/rSC48_SOX10AF488_EdUAF555_VIMEDL650_DAPI_20x_3_DAPI 1.tif]

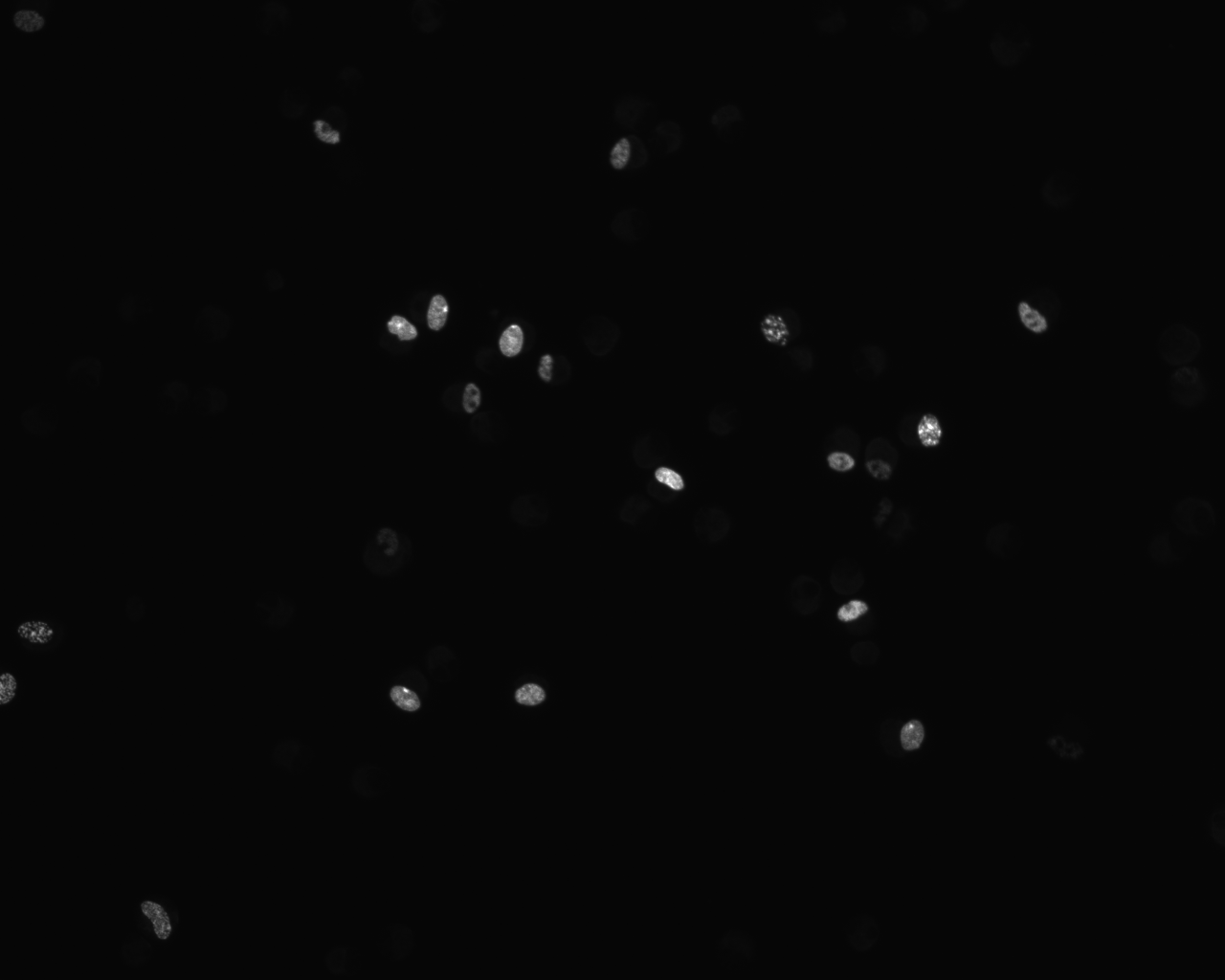

Supplement: S1 Data — (ZIP) [file pone.0233647.s004.zip › rSC48/rSC48_SOX10AF488_EdUAF555_VIMEDL650_DAPI_20x_3_EdU 4.tif]

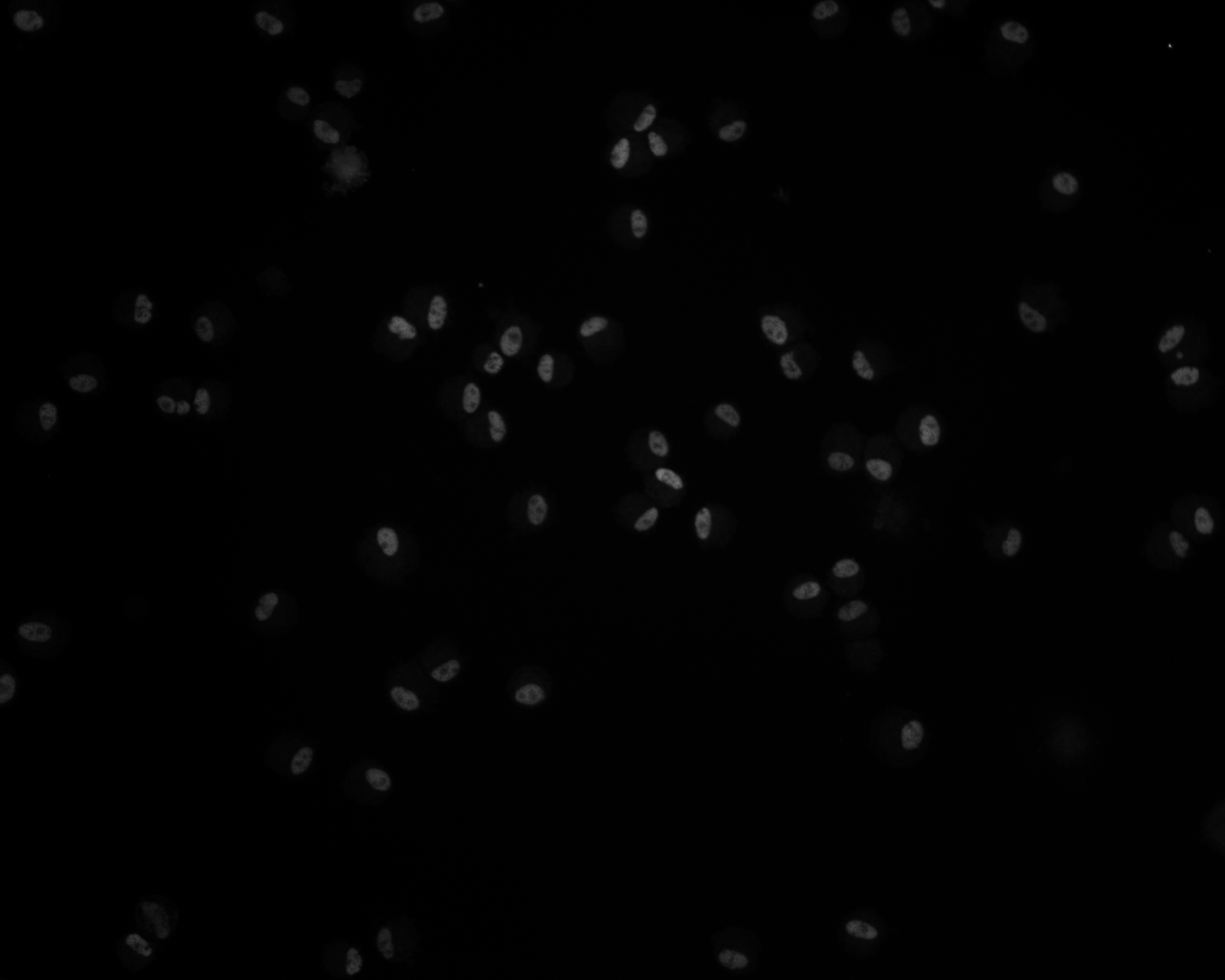

Supplement: S1 Data — (ZIP) [file pone.0233647.s004.zip › rSC48/rSC48_SOX10AF488_EdUAF555_VIMEDL650_DAPI_20x_3_SOX10 2.tif]

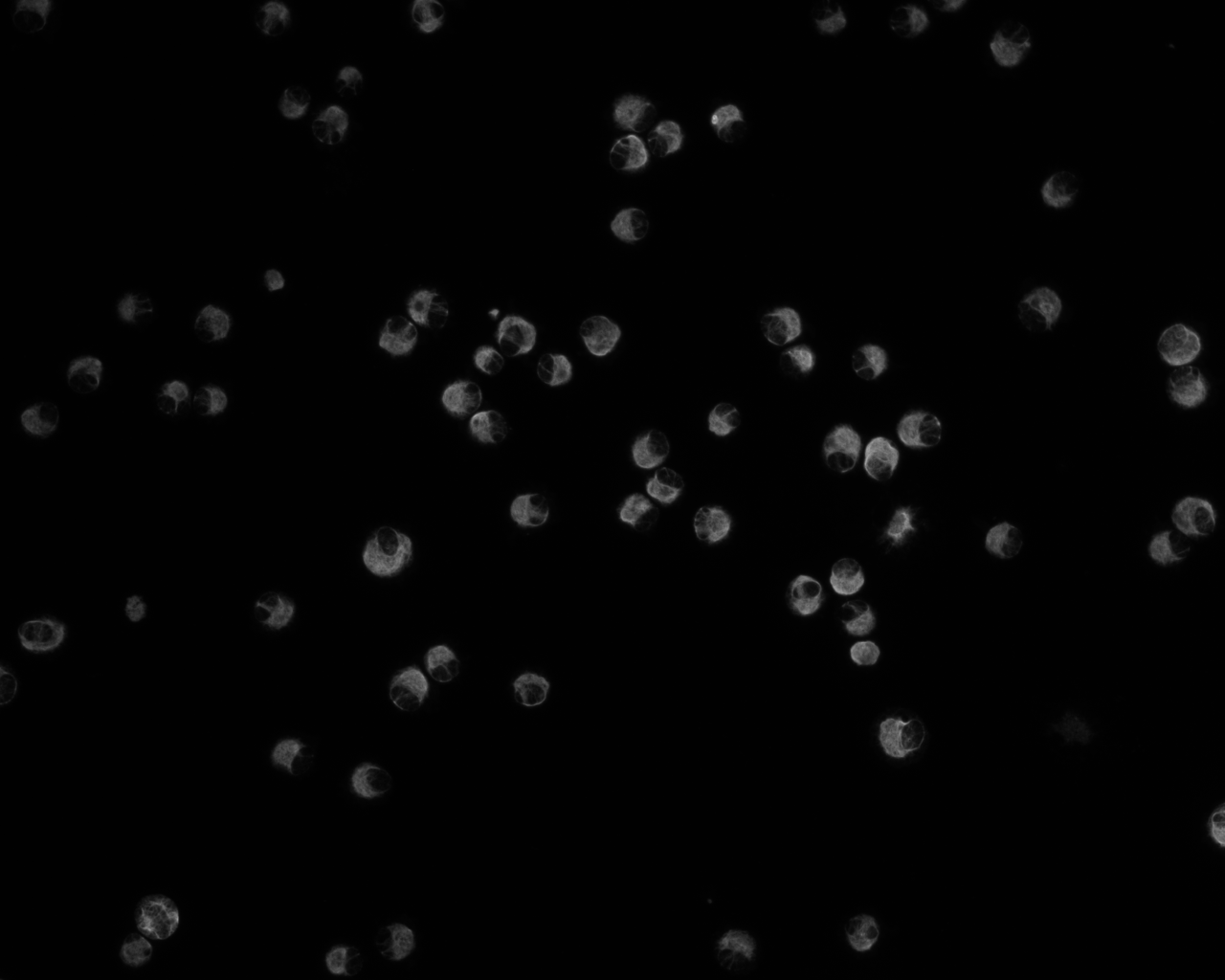

Supplement: S1 Data — (ZIP) [file pone.0233647.s004.zip › rSC48/rSC48_SOX10AF488_EdUAF555_VIMEDL650_DAPI_20x_3_VIME 3.tif]

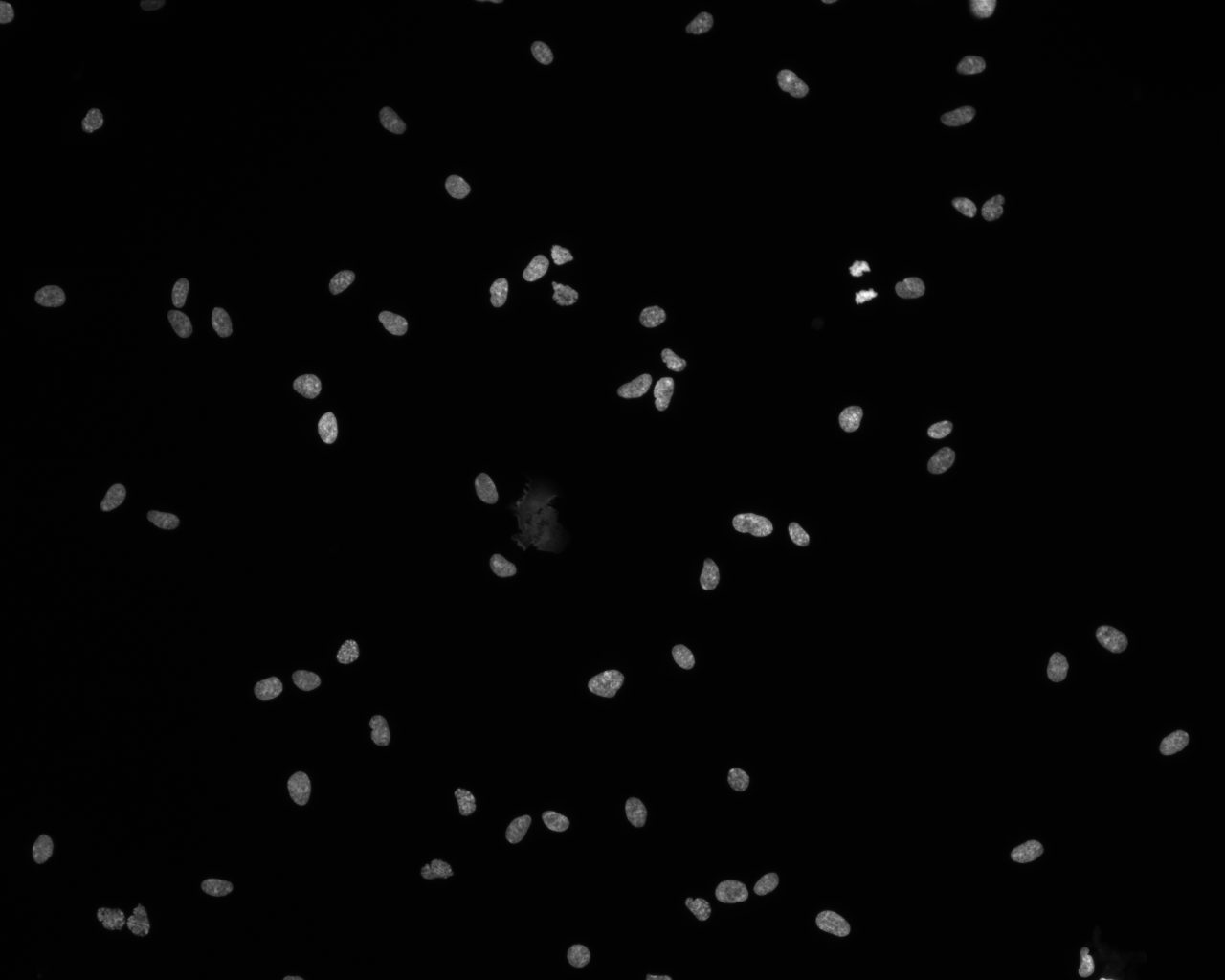

Supplement: S1 Data — (ZIP) [file pone.0233647.s004.zip › rSC48/rSC48_SOX10AF488_EdUAF555_VIMEDL650_DAPI_20x_4_DAPI 1.tif]

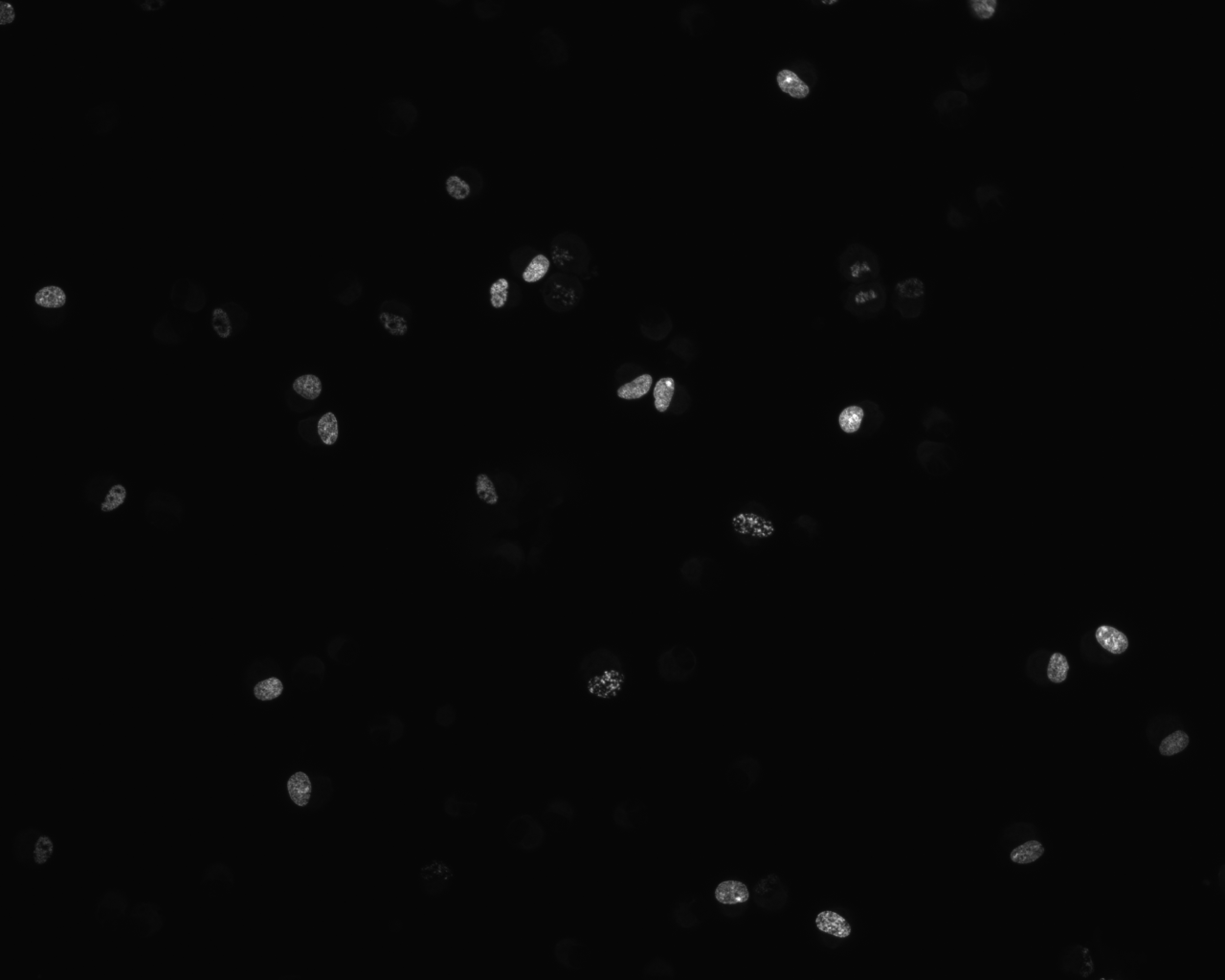

Supplement: S1 Data — (ZIP) [file pone.0233647.s004.zip › rSC48/rSC48_SOX10AF488_EdUAF555_VIMEDL650_DAPI_20x_4_EdU 4.tif]

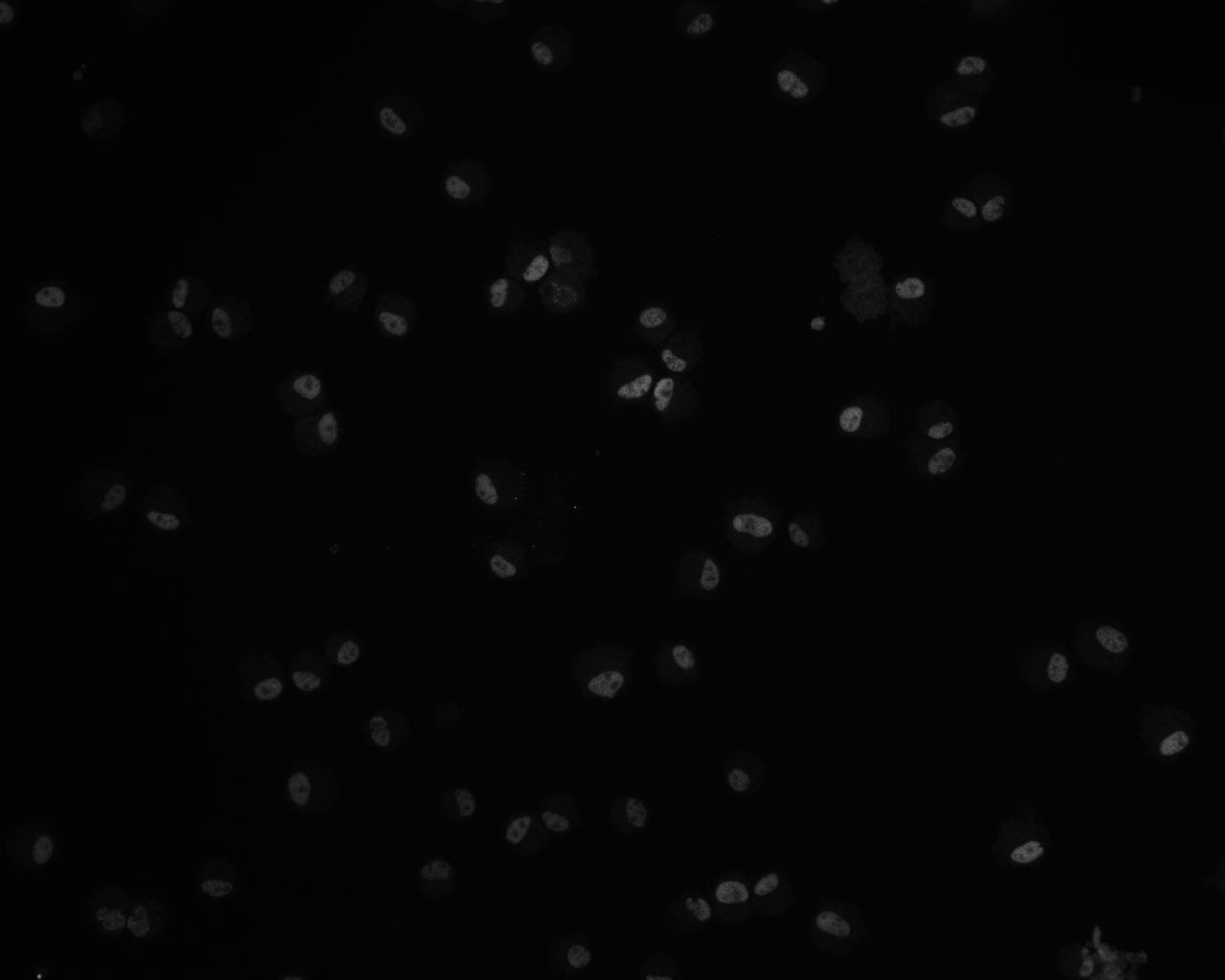

Supplement: S1 Data — (ZIP) [file pone.0233647.s004.zip › rSC48/rSC48_SOX10AF488_EdUAF555_VIMEDL650_DAPI_20x_4_SOX10 2.tif]

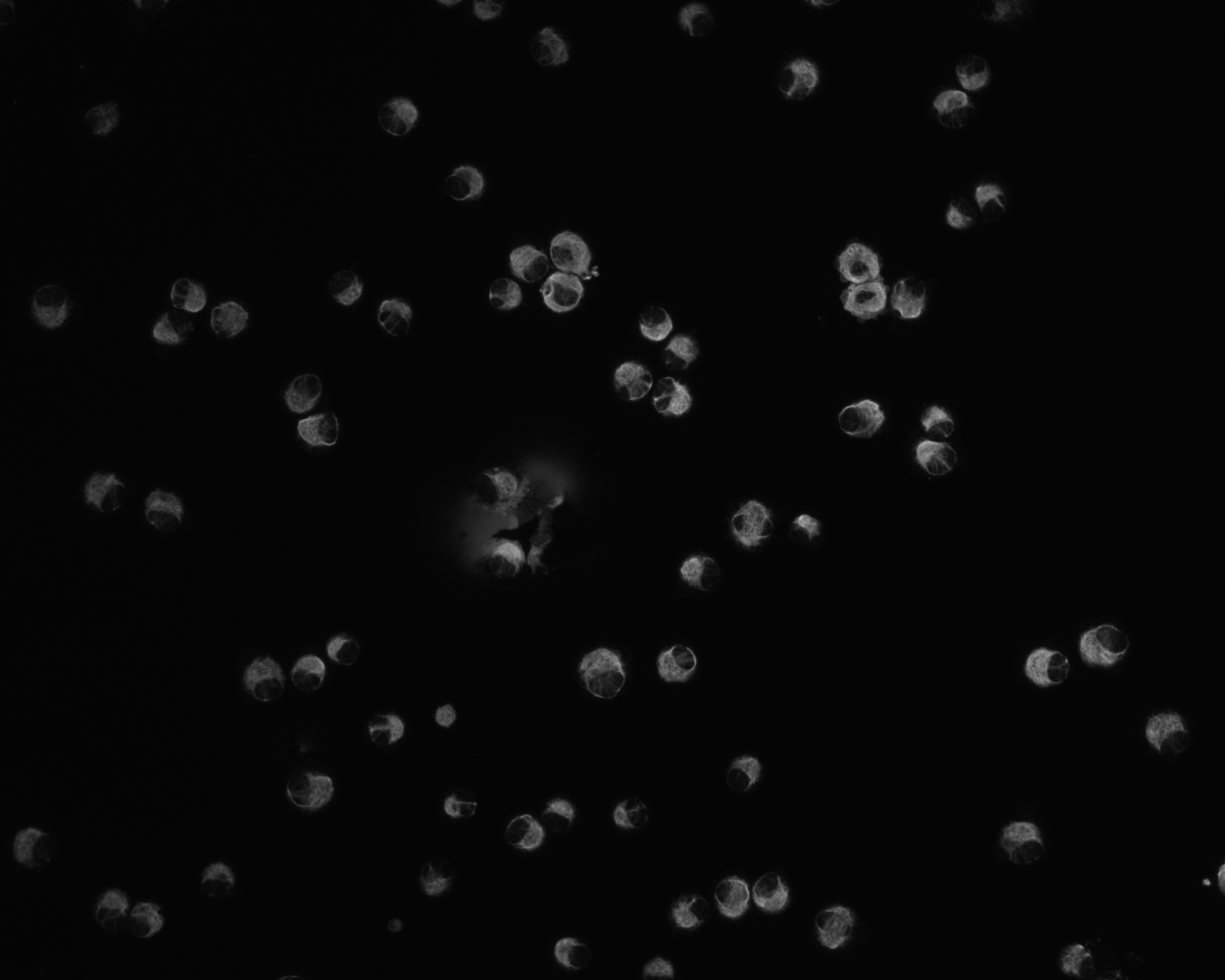

Supplement: S1 Data — (ZIP) [file pone.0233647.s004.zip › rSC48/rSC48_SOX10AF488_EdUAF555_VIMEDL650_DAPI_20x_4_VIME 3.tif]

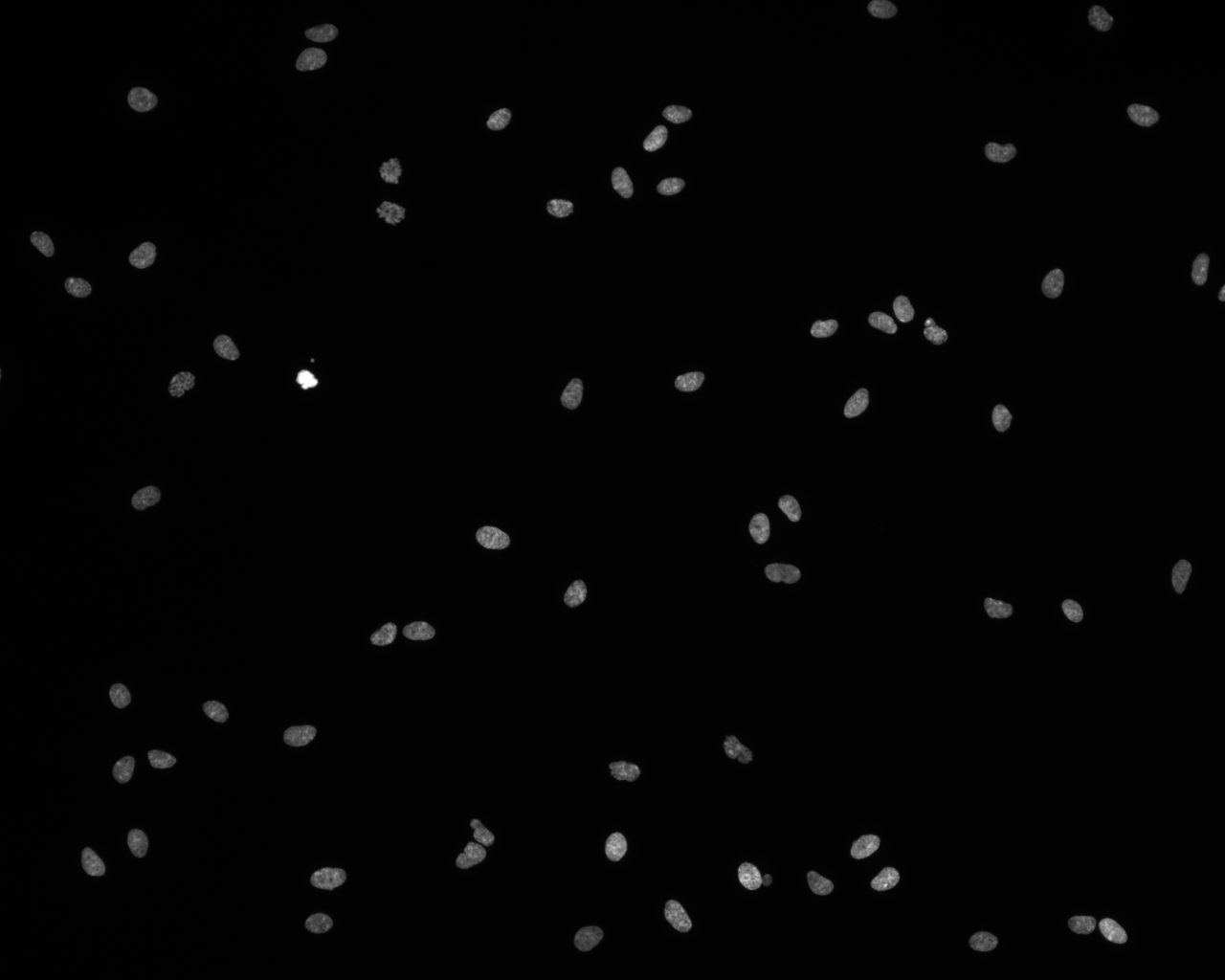

Supplement: S1 Data — (ZIP) [file pone.0233647.s004.zip › rSC48/rSC48_SOX10AF488_EdUAF555_VIMEDL650_DAPI_20x_5_DAPI 1.tif]

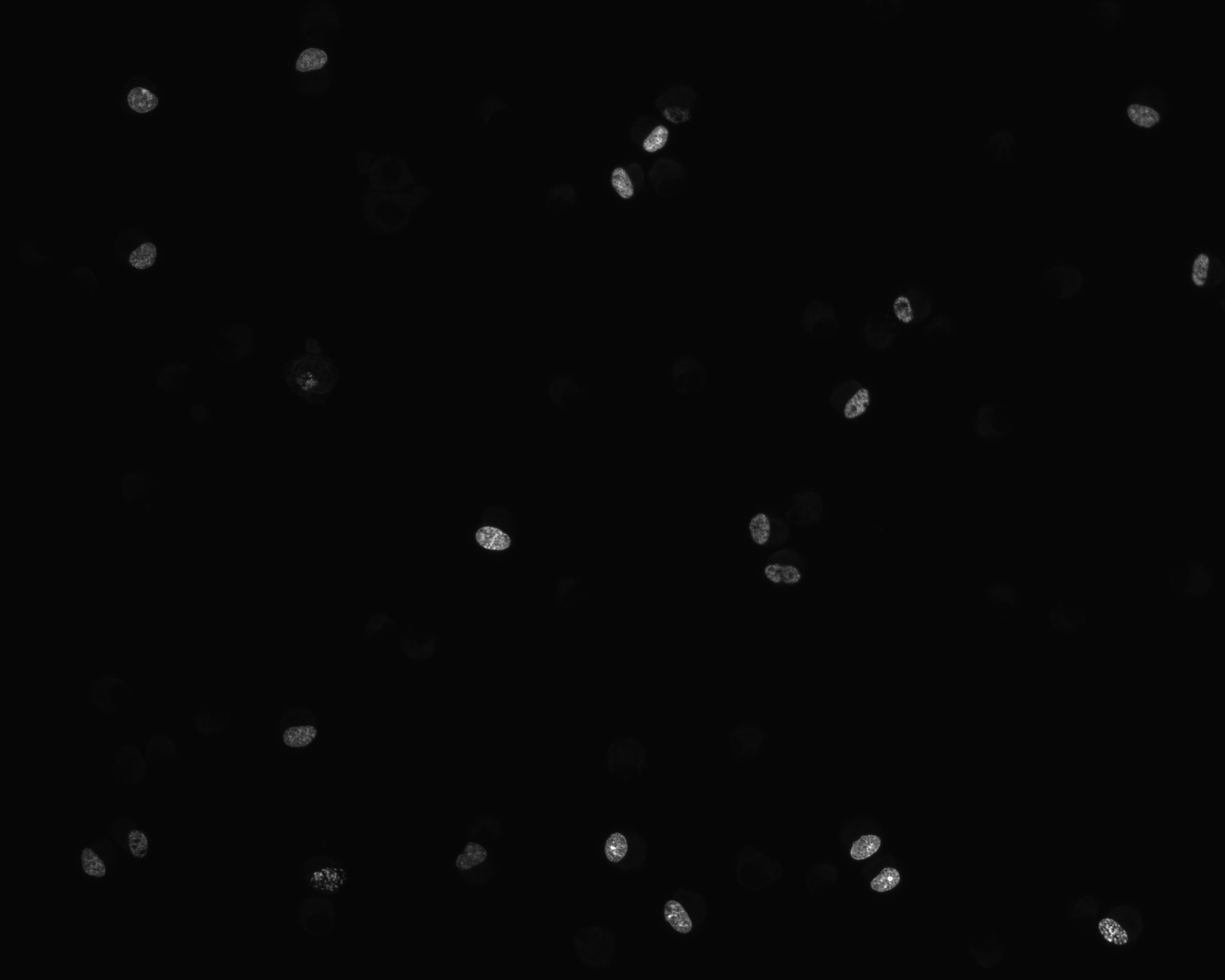

Supplement: S1 Data — (ZIP) [file pone.0233647.s004.zip › rSC48/rSC48_SOX10AF488_EdUAF555_VIMEDL650_DAPI_20x_5_EdU 4.tif]

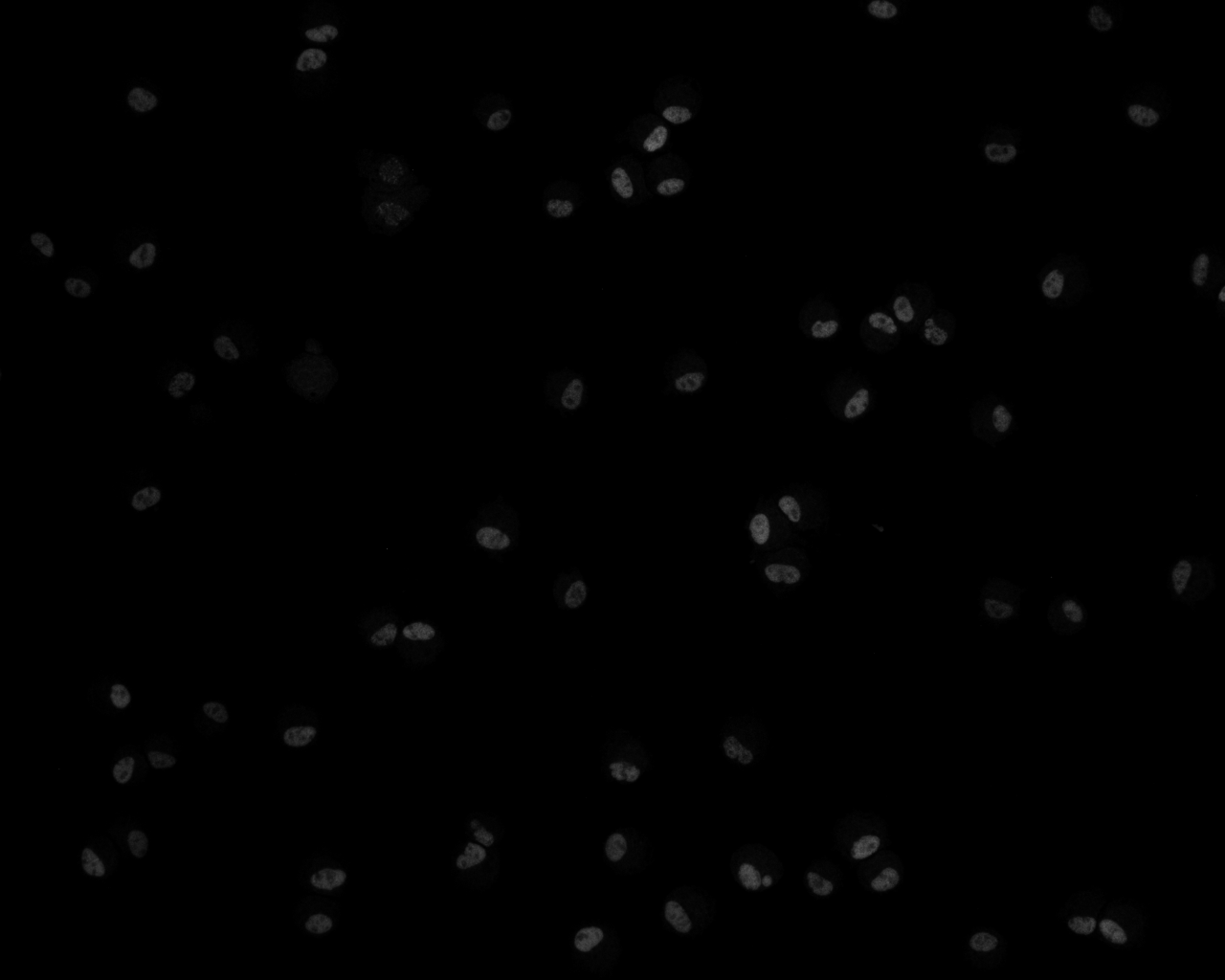

Supplement: S1 Data — (ZIP) [file pone.0233647.s004.zip › rSC48/rSC48_SOX10AF488_EdUAF555_VIMEDL650_DAPI_20x_5_SOX10 2.tif]

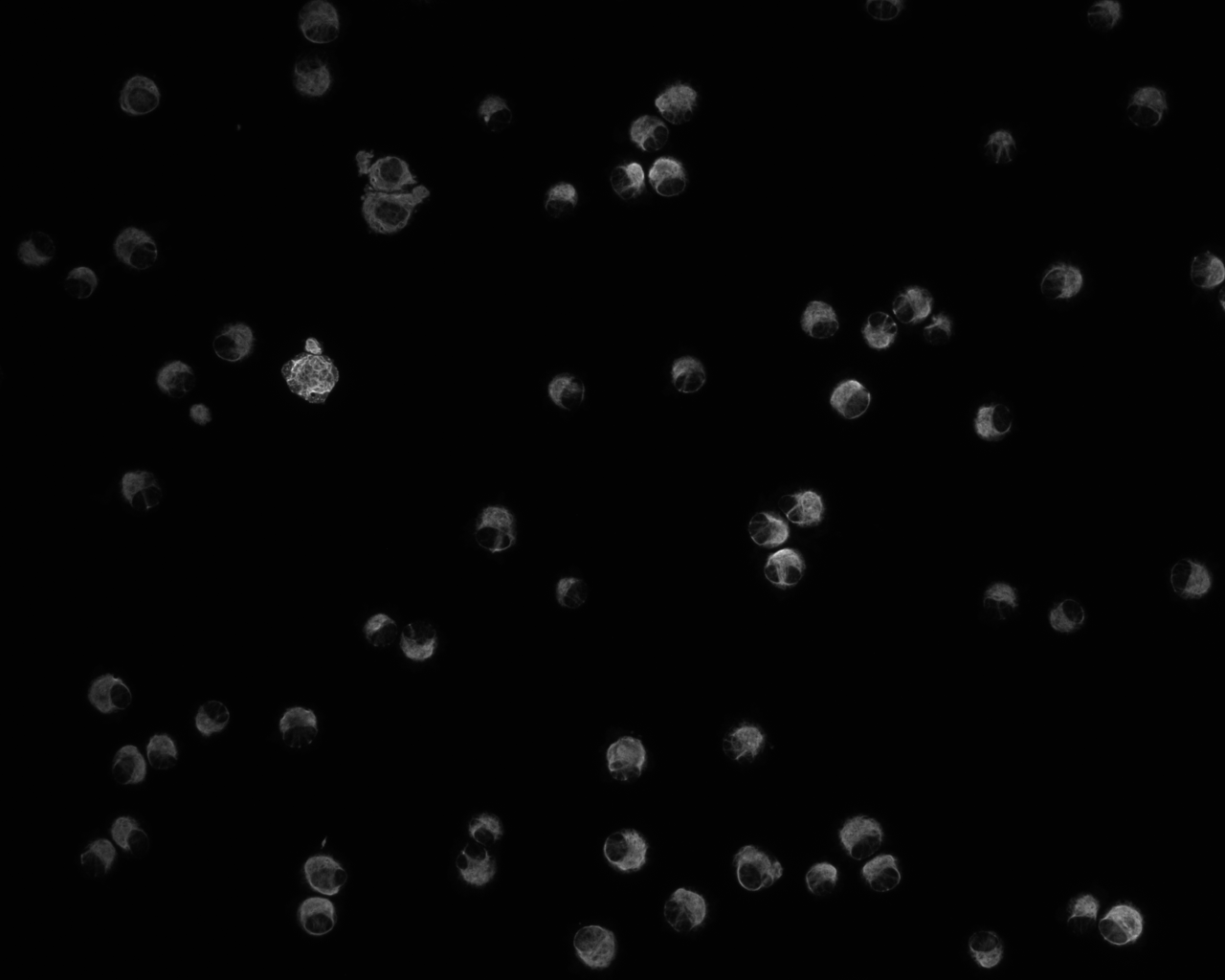

Supplement: S1 Data — (ZIP) [file pone.0233647.s004.zip › rSC48/rSC48_SOX10AF488_EdUAF555_VIMEDL650_DAPI_20x_5_VIME 3.tif]

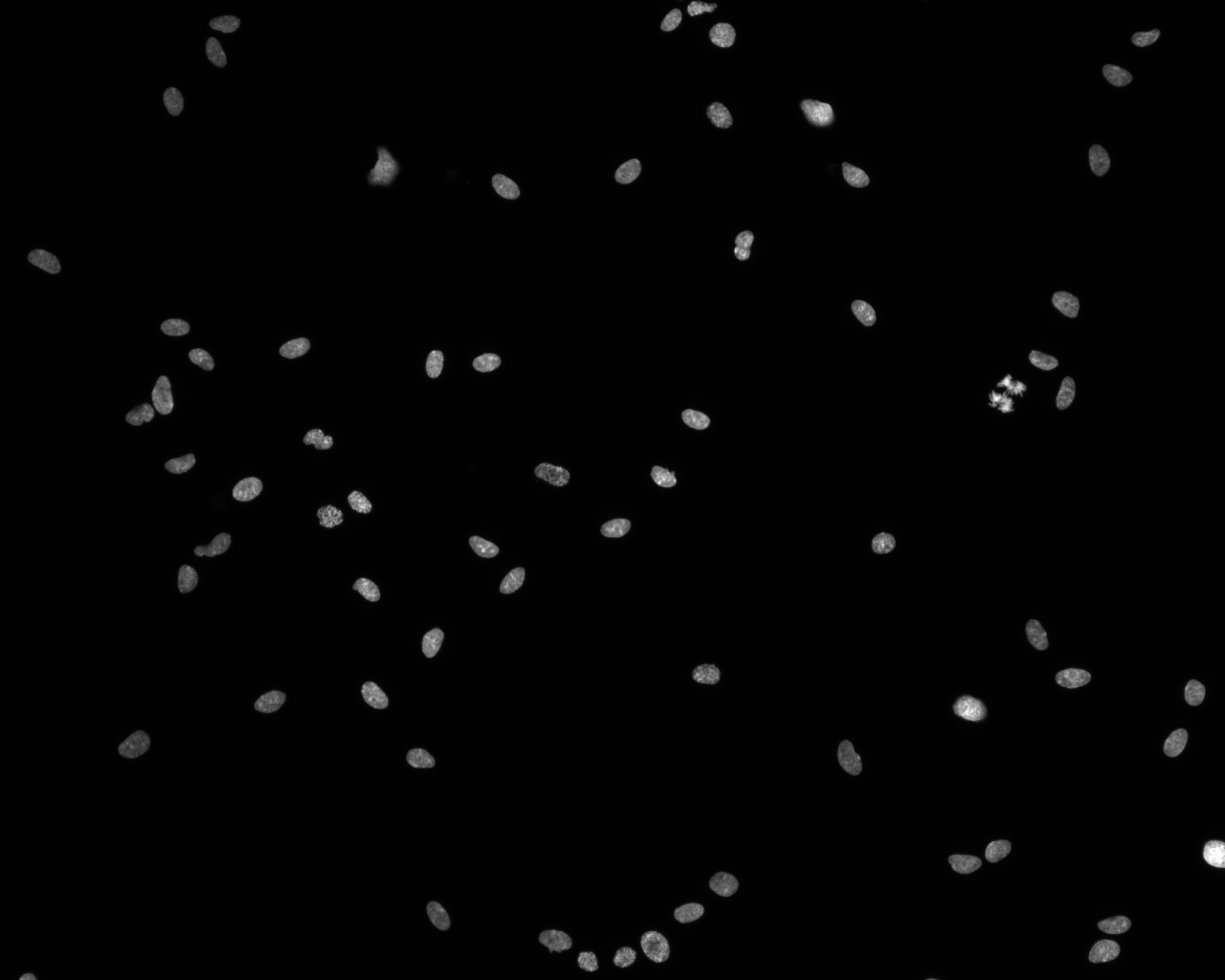

Supplement: S1 Data — (ZIP) [file pone.0233647.s004.zip › rSC48/rSC48_SOX10AF488_EdUAF555_VIMEDL650_DAPI_20x_6_DAPI 1.tif]

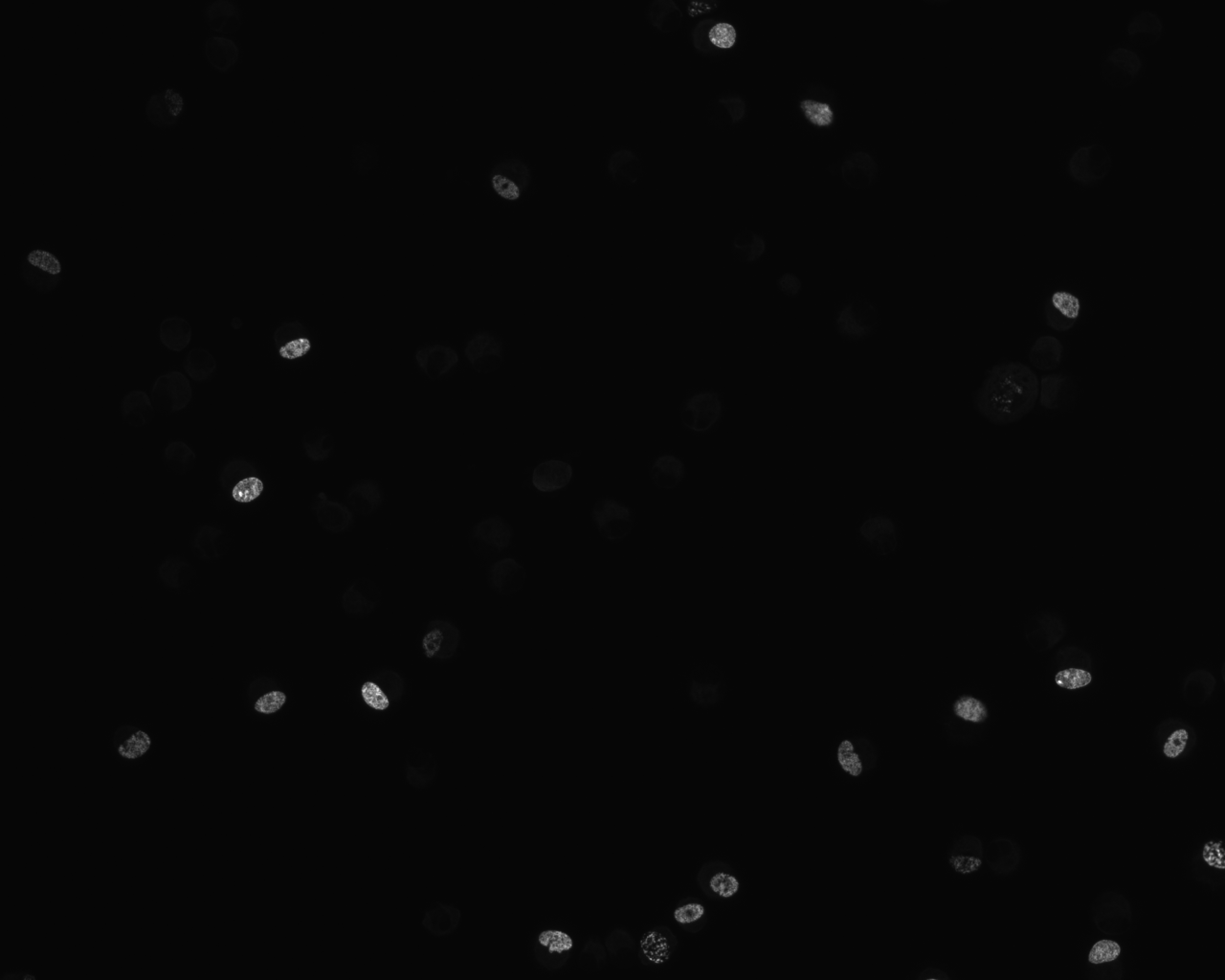

Supplement: S1 Data — (ZIP) [file pone.0233647.s004.zip › rSC48/rSC48_SOX10AF488_EdUAF555_VIMEDL650_DAPI_20x_6_EdU 4.tif]

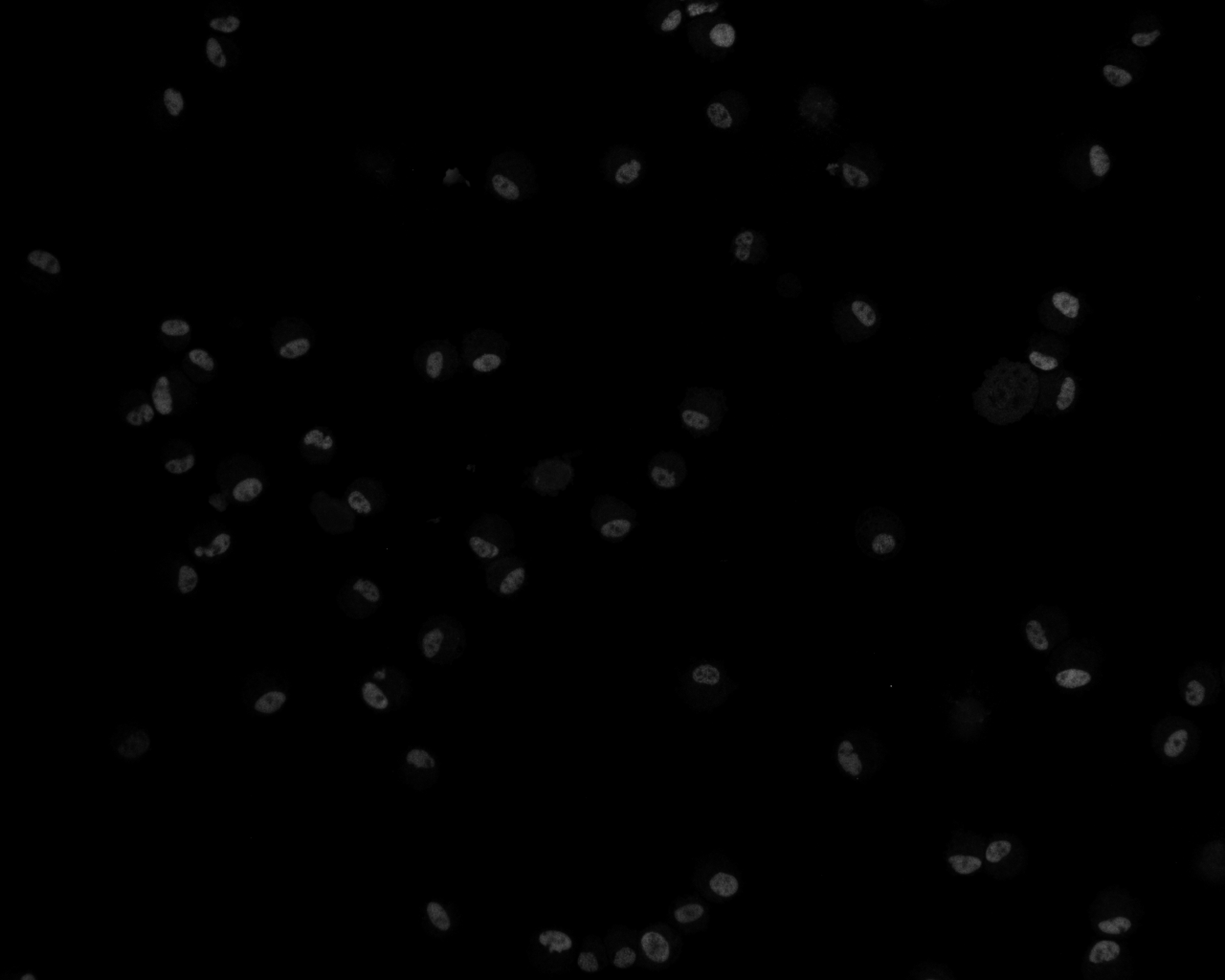

Supplement: S1 Data — (ZIP) [file pone.0233647.s004.zip › rSC48/rSC48_SOX10AF488_EdUAF555_VIMEDL650_DAPI_20x_6_SOX10 2.tif]

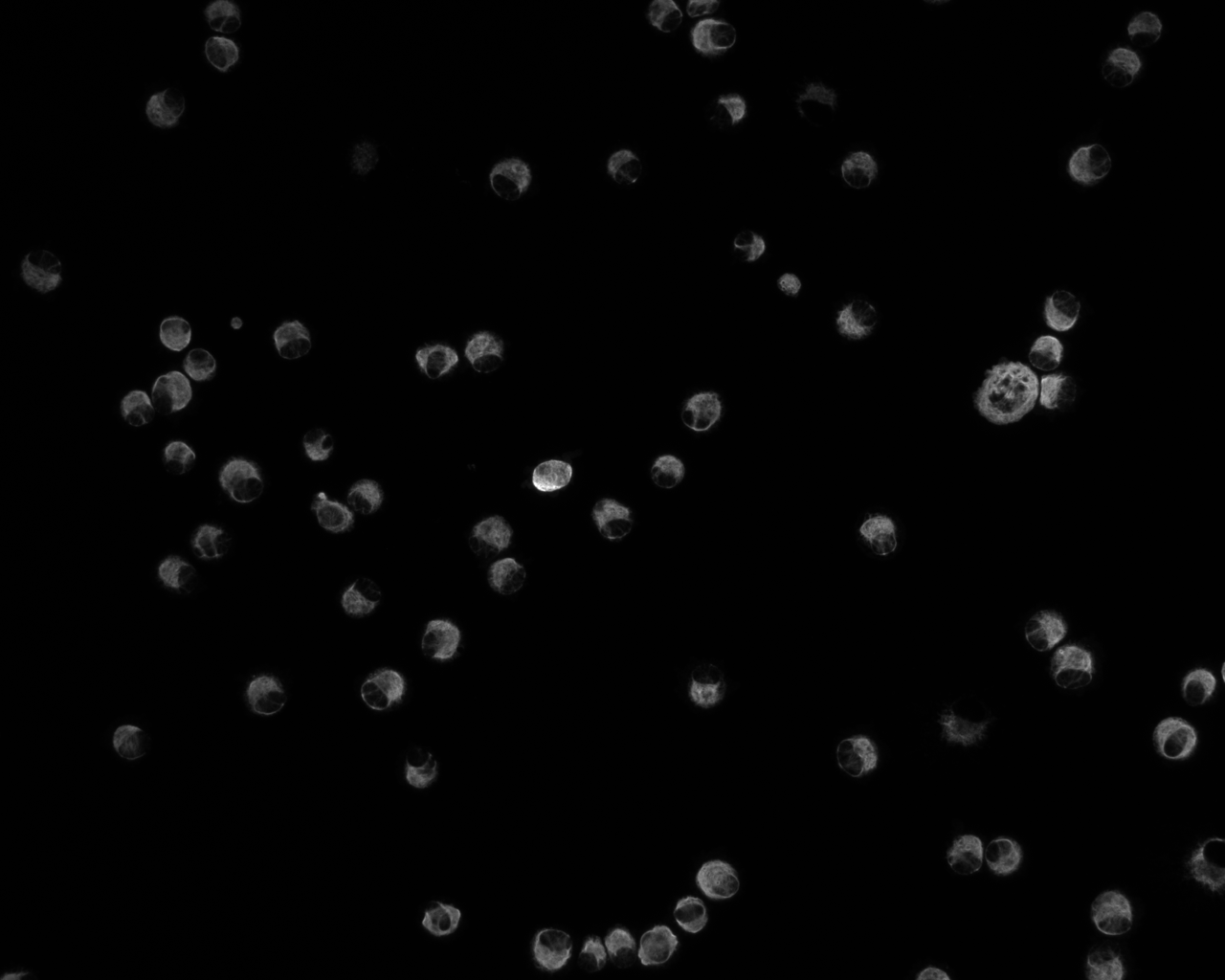

Supplement: S1 Data — (ZIP) [file pone.0233647.s004.zip › rSC48/rSC48_SOX10AF488_EdUAF555_VIMEDL650_DAPI_20x_6_VIME 3.tif]

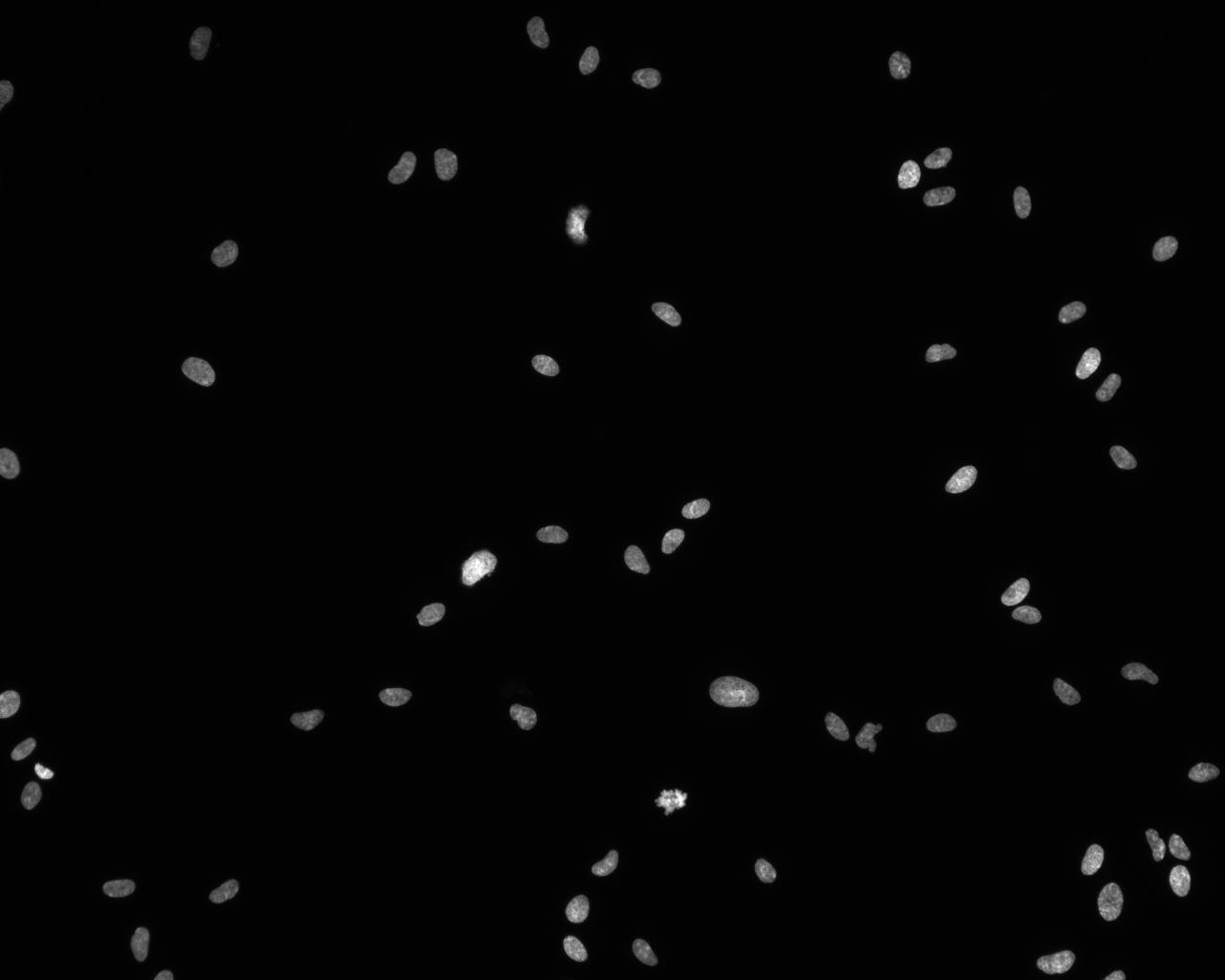

Supplement: S1 Data — (ZIP) [file pone.0233647.s004.zip › rSC48/rSC48_SOX10AF488_EdUAF555_VIMEDL650_DAPI_20x_7_DAPI 1.tif]

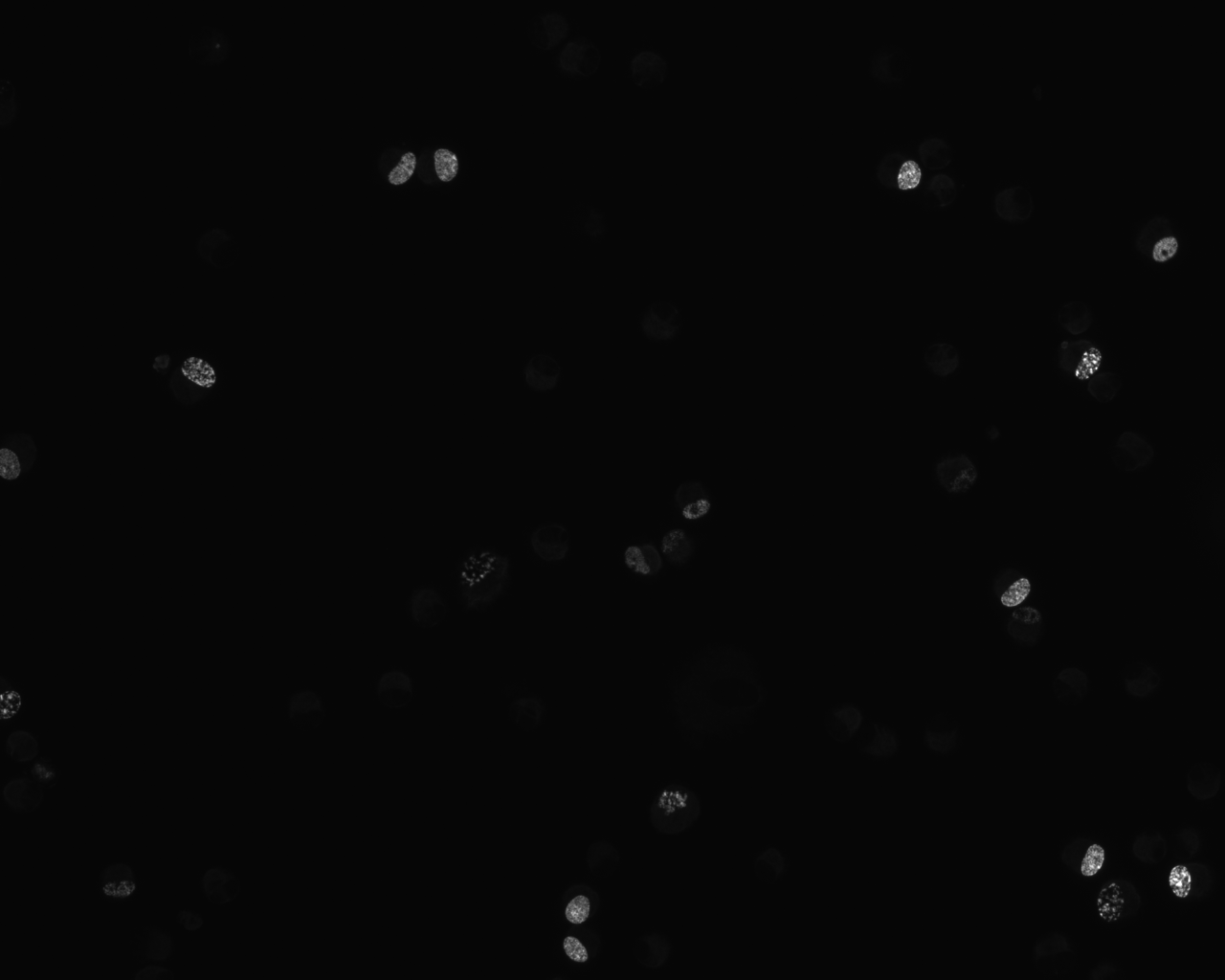

Supplement: S1 Data — (ZIP) [file pone.0233647.s004.zip › rSC48/rSC48_SOX10AF488_EdUAF555_VIMEDL650_DAPI_20x_7_EdU 4.tif]

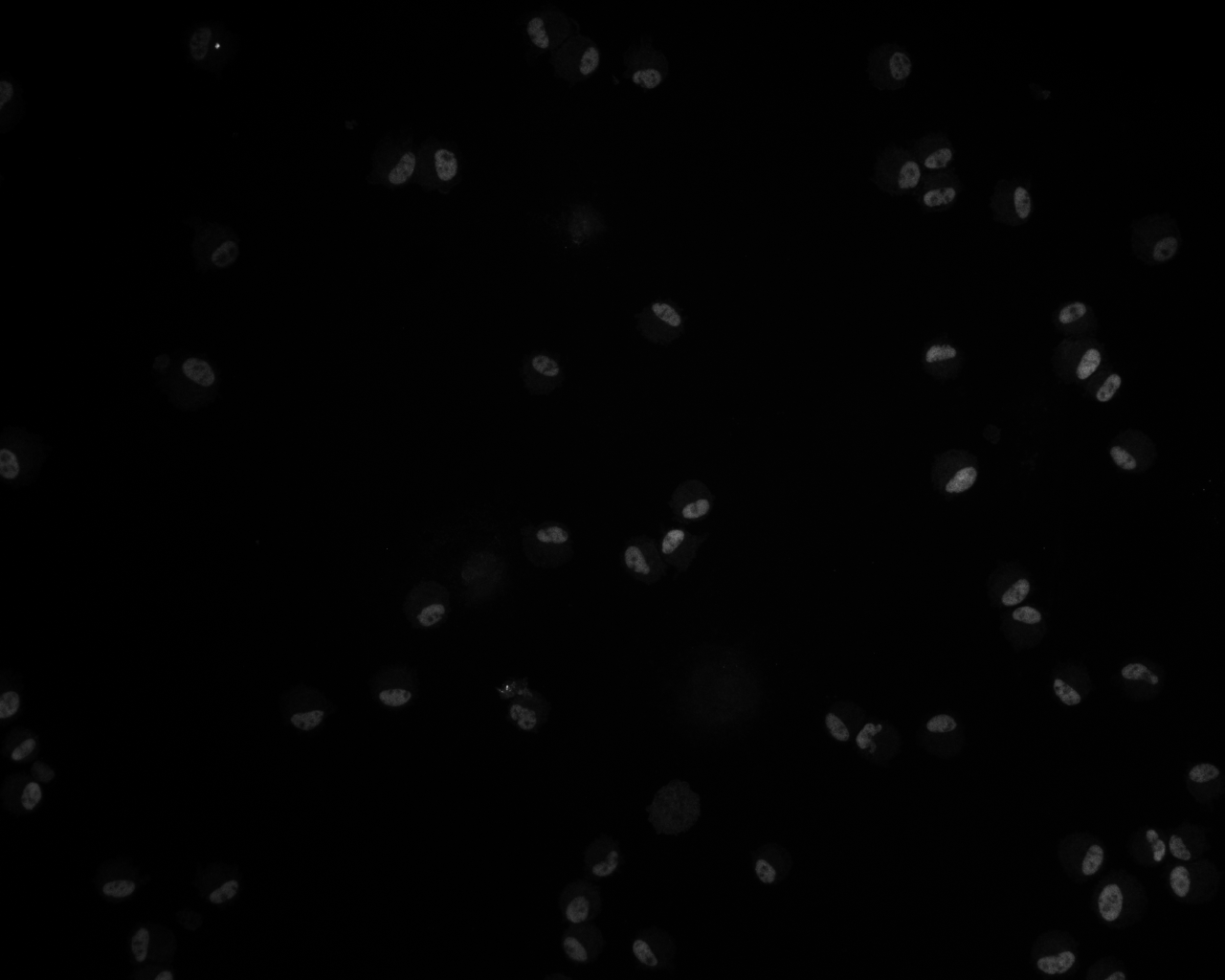

Supplement: S1 Data — (ZIP) [file pone.0233647.s004.zip › rSC48/rSC48_SOX10AF488_EdUAF555_VIMEDL650_DAPI_20x_7_SOX10 2.tif]

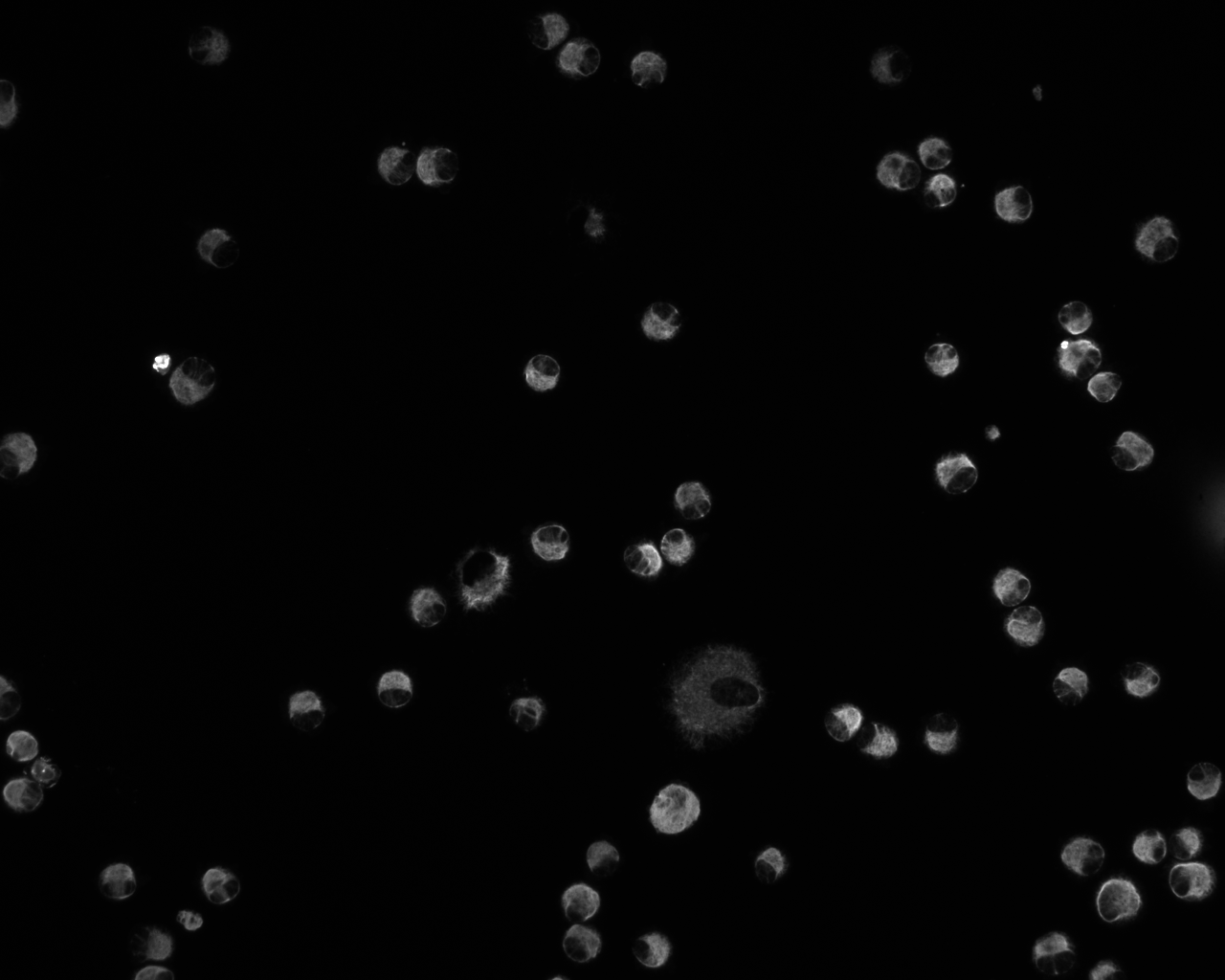

Supplement: S1 Data — (ZIP) [file pone.0233647.s004.zip › rSC48/rSC48_SOX10AF488_EdUAF555_VIMEDL650_DAPI_20x_7_VIME 3.tif]

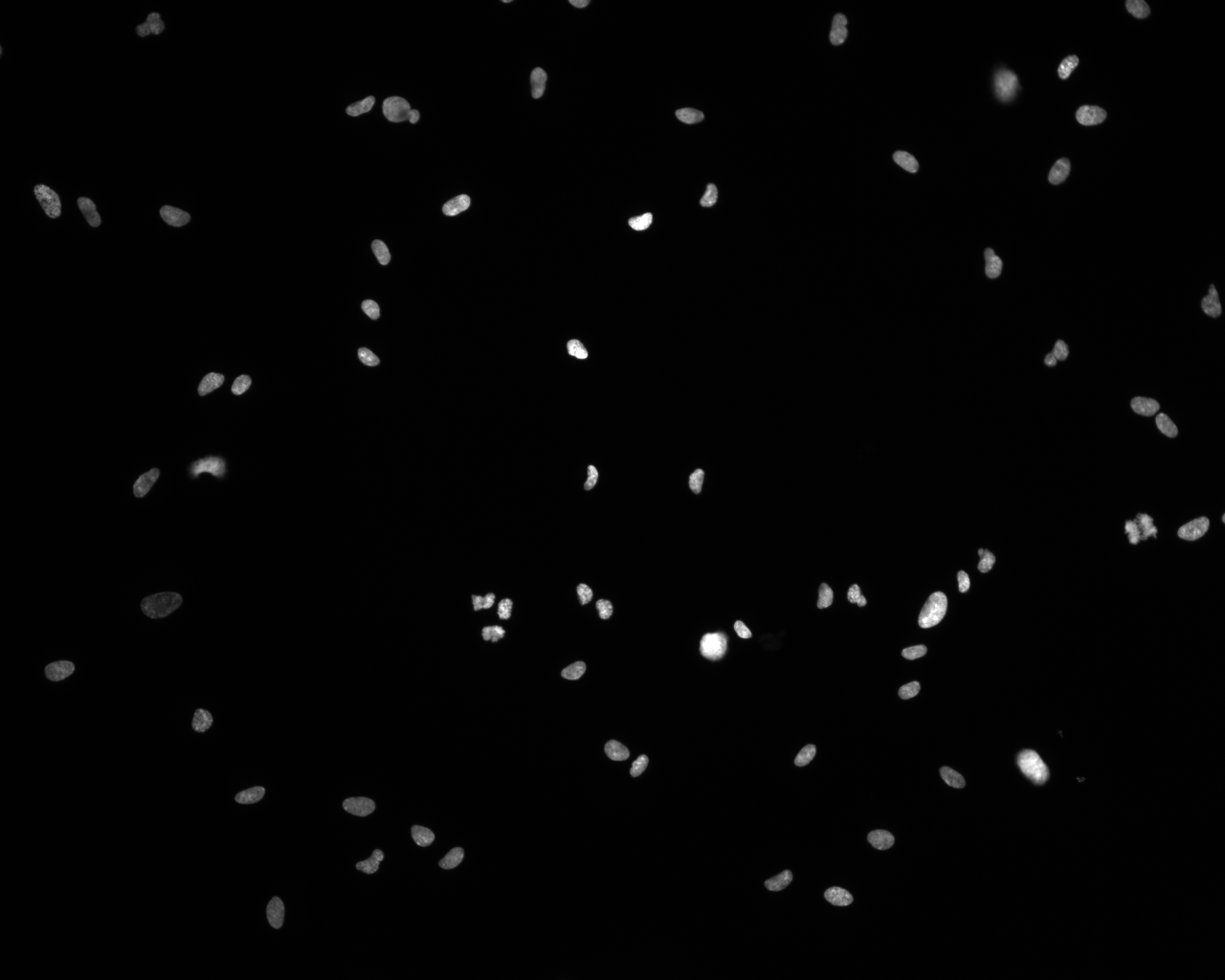

Supplement: S1 Data — (ZIP) [file pone.0233647.s004.zip › rSC48/rSC48_SOX10AF488_EdUAF555_VIMEDL650_DAPI_20x_8_DAPI 1.tif]

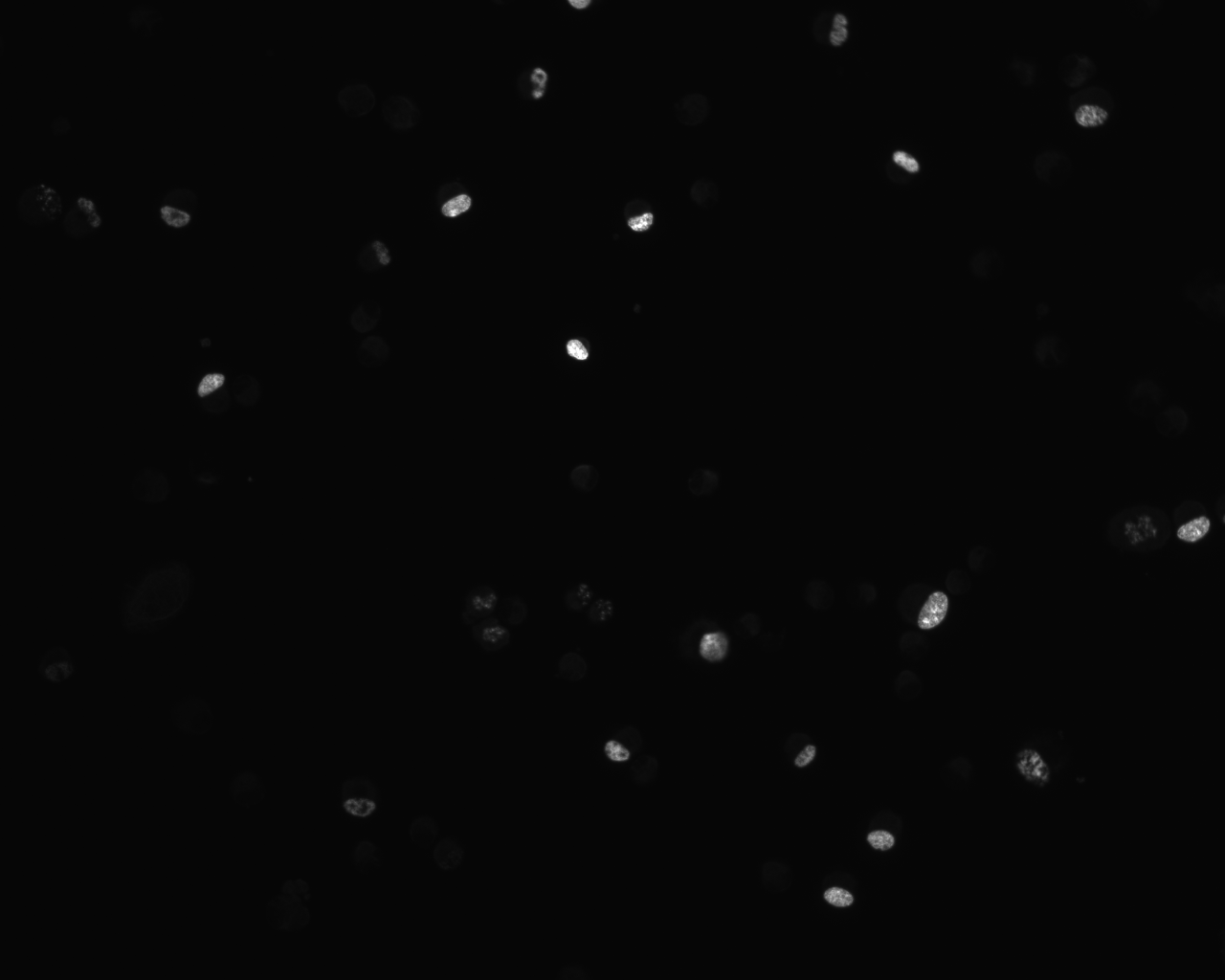

Supplement: S1 Data — (ZIP) [file pone.0233647.s004.zip › rSC48/rSC48_SOX10AF488_EdUAF555_VIMEDL650_DAPI_20x_8_EdU 4.tif]

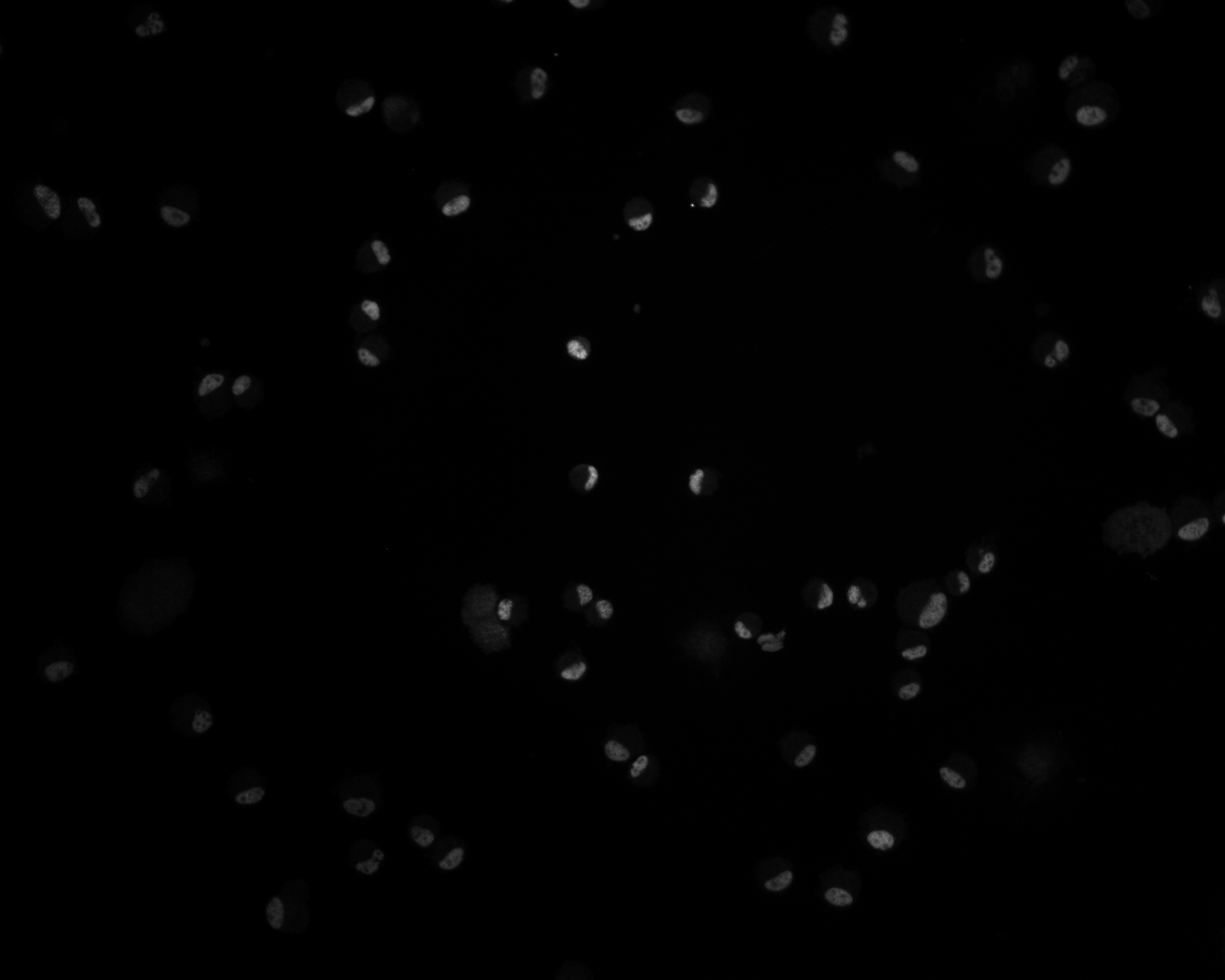

Supplement: S1 Data — (ZIP) [file pone.0233647.s004.zip › rSC48/rSC48_SOX10AF488_EdUAF555_VIMEDL650_DAPI_20x_8_SOX10 2.tif]

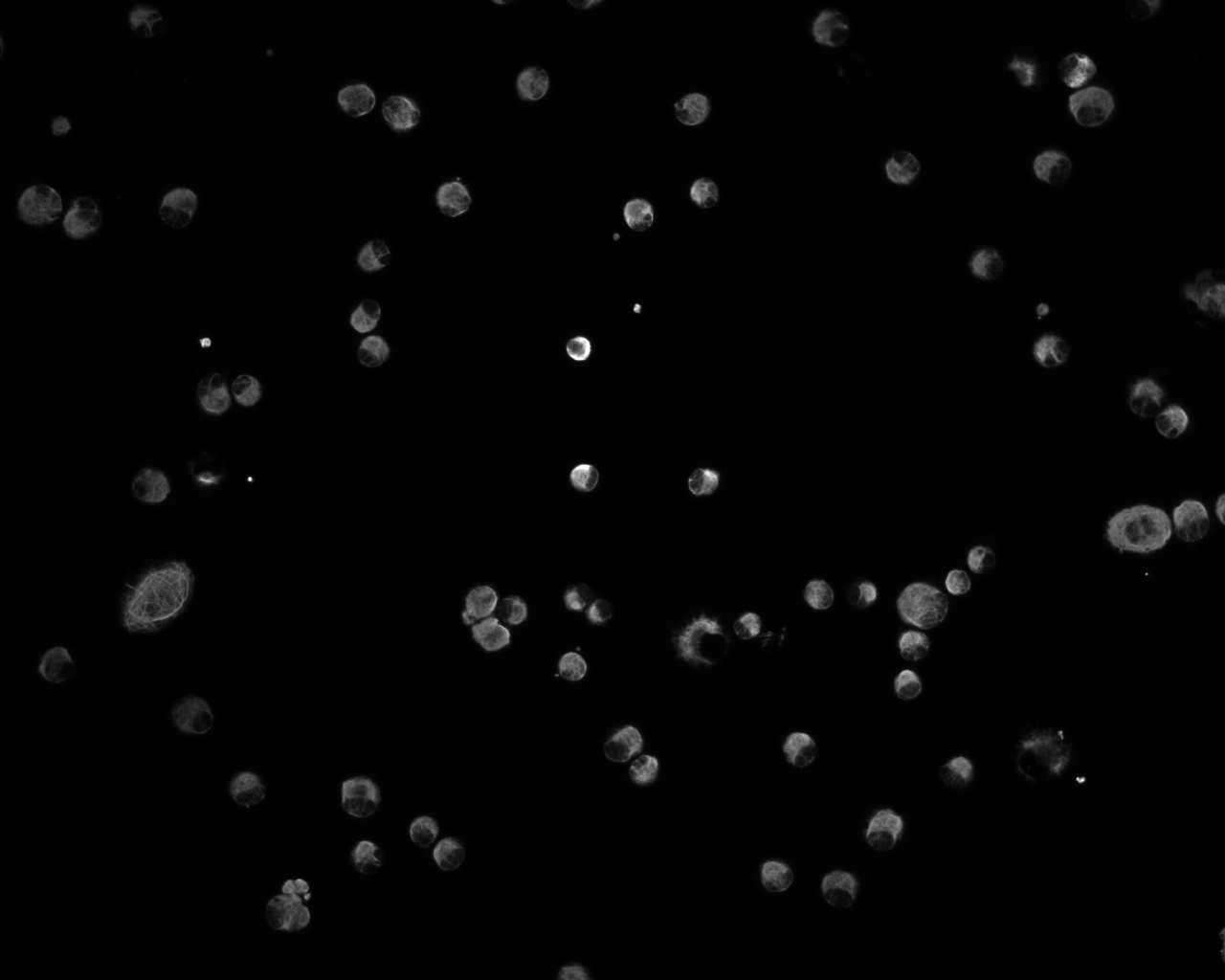

Supplement: S1 Data — (ZIP) [file pone.0233647.s004.zip › rSC48/rSC48_SOX10AF488_EdUAF555_VIMEDL650_DAPI_20x_8_VIME 3.tif]

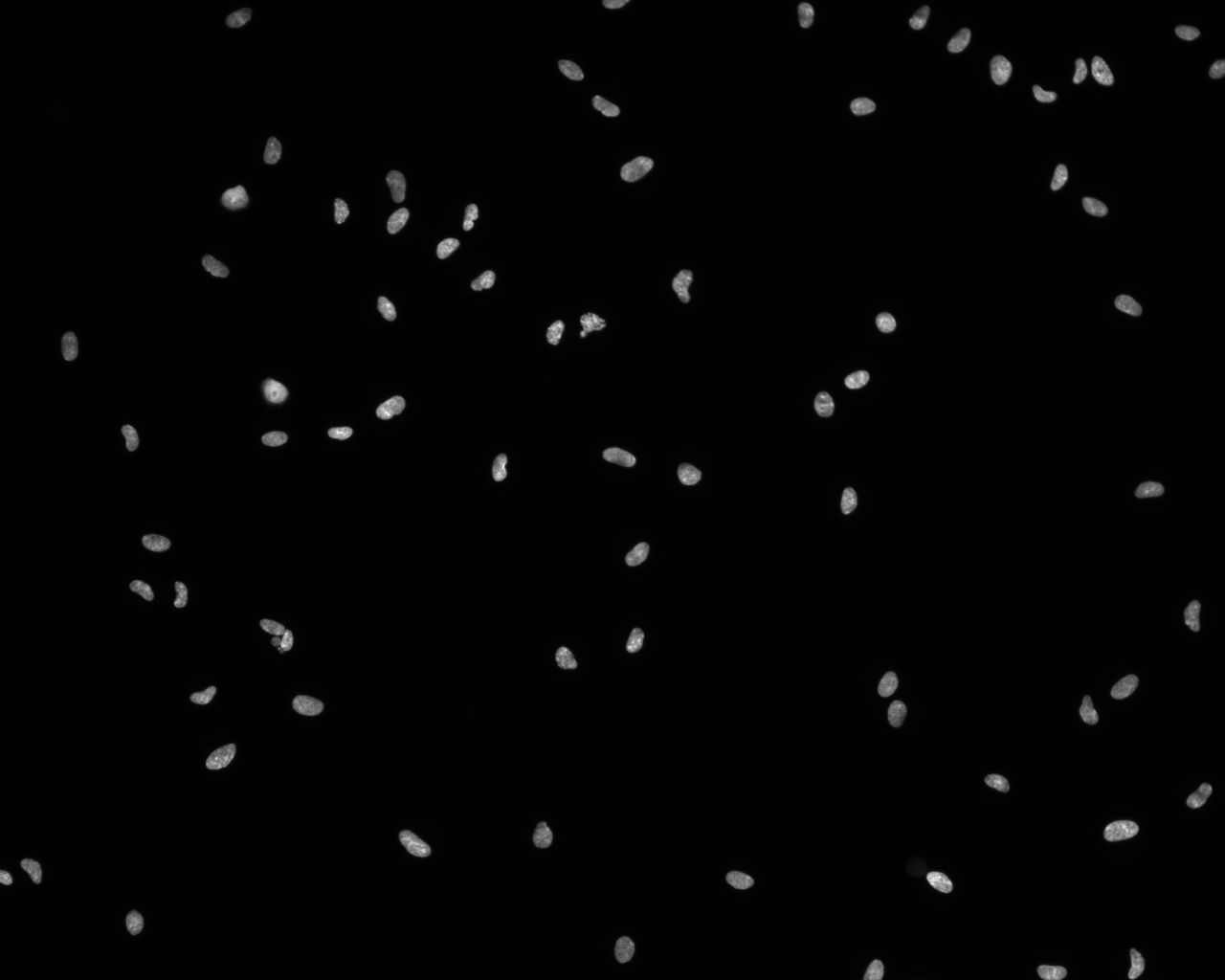

Supplement: S1 Data — (ZIP) [file pone.0233647.s004.zip › rSC48/rSC48_SOX10AF488_EdUAF555_VIMEDL650_DAPI_20x_9_DAPI 1.tif]

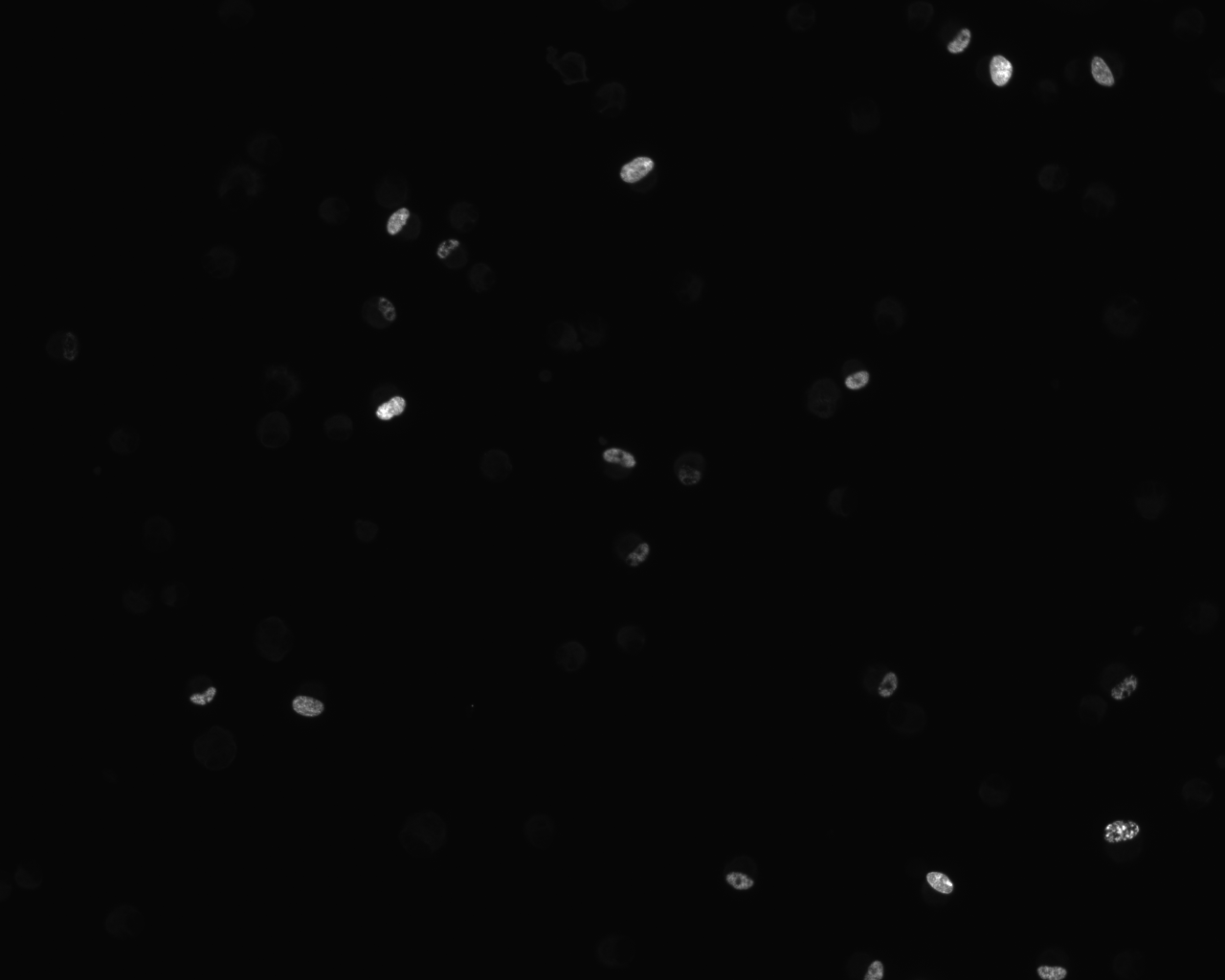

Supplement: S1 Data — (ZIP) [file pone.0233647.s004.zip › rSC48/rSC48_SOX10AF488_EdUAF555_VIMEDL650_DAPI_20x_9_EdU 4.tif]

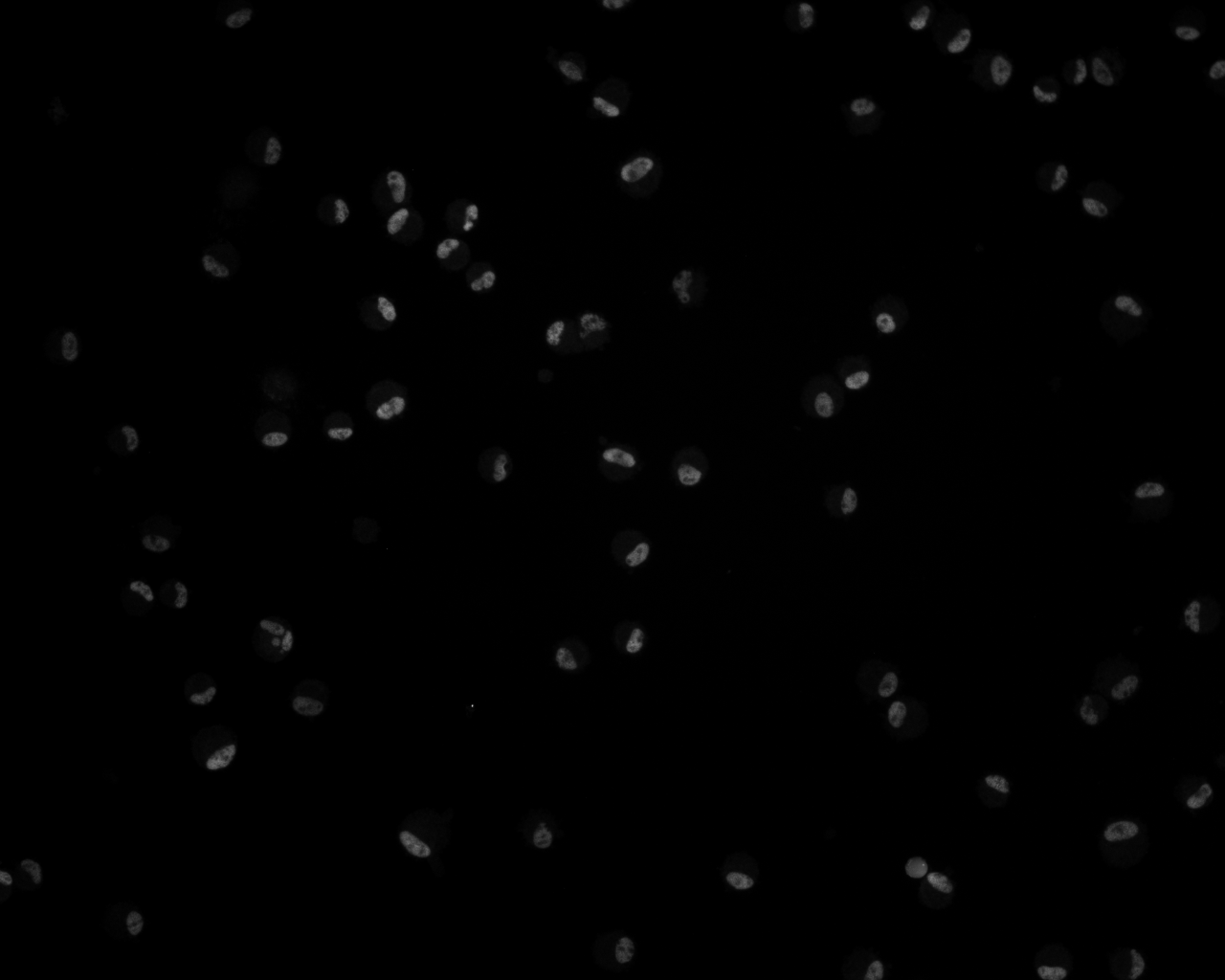

Supplement: S1 Data — (ZIP) [file pone.0233647.s004.zip › rSC48/rSC48_SOX10AF488_EdUAF555_VIMEDL650_DAPI_20x_9_SOX10 2.tif]

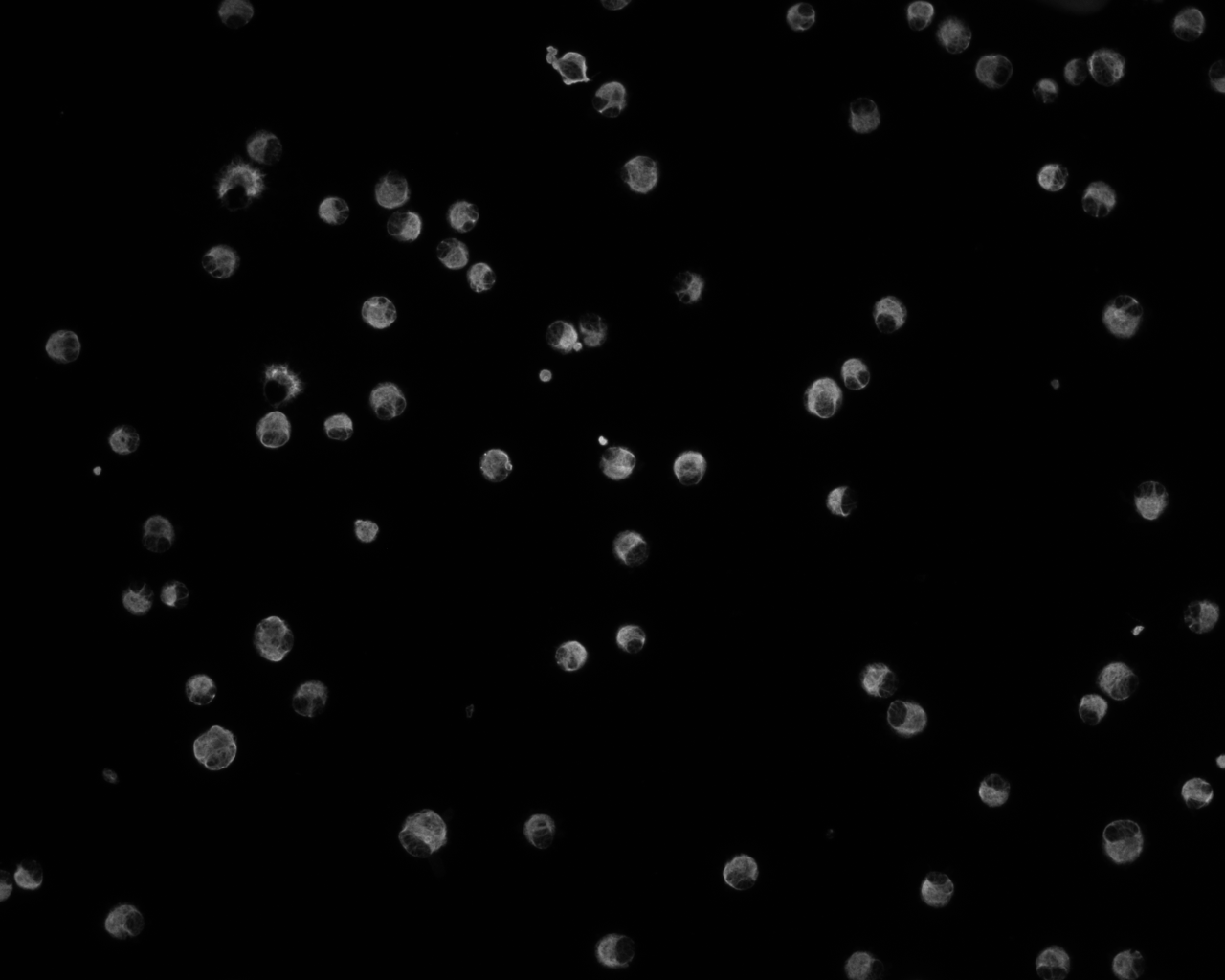

Supplement: S1 Data — (ZIP) [file pone.0233647.s004.zip › rSC48/rSC48_SOX10AF488_EdUAF555_VIMEDL650_DAPI_20x_9_VIME 3.tif]

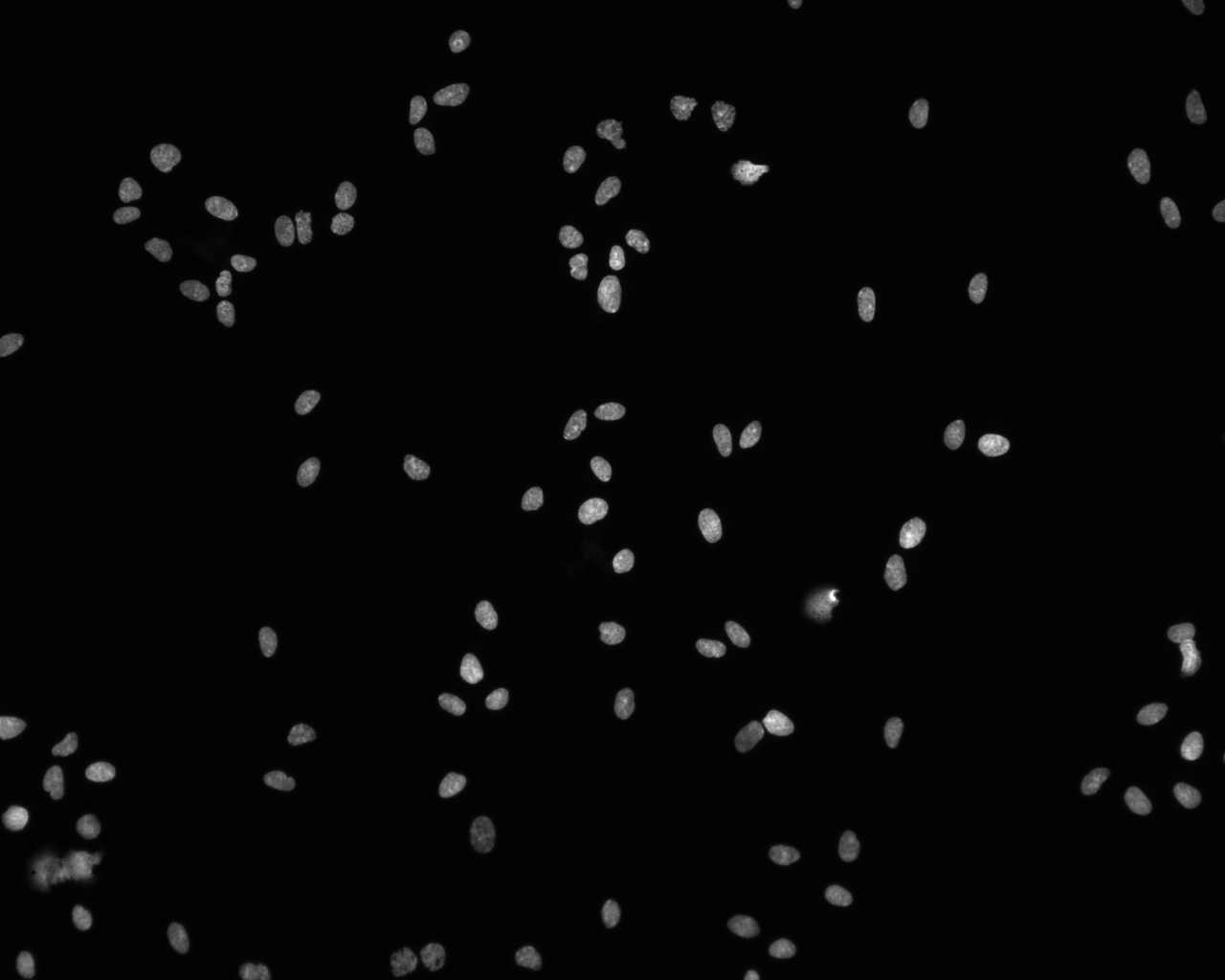

Supplement: S2 Data — (ZIP) [file pone.0233647.s005.zip › PACE Corrected/rSC49_SOX10AF488_EdUAF555_VIMEDL650_DAPI_20x_1_DAPI 1.tif]

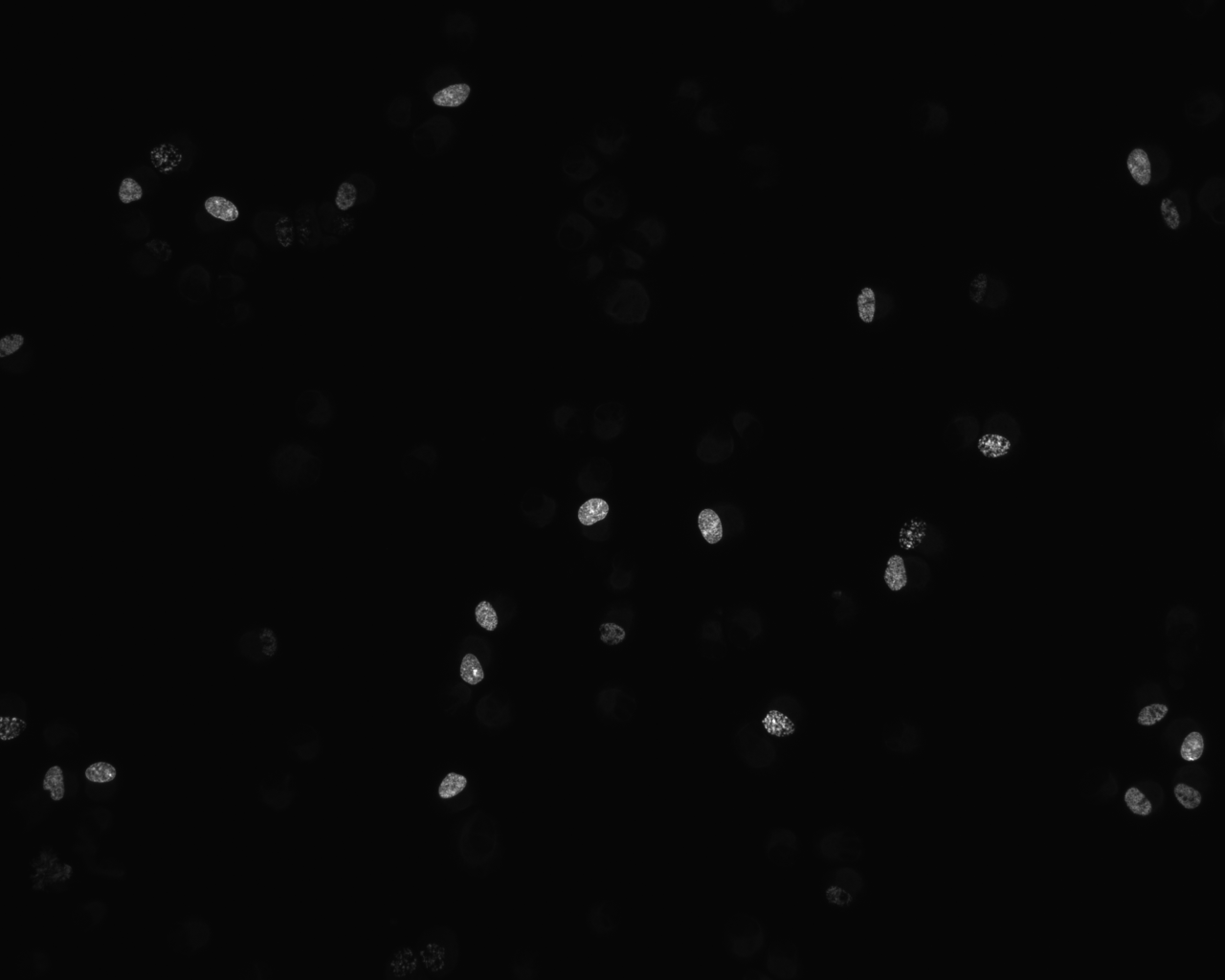

Supplement: S2 Data — (ZIP) [file pone.0233647.s005.zip › PACE Corrected/rSC49_SOX10AF488_EdUAF555_VIMEDL650_DAPI_20x_1_EdU 4.tif]

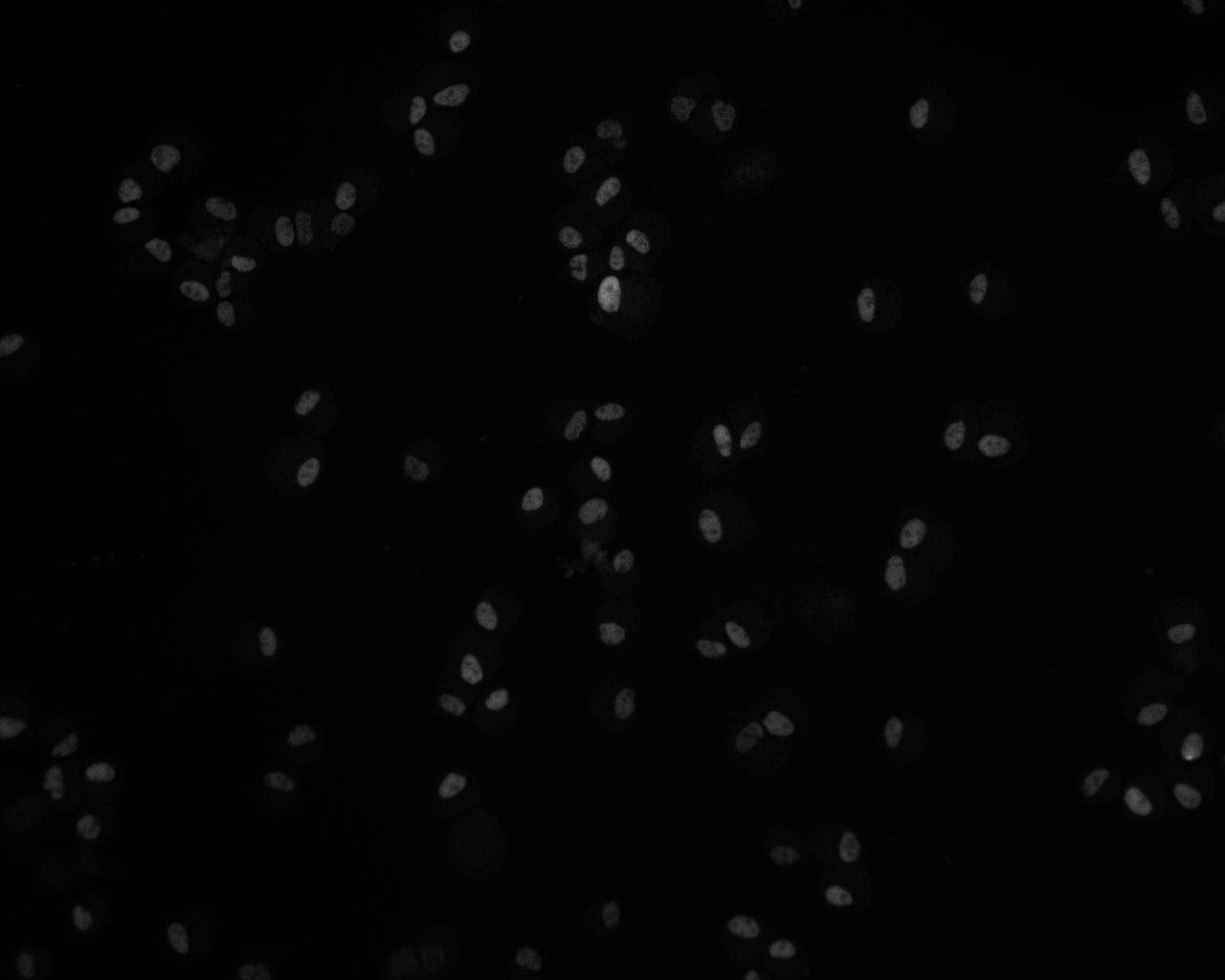

Supplement: S2 Data — (ZIP) [file pone.0233647.s005.zip › PACE Corrected/rSC49_SOX10AF488_EdUAF555_VIMEDL650_DAPI_20x_1_SOX10 2.tif]

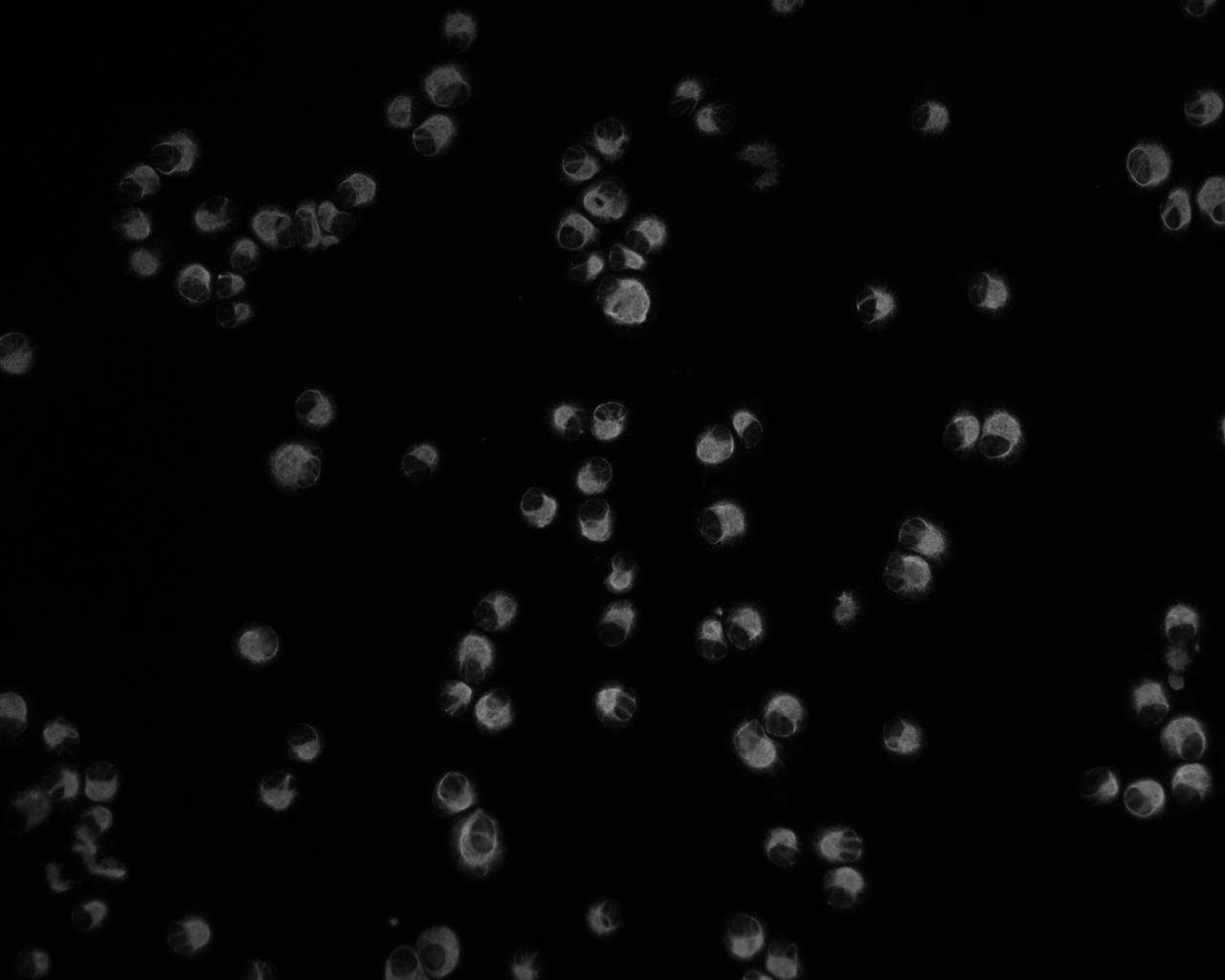

Supplement: S2 Data — (ZIP) [file pone.0233647.s005.zip › PACE Corrected/rSC49_SOX10AF488_EdUAF555_VIMEDL650_DAPI_20x_1_VIME 3.tif]

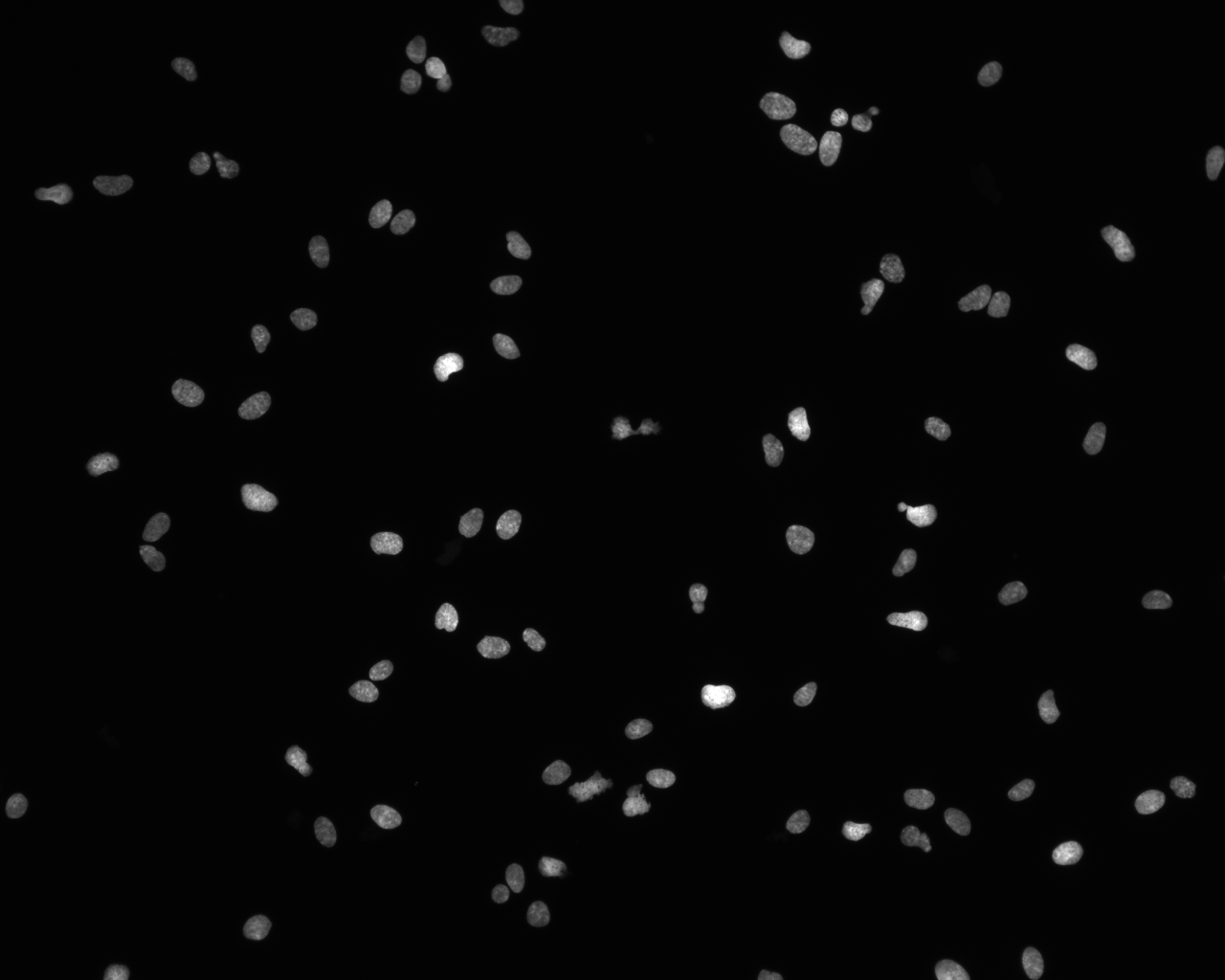

Supplement: S2 Data — (ZIP) [file pone.0233647.s005.zip › PACE Corrected/rSC49_SOX10AF488_EdUAF555_VIMEDL650_DAPI_20x_2_DAPI 1.tif]

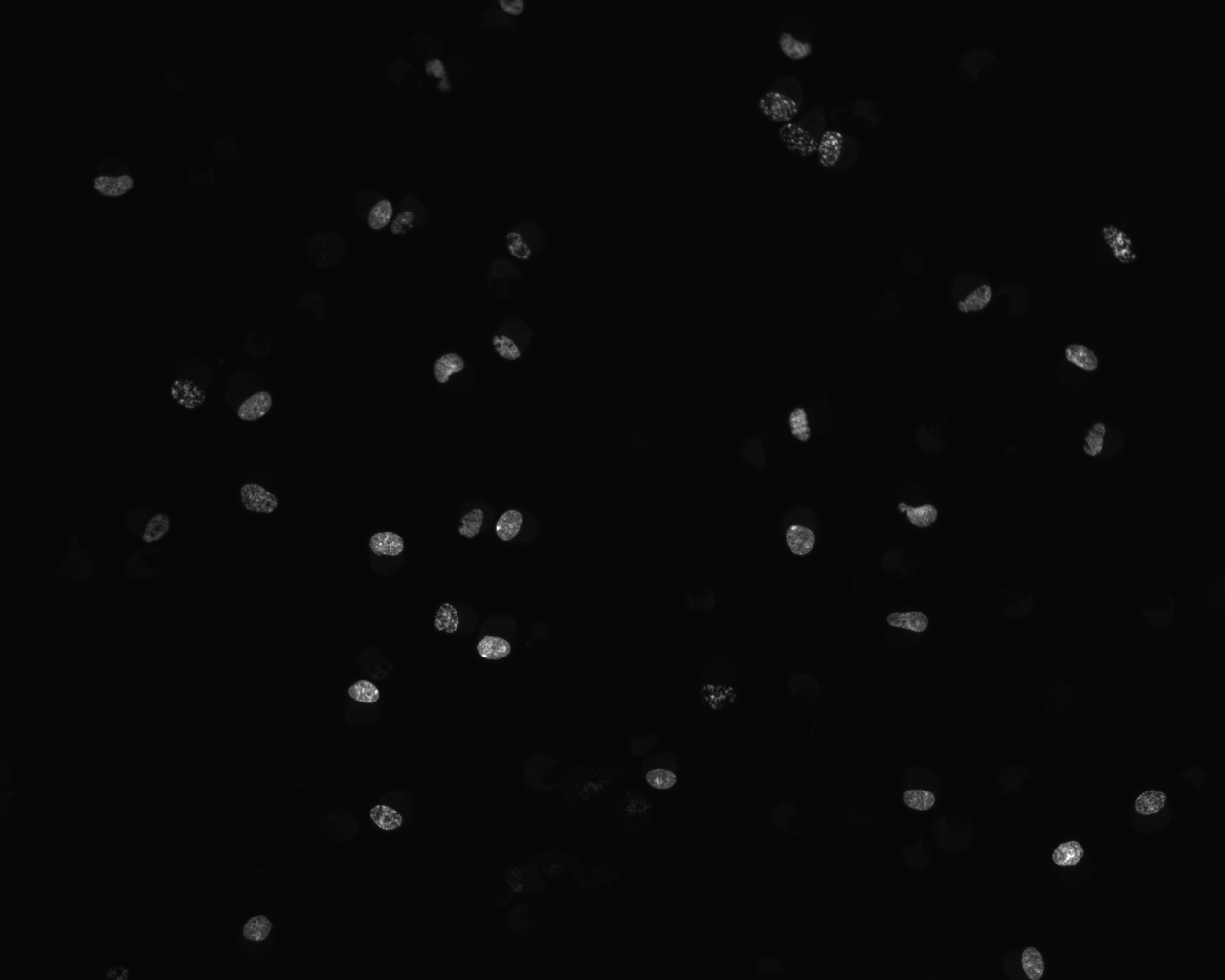

Supplement: S2 Data — (ZIP) [file pone.0233647.s005.zip › PACE Corrected/rSC49_SOX10AF488_EdUAF555_VIMEDL650_DAPI_20x_2_EdU 4.tif]

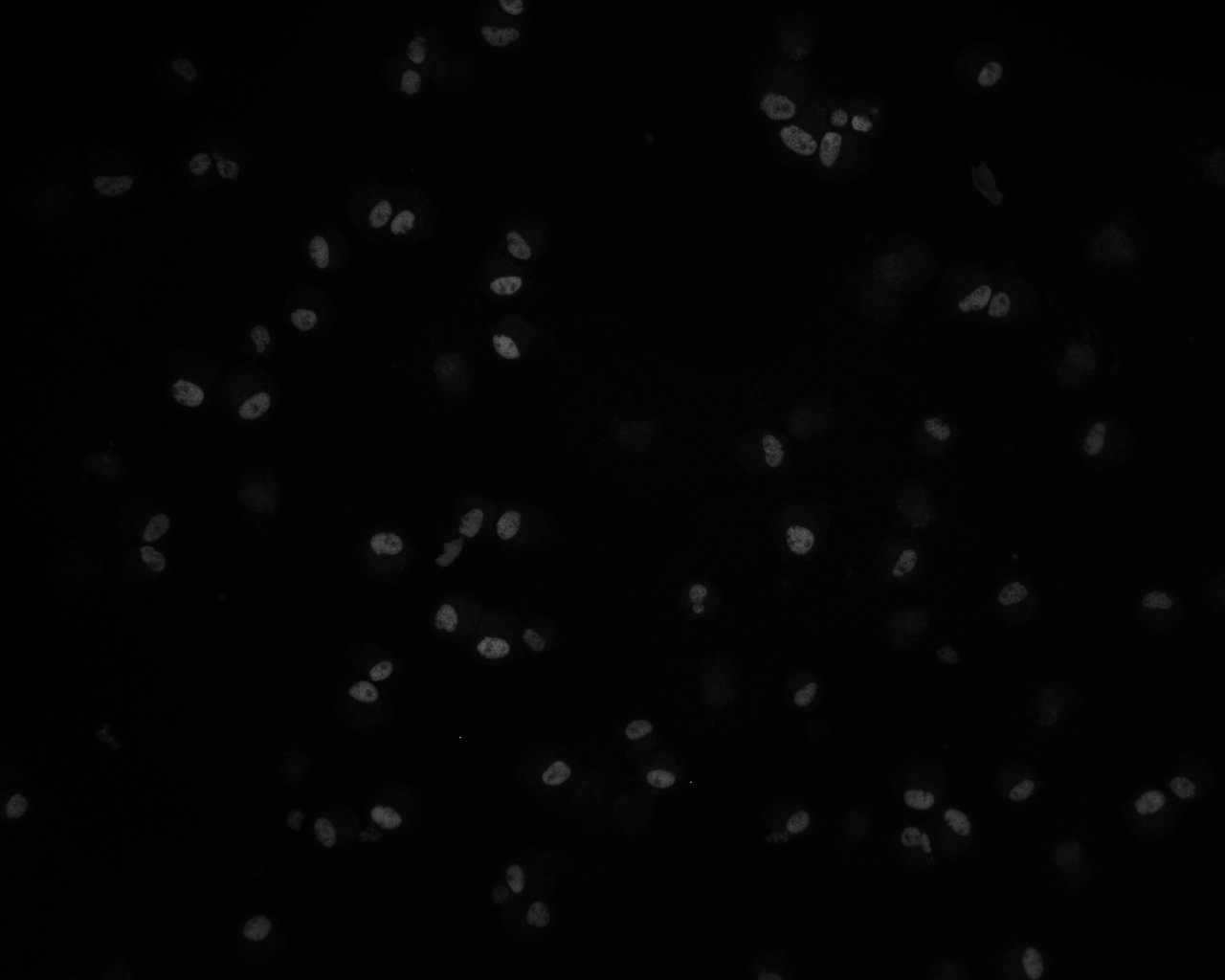

Supplement: S2 Data — (ZIP) [file pone.0233647.s005.zip › PACE Corrected/rSC49_SOX10AF488_EdUAF555_VIMEDL650_DAPI_20x_2_SOX10 2.tif]

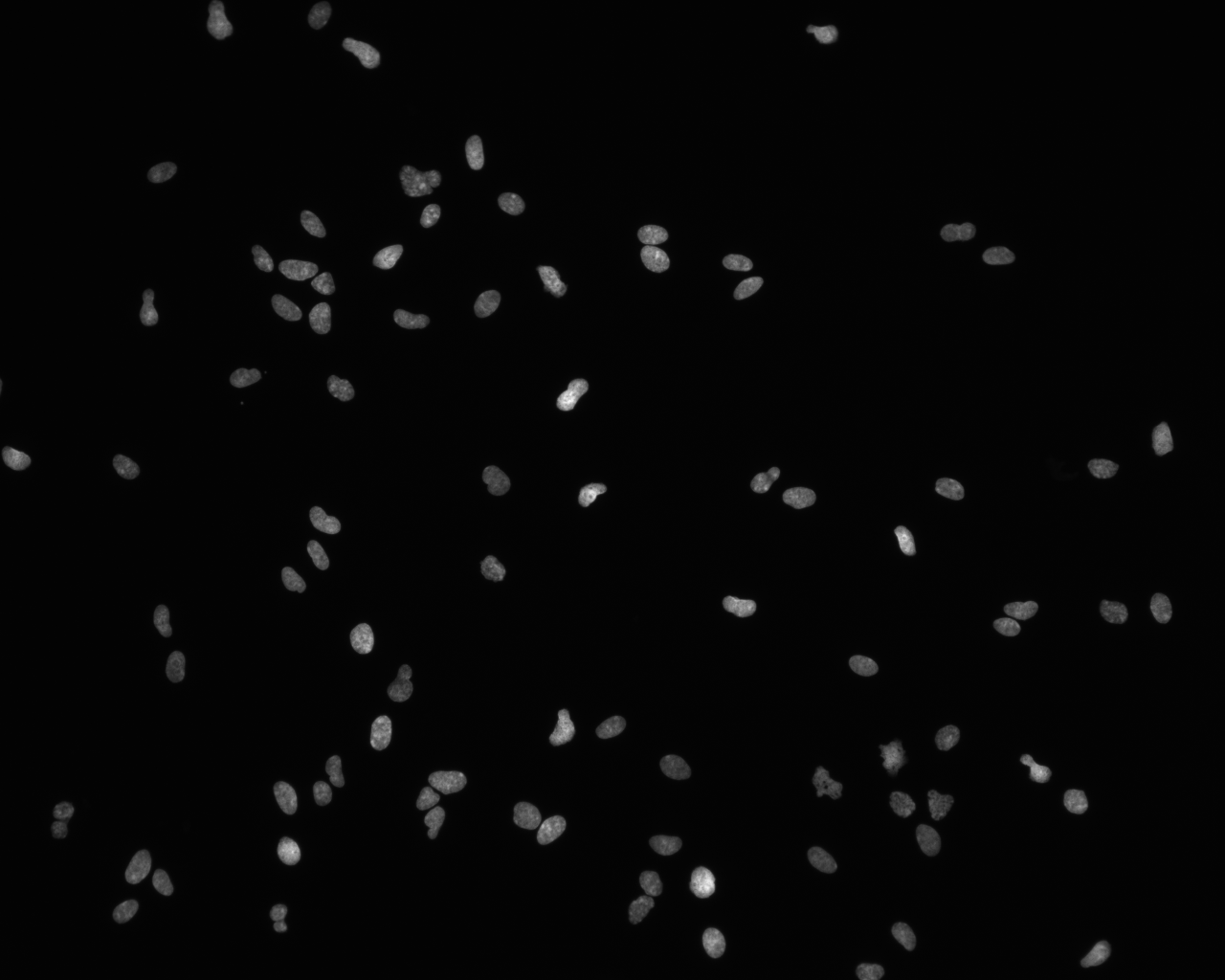

Supplement: S2 Data — (ZIP) [file pone.0233647.s005.zip › PACE Corrected/rSC49_SOX10AF488_EdUAF555_VIMEDL650_DAPI_20x_3_DAPI 1.tif]

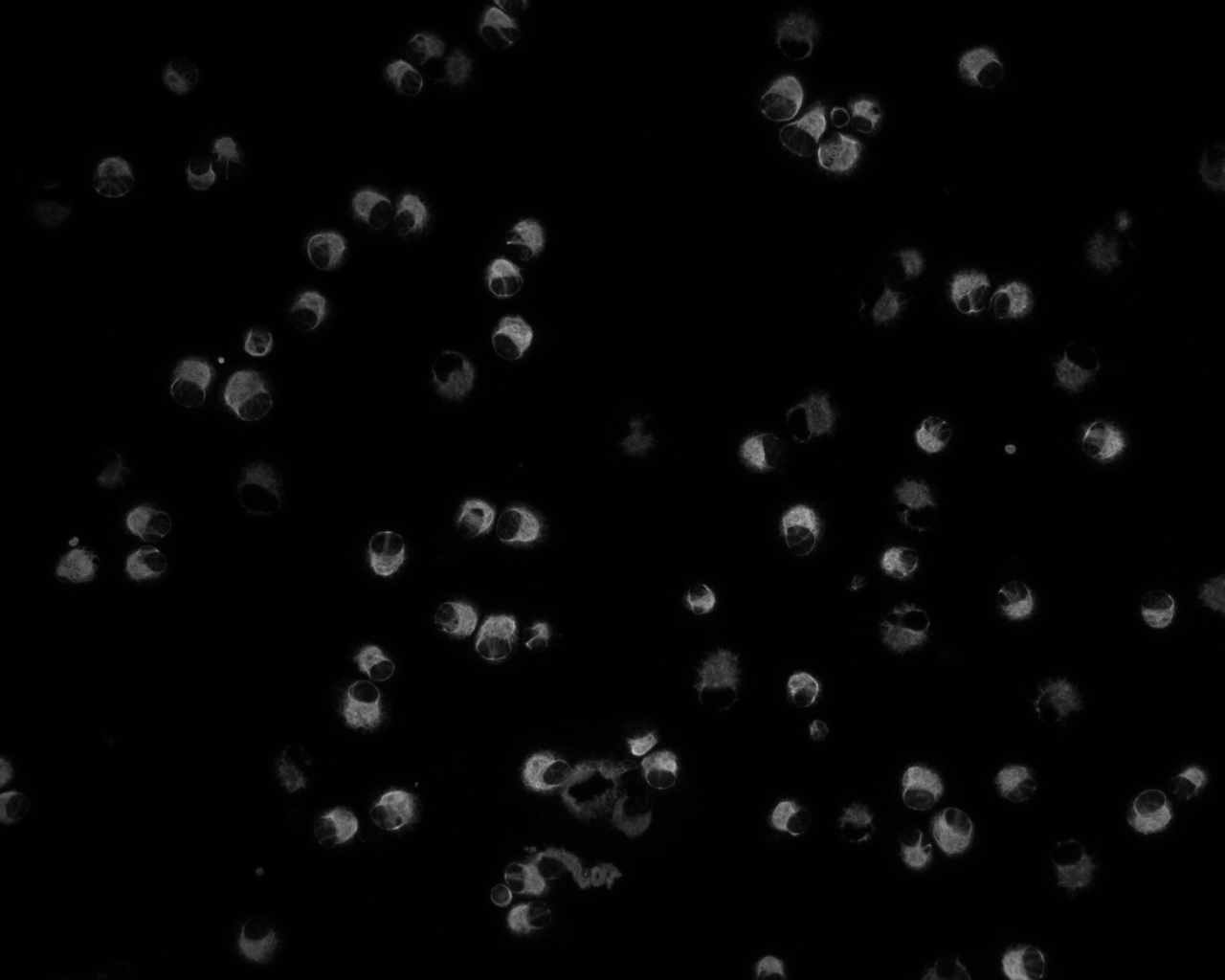

Supplement: S2 Data — (ZIP) [file pone.0233647.s005.zip › PACE Corrected/rSC49_SOX10AF488_EdUAF555_VIMEDL650_DAPI_20x_2_VIME 3.tif]

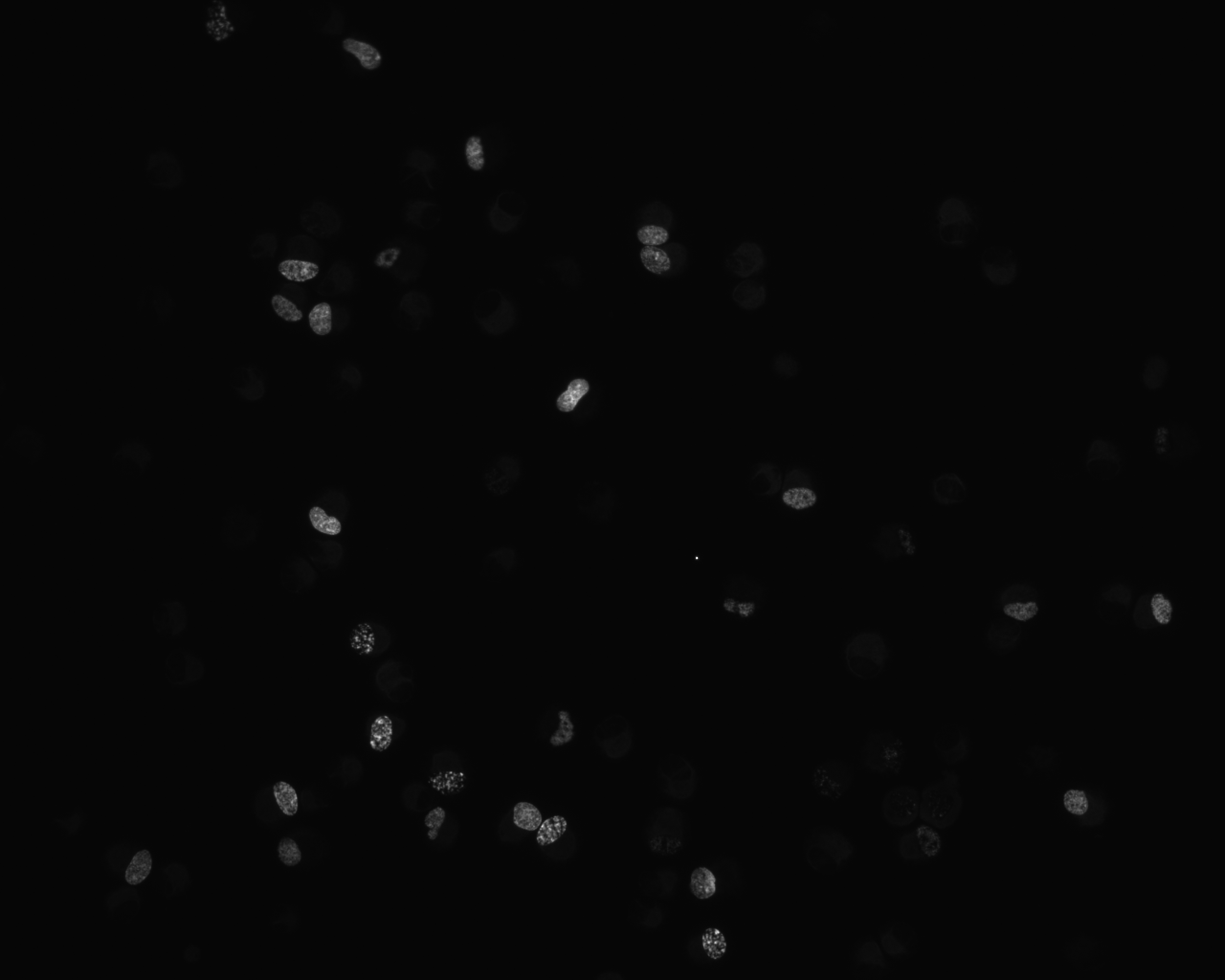

Supplement: S2 Data — (ZIP) [file pone.0233647.s005.zip › PACE Corrected/rSC49_SOX10AF488_EdUAF555_VIMEDL650_DAPI_20x_3_EdU 4.tif]

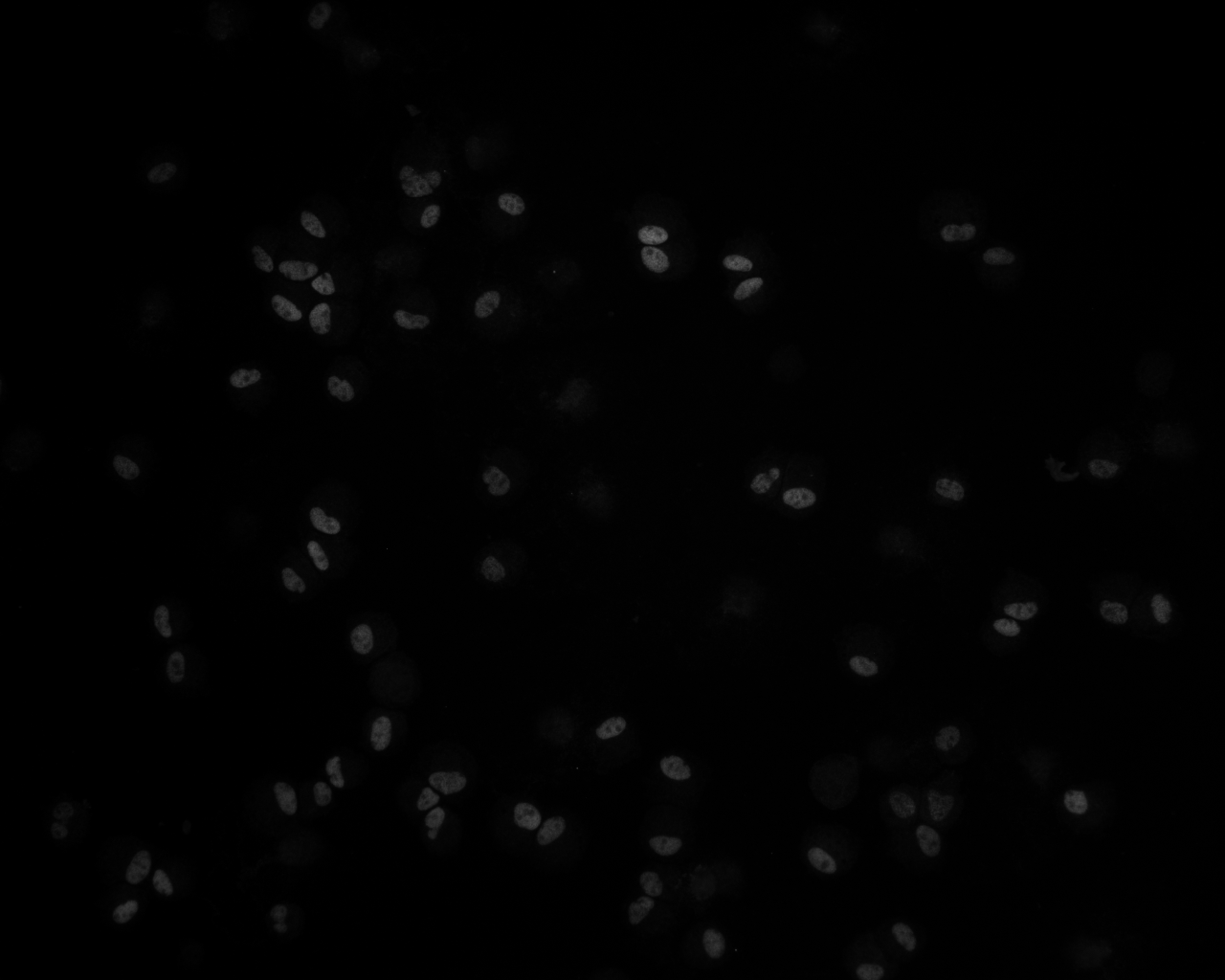

Supplement: S2 Data — (ZIP) [file pone.0233647.s005.zip › PACE Corrected/rSC49_SOX10AF488_EdUAF555_VIMEDL650_DAPI_20x_3_SOX10 2.tif]

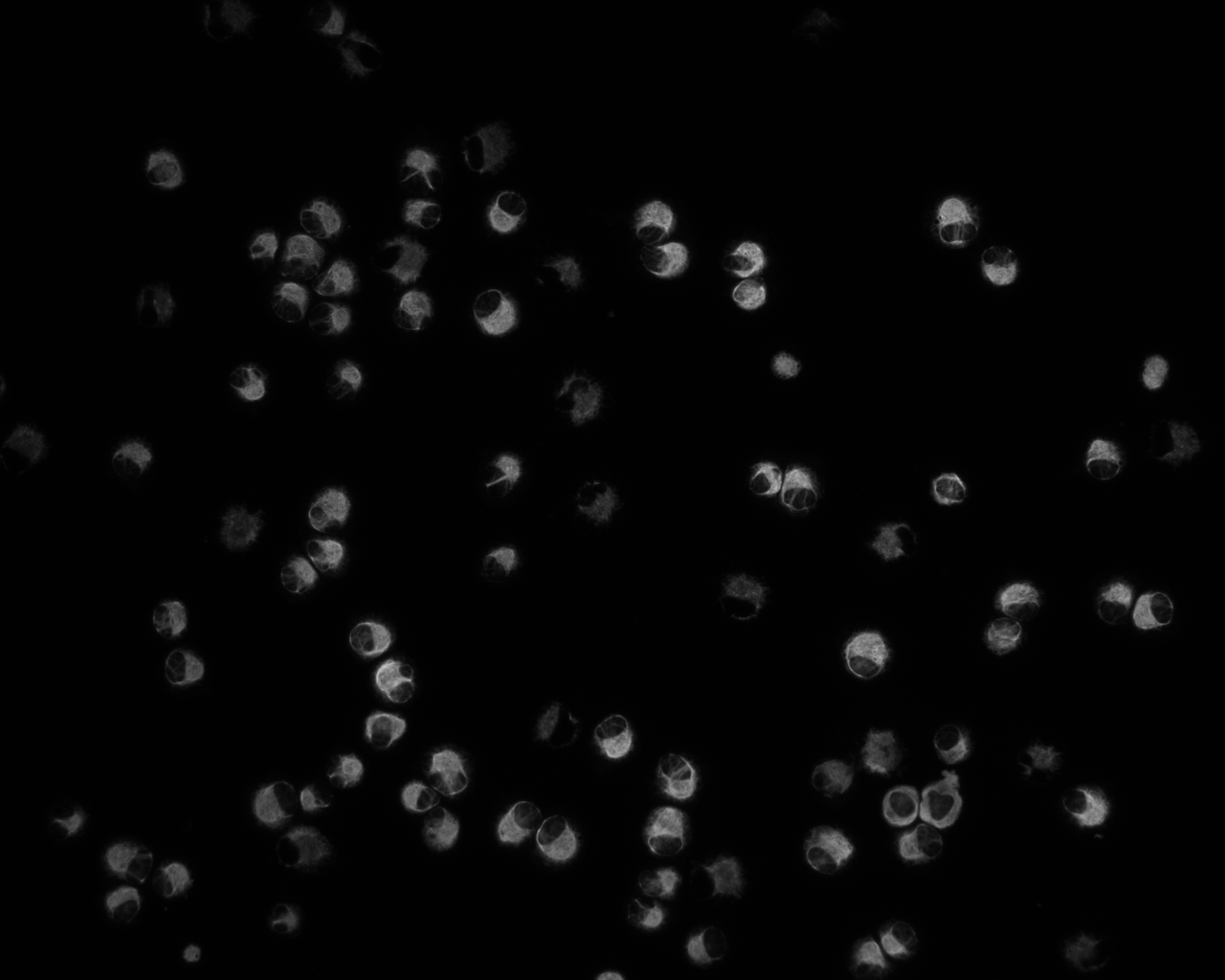

Supplement: S2 Data — (ZIP) [file pone.0233647.s005.zip › PACE Corrected/rSC49_SOX10AF488_EdUAF555_VIMEDL650_DAPI_20x_3_VIME 3.tif]

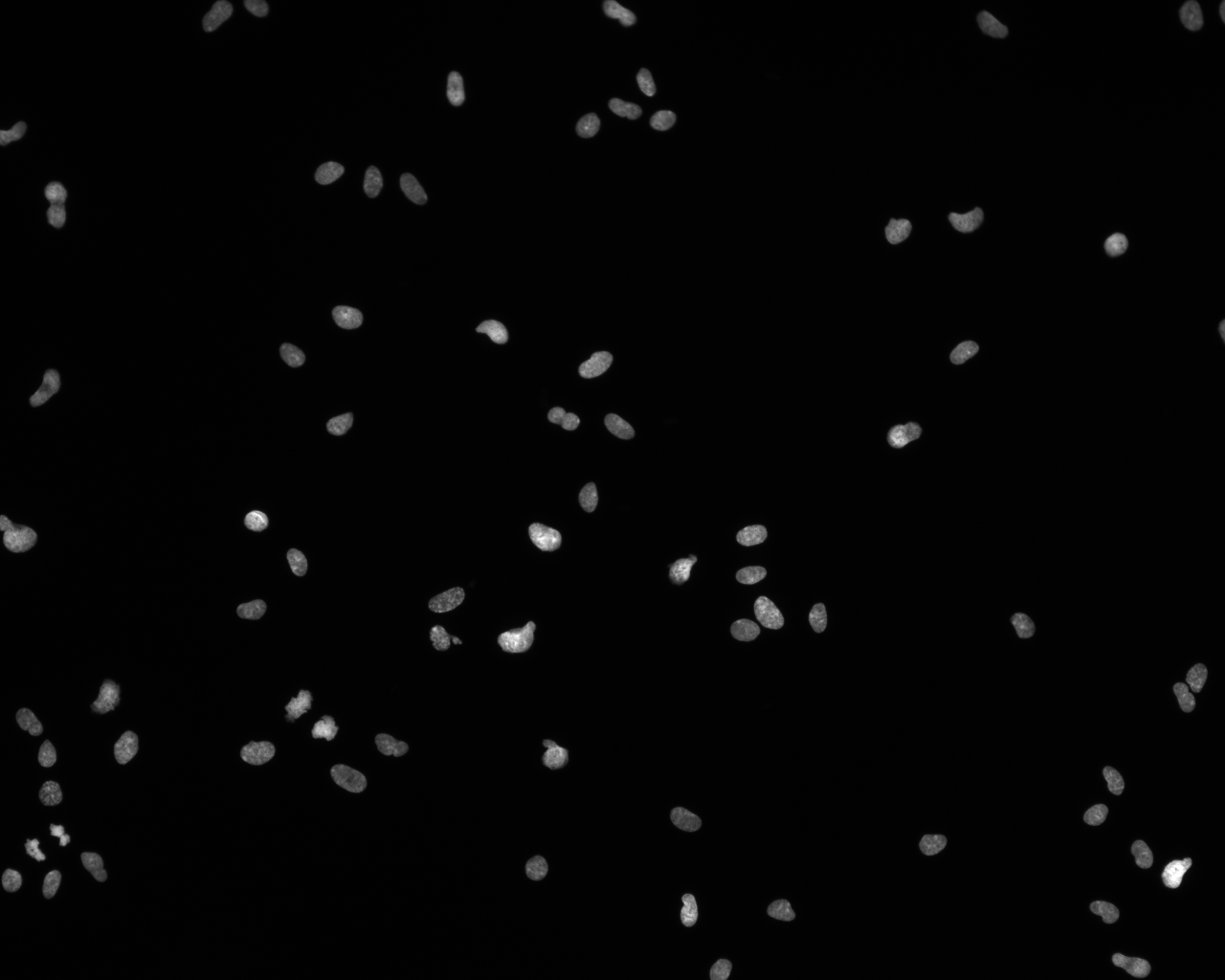

Supplement: S2 Data — (ZIP) [file pone.0233647.s005.zip › PACE Corrected/rSC49_SOX10AF488_EdUAF555_VIMEDL650_DAPI_20x_4_DAPI 1.tif]

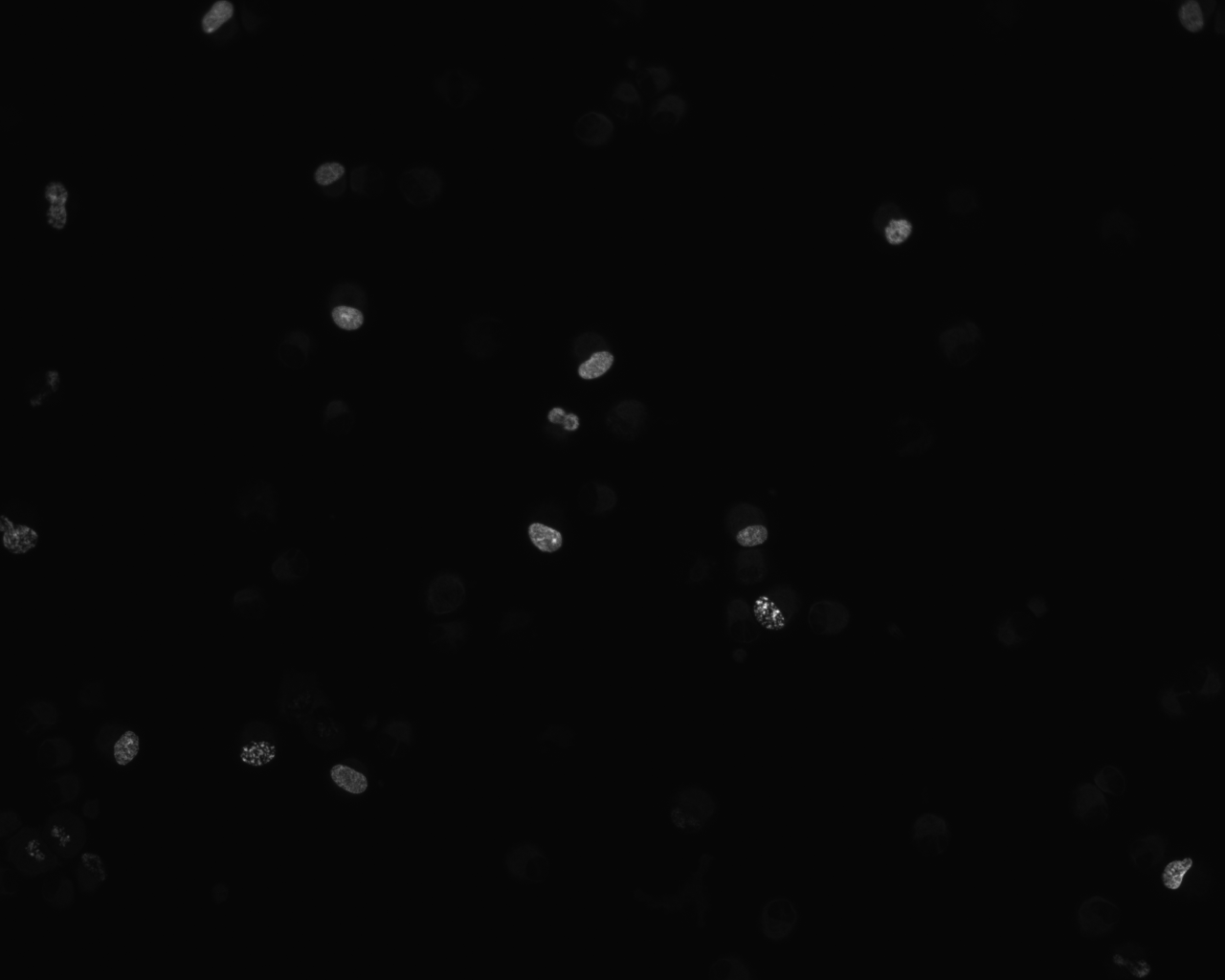

Supplement: S2 Data — (ZIP) [file pone.0233647.s005.zip › PACE Corrected/rSC49_SOX10AF488_EdUAF555_VIMEDL650_DAPI_20x_4_EdU 4.tif]

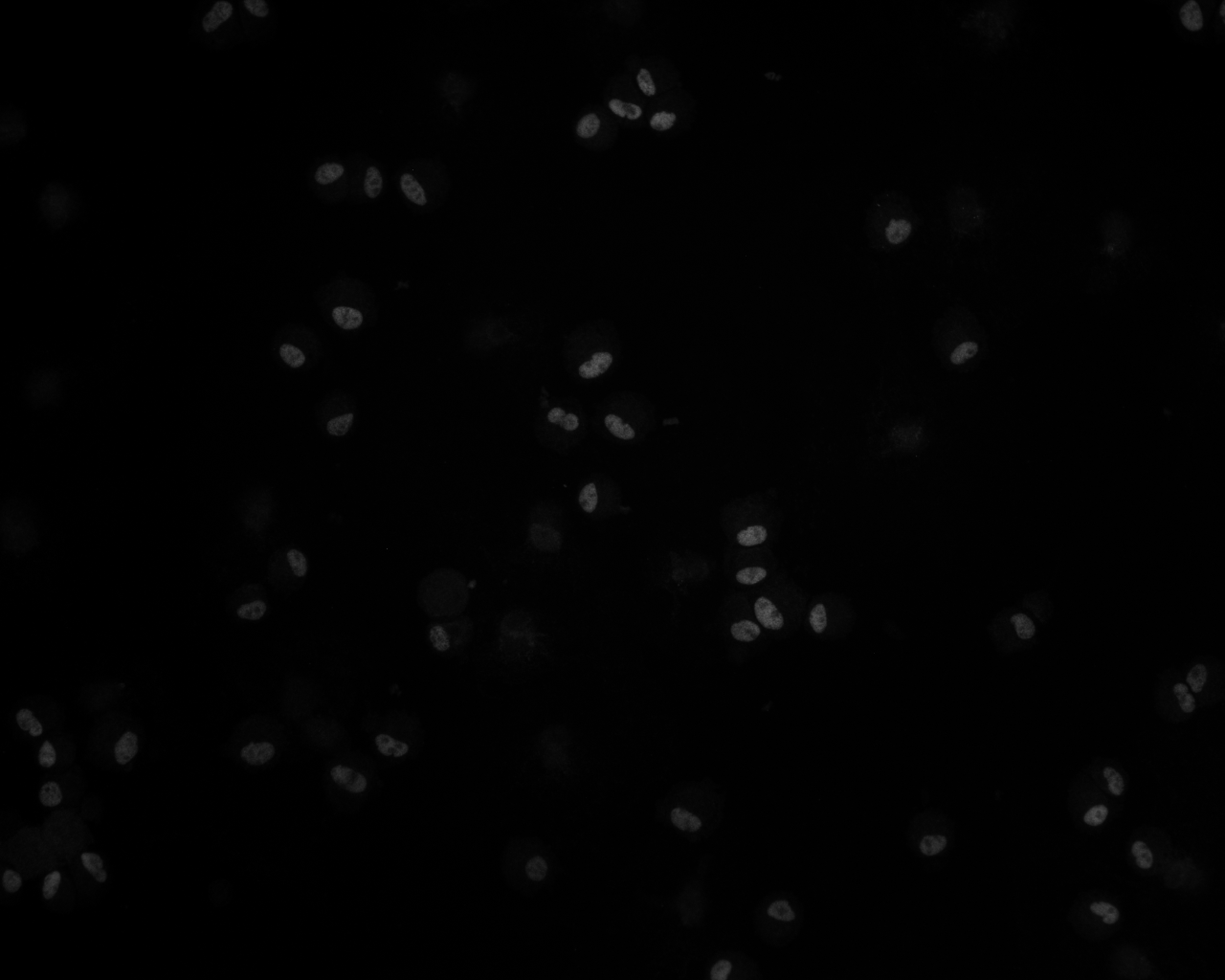

Supplement: S2 Data — (ZIP) [file pone.0233647.s005.zip › PACE Corrected/rSC49_SOX10AF488_EdUAF555_VIMEDL650_DAPI_20x_4_SOX10 2.tif]

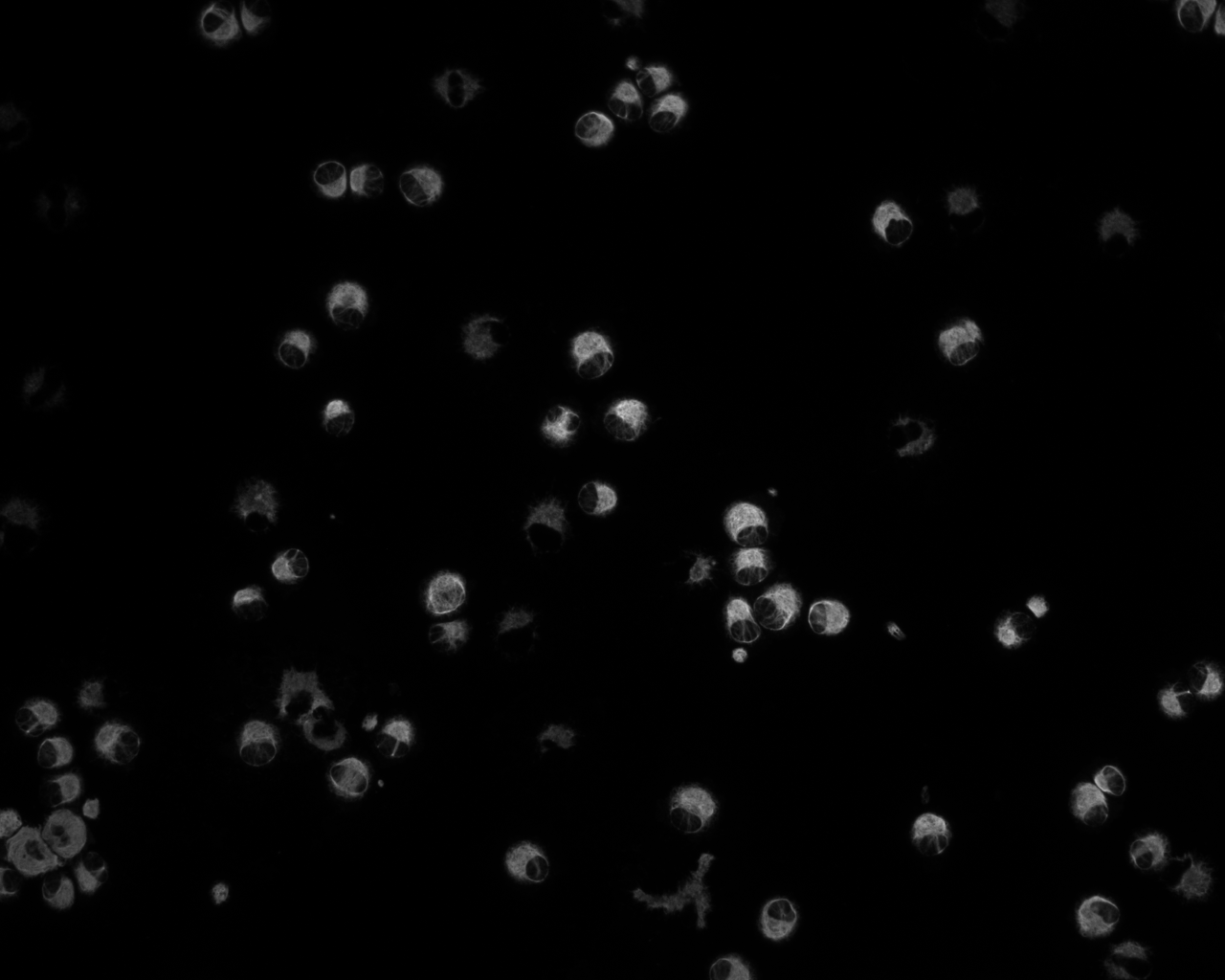

Supplement: S2 Data — (ZIP) [file pone.0233647.s005.zip › PACE Corrected/rSC49_SOX10AF488_EdUAF555_VIMEDL650_DAPI_20x_4_VIME 3.tif]

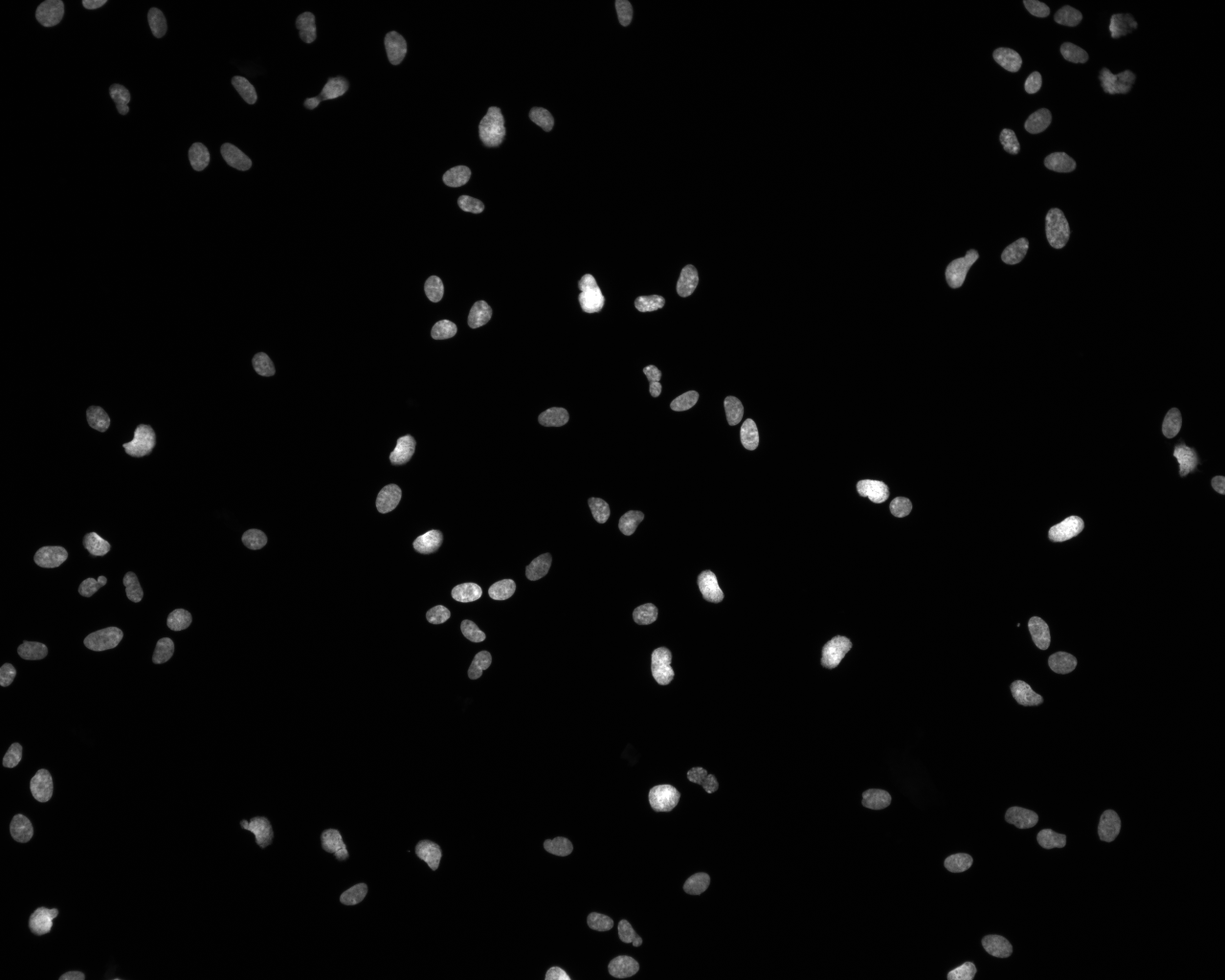

Supplement: S2 Data — (ZIP) [file pone.0233647.s005.zip › PACE Corrected/rSC49_SOX10AF488_EdUAF555_VIMEDL650_DAPI_20x_5_DAPI 1.tif]

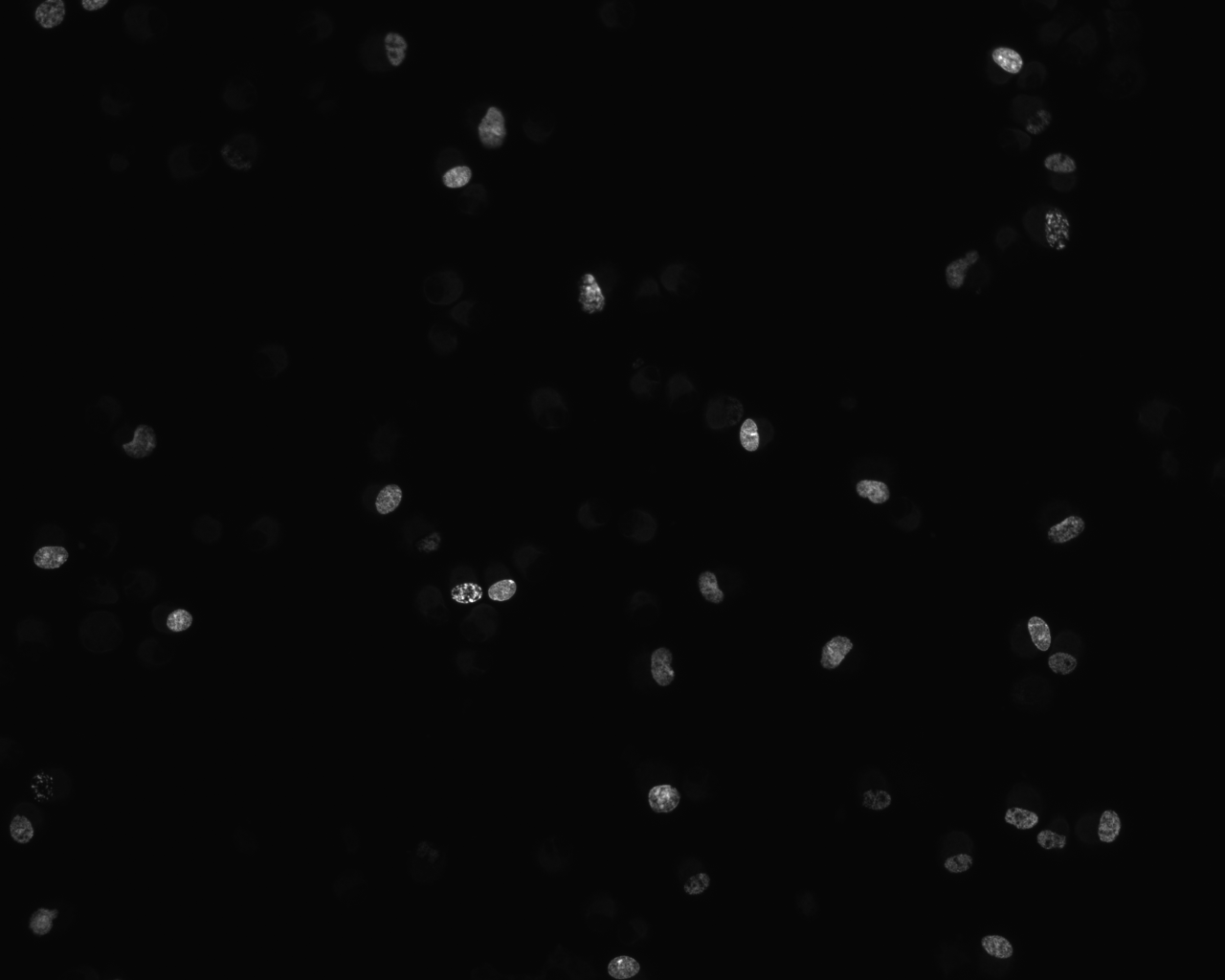

Supplement: S2 Data — (ZIP) [file pone.0233647.s005.zip › PACE Corrected/rSC49_SOX10AF488_EdUAF555_VIMEDL650_DAPI_20x_5_EdU 4.tif]

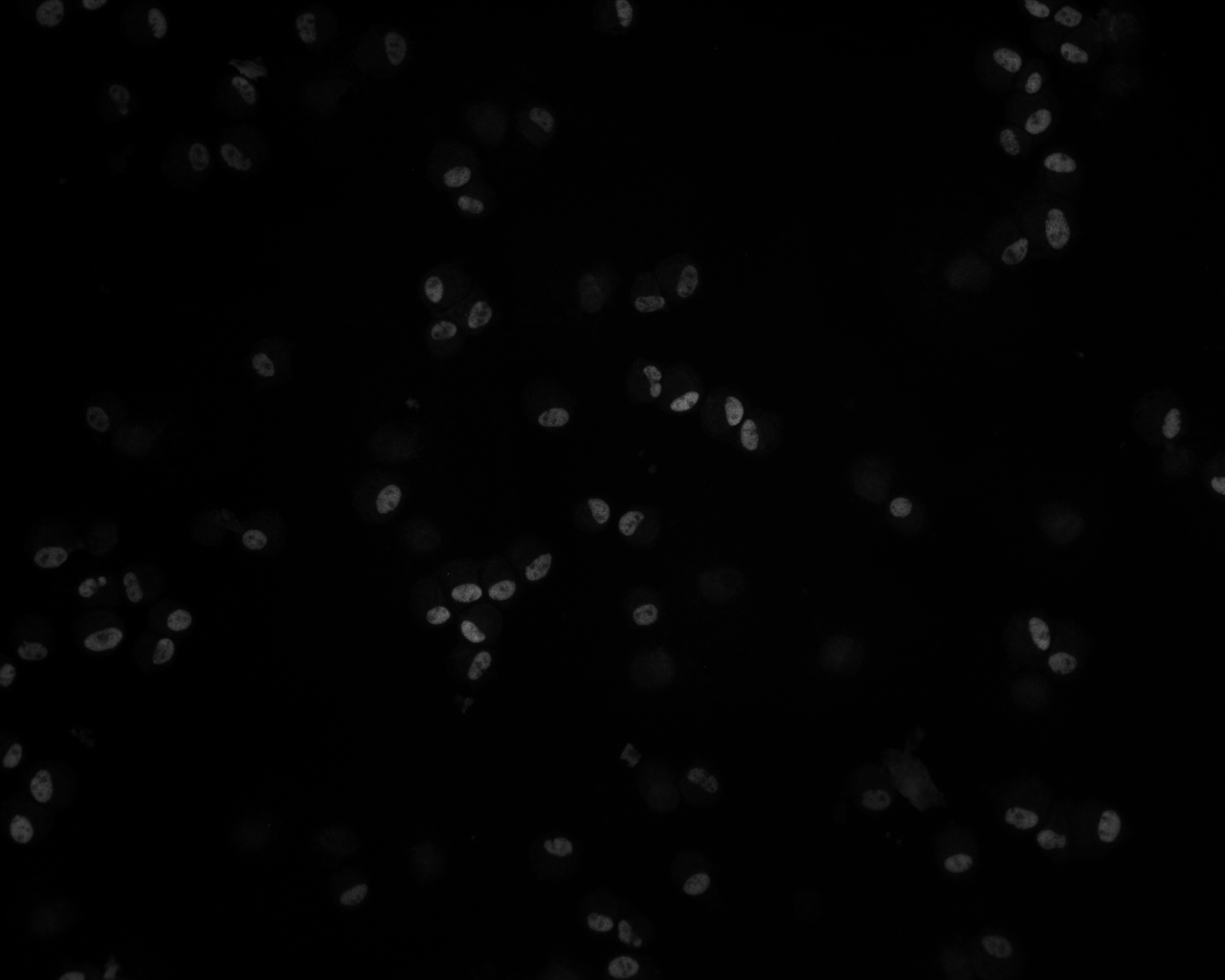

Supplement: S2 Data — (ZIP) [file pone.0233647.s005.zip › PACE Corrected/rSC49_SOX10AF488_EdUAF555_VIMEDL650_DAPI_20x_5_SOX10 2.tif]

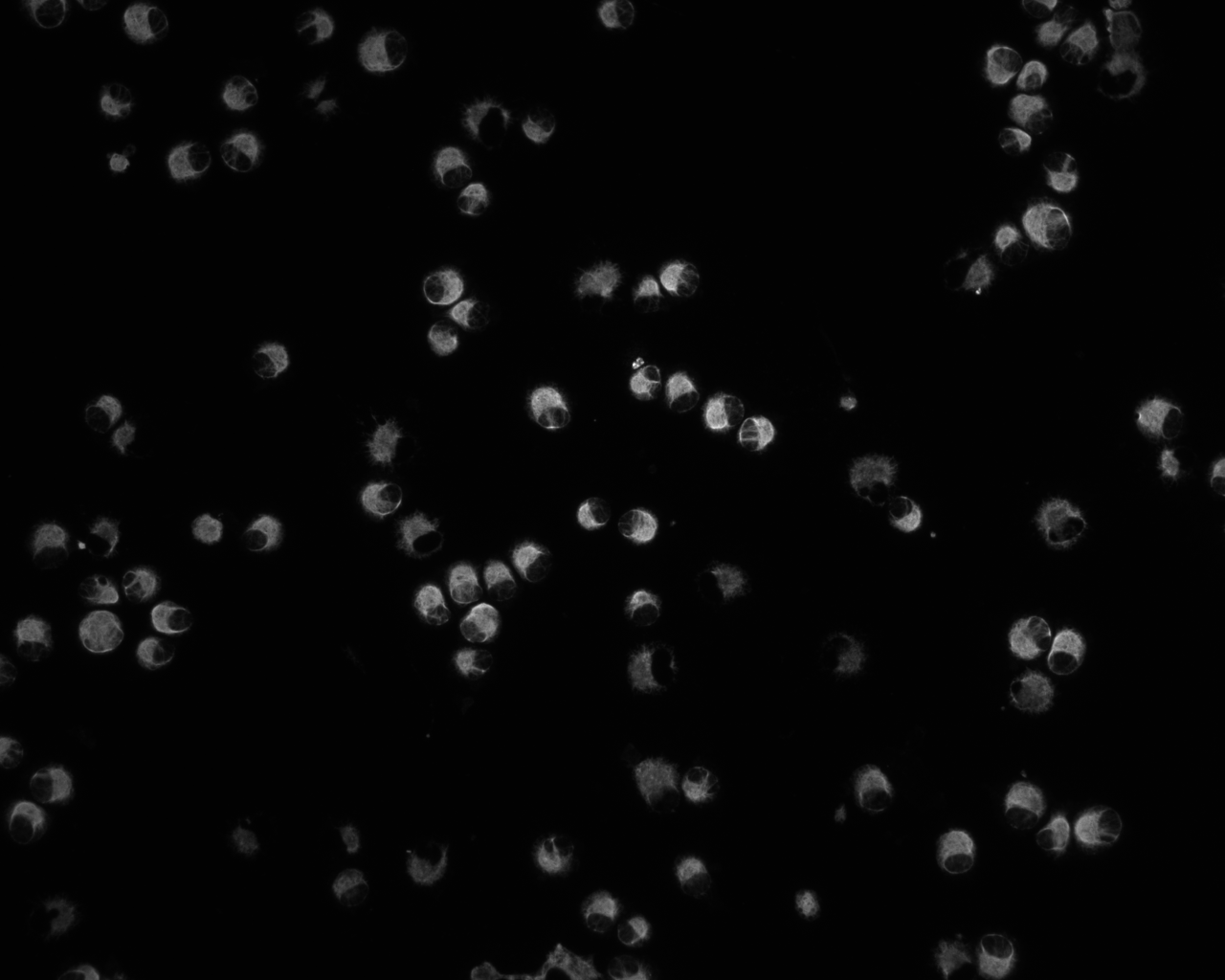

Supplement: S2 Data — (ZIP) [file pone.0233647.s005.zip › PACE Corrected/rSC49_SOX10AF488_EdUAF555_VIMEDL650_DAPI_20x_5_VIME 3.tif]

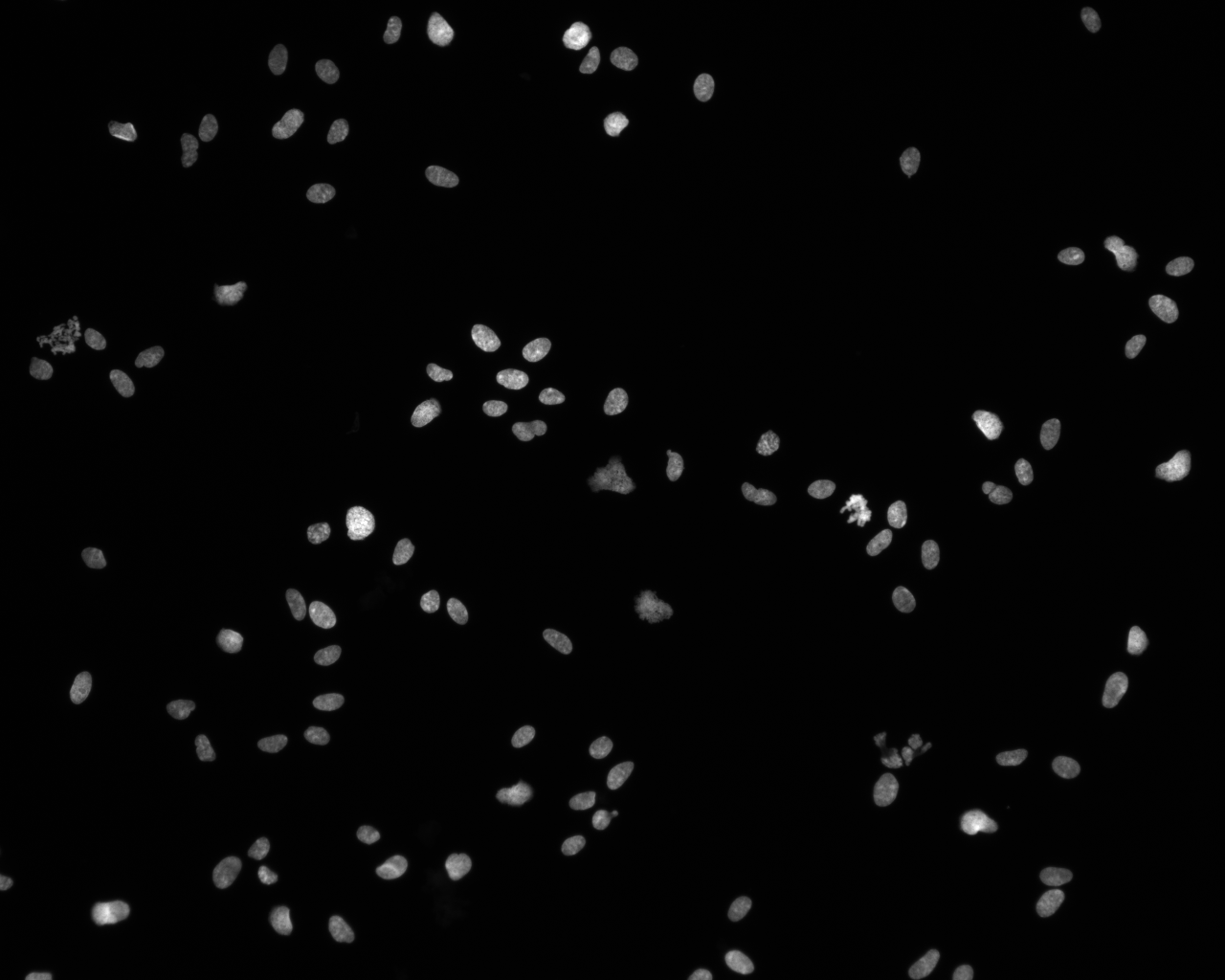

Supplement: S2 Data — (ZIP) [file pone.0233647.s005.zip › PACE Corrected/rSC49_SOX10AF488_EdUAF555_VIMEDL650_DAPI_20x_6_DAPI 1.tif]

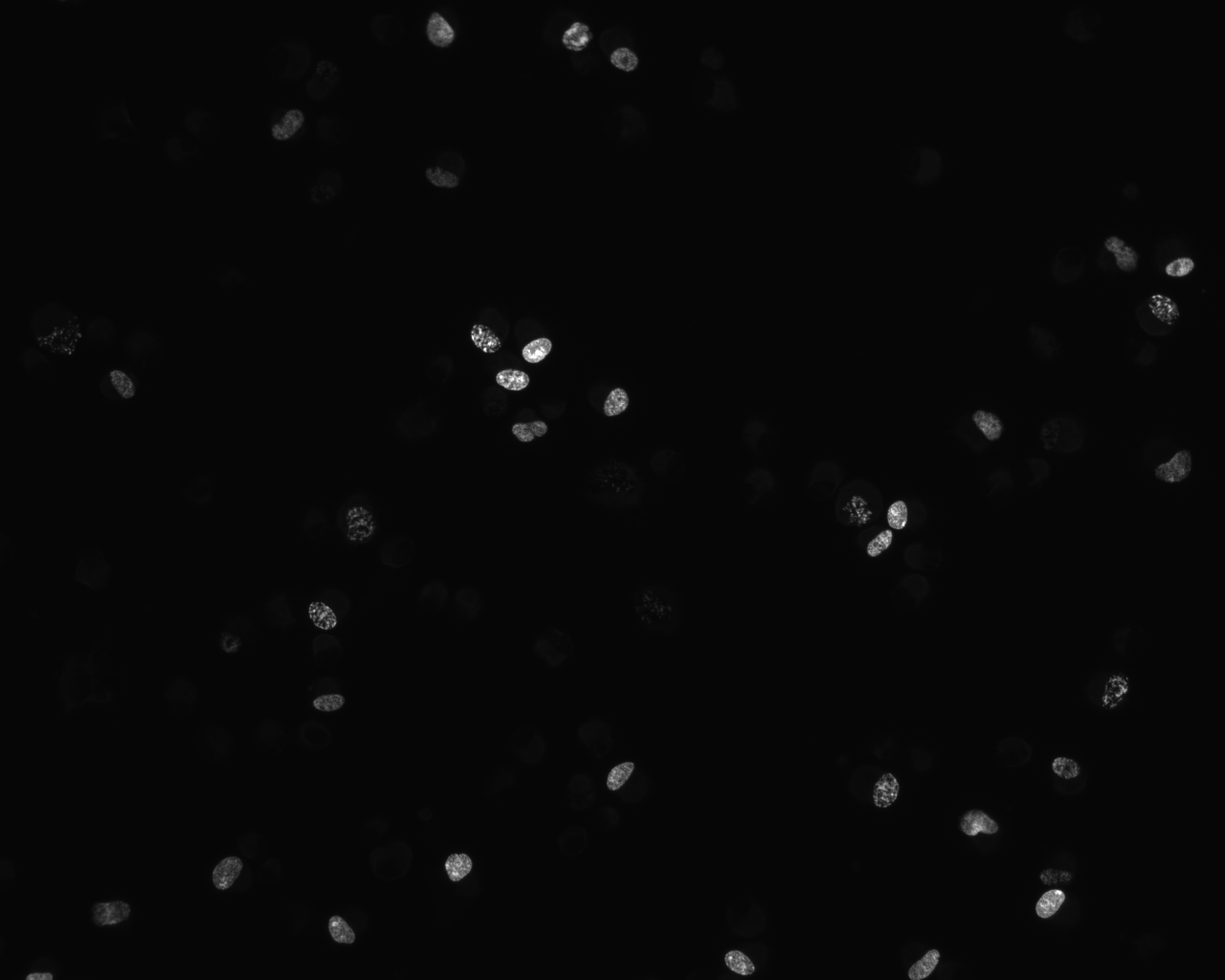

Supplement: S2 Data — (ZIP) [file pone.0233647.s005.zip › PACE Corrected/rSC49_SOX10AF488_EdUAF555_VIMEDL650_DAPI_20x_6_EdU 4.tif]

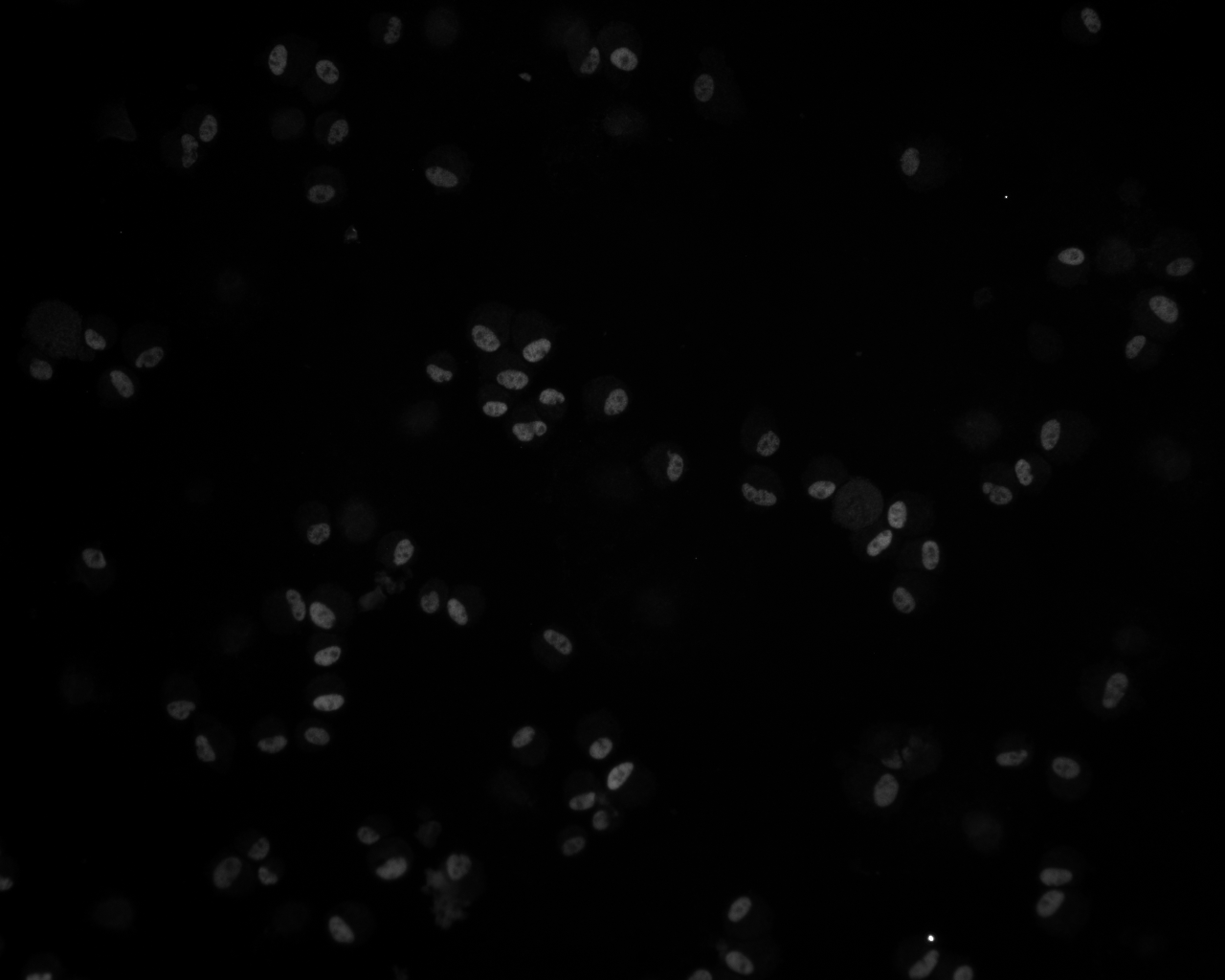

Supplement: S2 Data — (ZIP) [file pone.0233647.s005.zip › PACE Corrected/rSC49_SOX10AF488_EdUAF555_VIMEDL650_DAPI_20x_6_SOX10 2.tif]

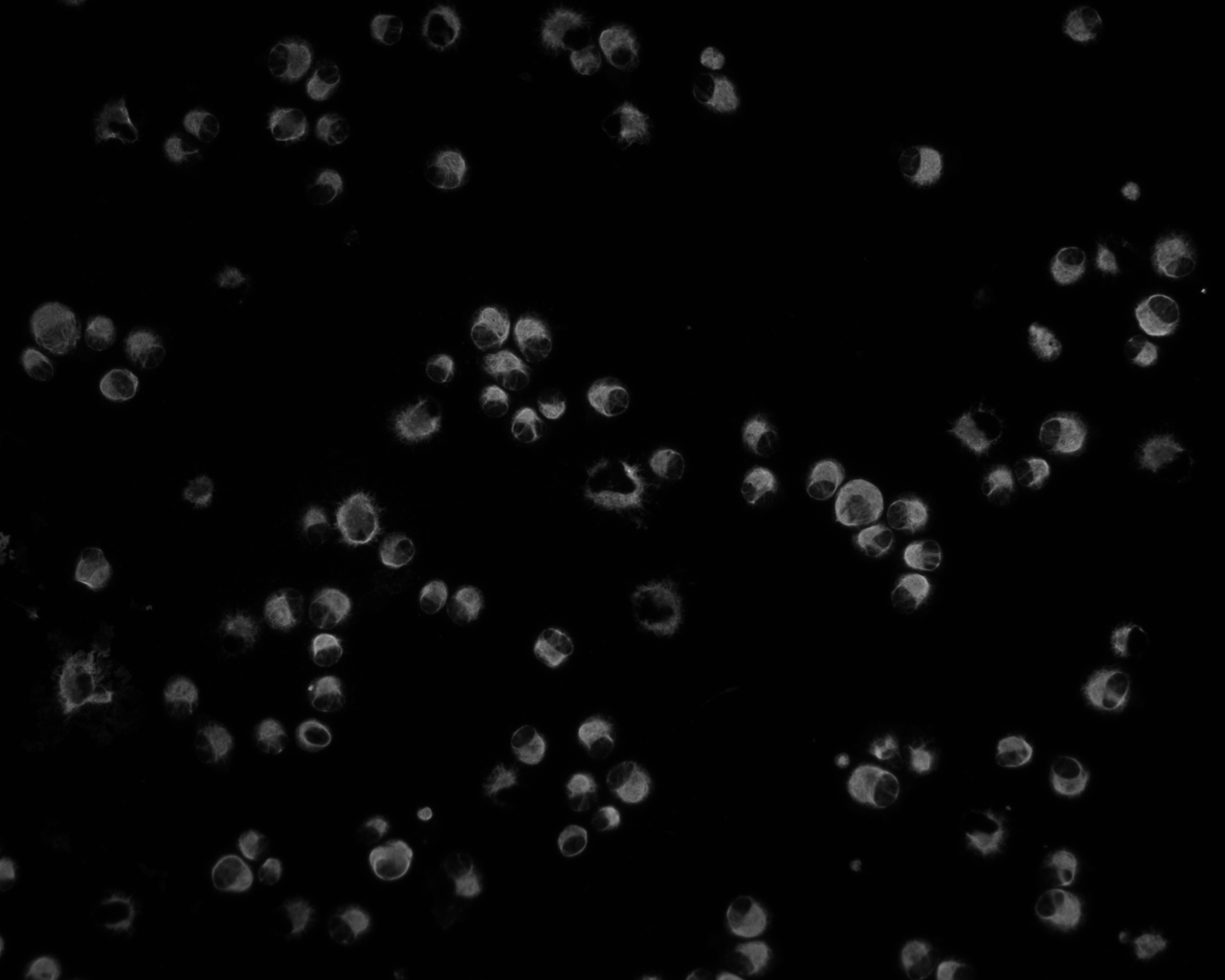

Supplement: S2 Data — (ZIP) [file pone.0233647.s005.zip › PACE Corrected/rSC49_SOX10AF488_EdUAF555_VIMEDL650_DAPI_20x_6_VIME 3.tif]

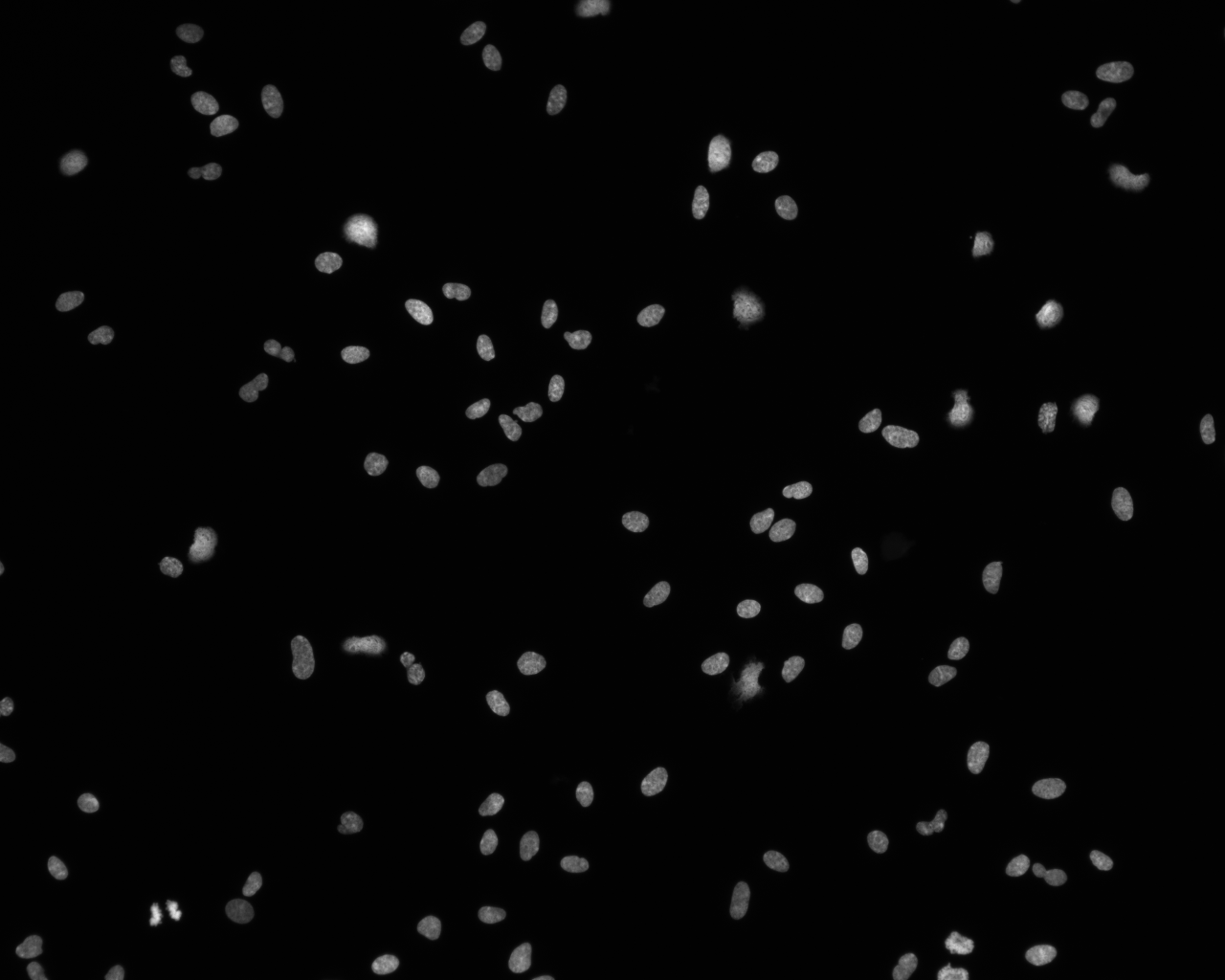

Supplement: S2 Data — (ZIP) [file pone.0233647.s005.zip › PACE Corrected/rSC49_SOX10AF488_EdUAF555_VIMEDL650_DAPI_20x_8_DAPI 1.tif]

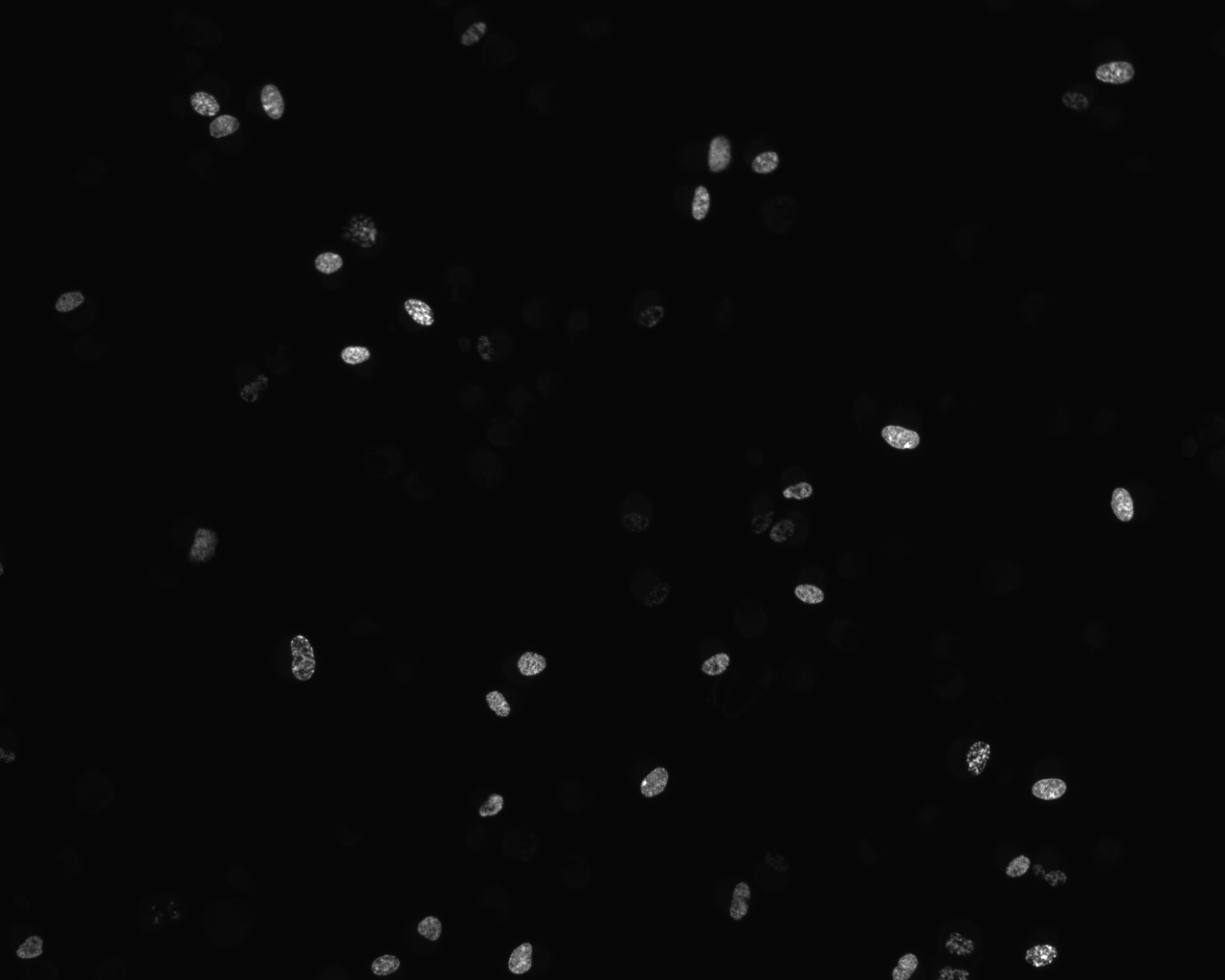

Supplement: S2 Data — (ZIP) [file pone.0233647.s005.zip › PACE Corrected/rSC49_SOX10AF488_EdUAF555_VIMEDL650_DAPI_20x_8_EdU 4.tif]

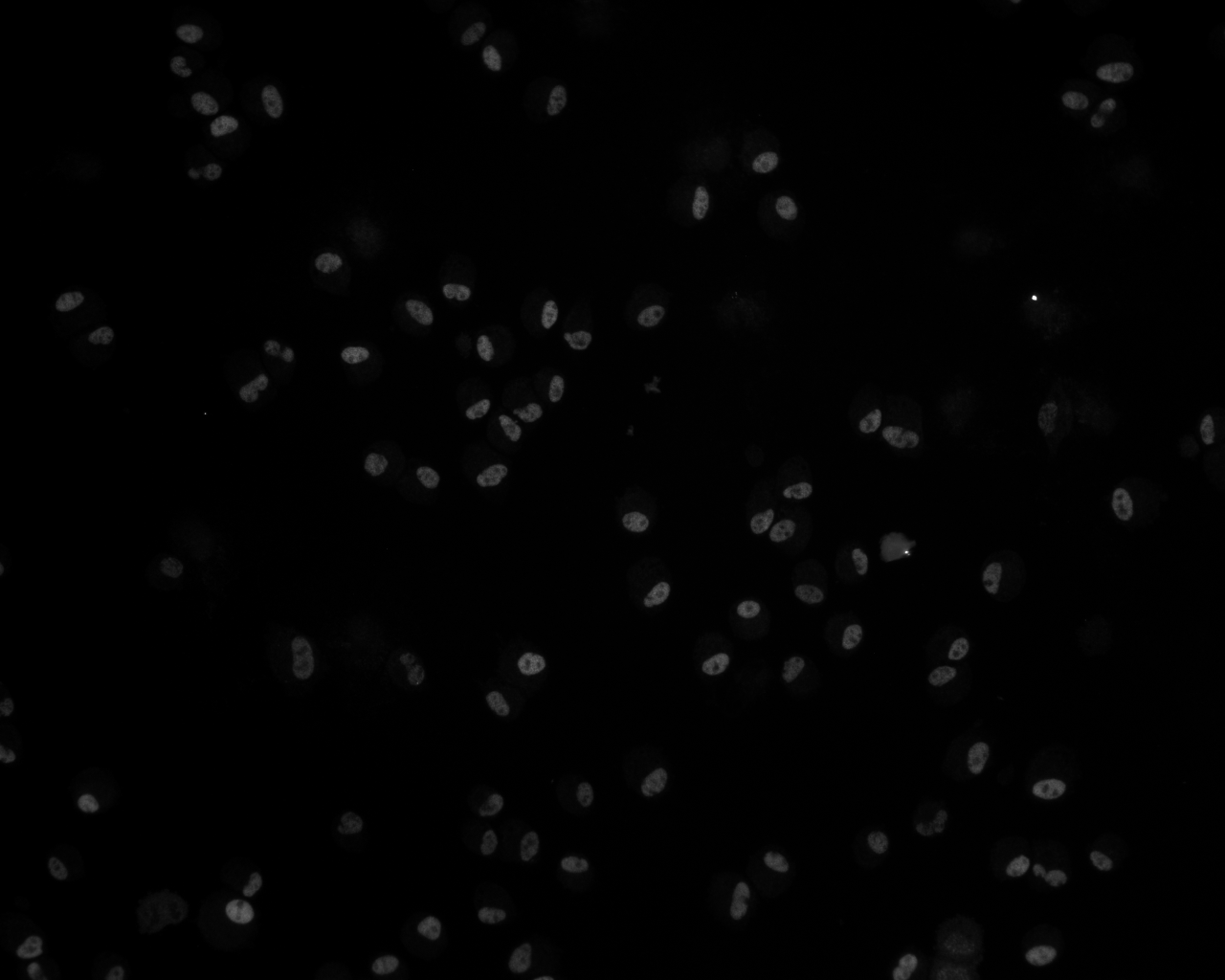

Supplement: S2 Data — (ZIP) [file pone.0233647.s005.zip › PACE Corrected/rSC49_SOX10AF488_EdUAF555_VIMEDL650_DAPI_20x_8_SOX10 2.tif]

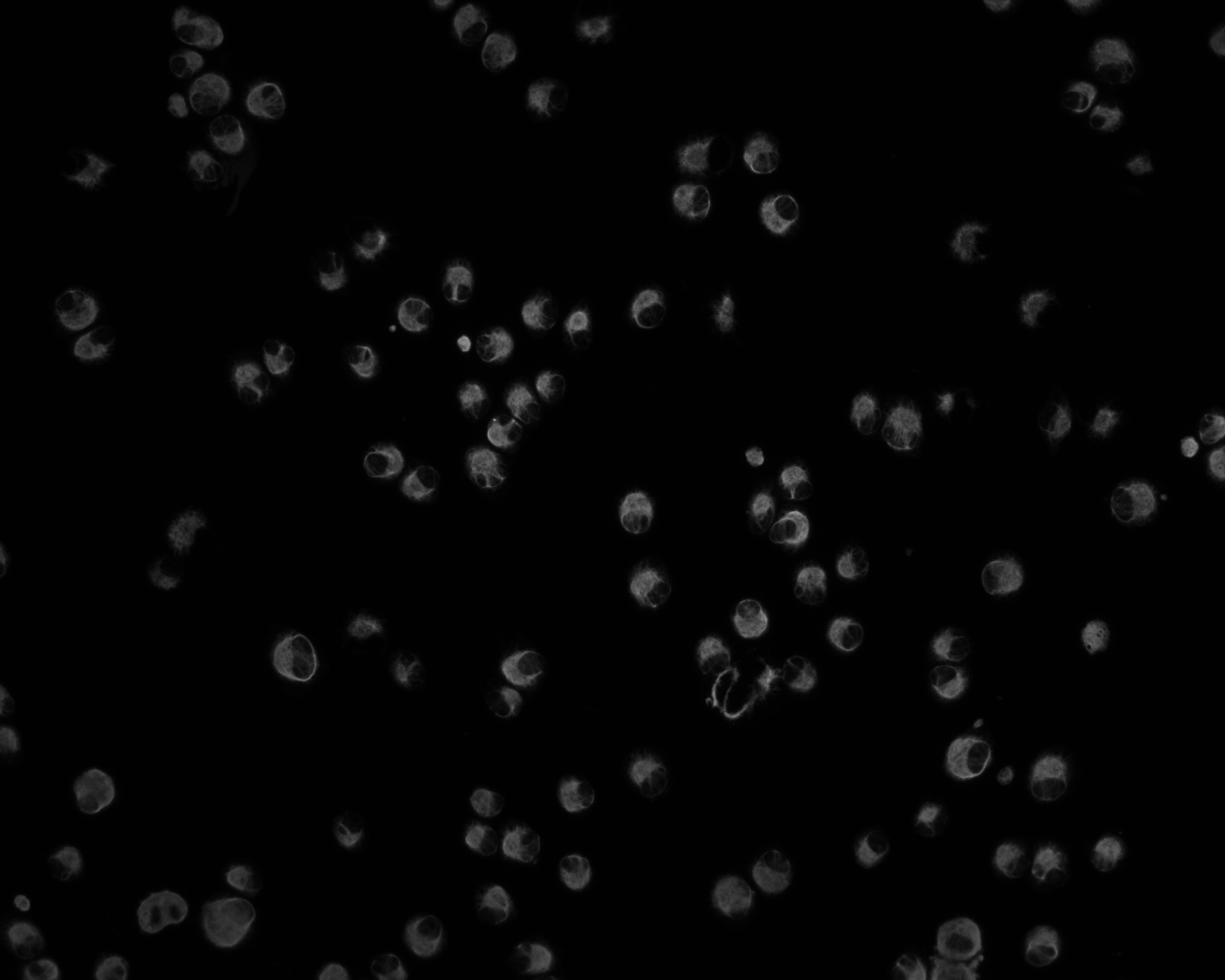

Supplement: S2 Data — (ZIP) [file pone.0233647.s005.zip › PACE Corrected/rSC49_SOX10AF488_EdUAF555_VIMEDL650_DAPI_20x_8_VIME 3.tif]

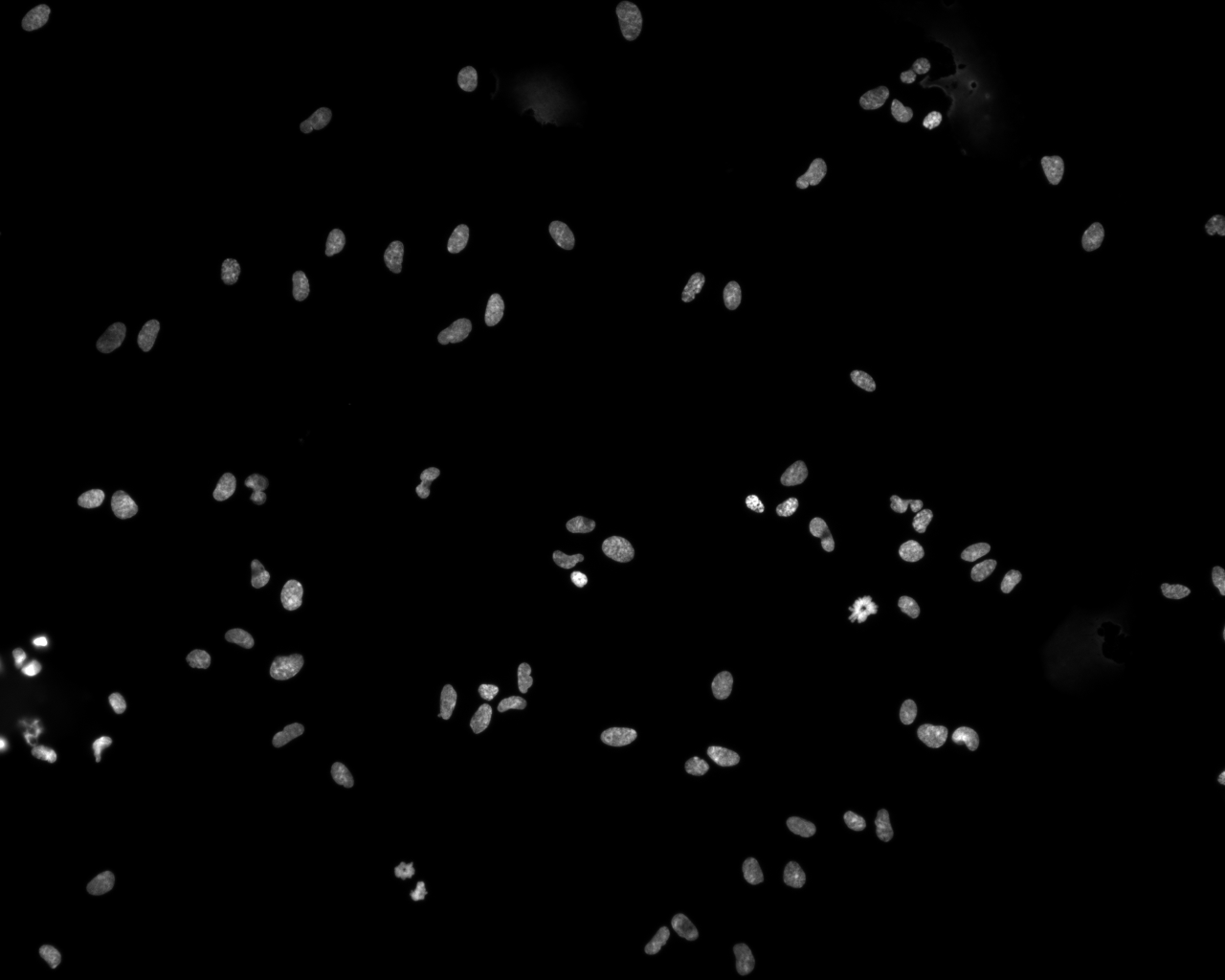

Supplement: S3 Data — (ZIP) [file pone.0233647.s006.zip › PACE Corrected/rSC51_SOX10AF488_EdUAF555_VIMEDL650_DAPI_20x_3_DAPI 1.tif]

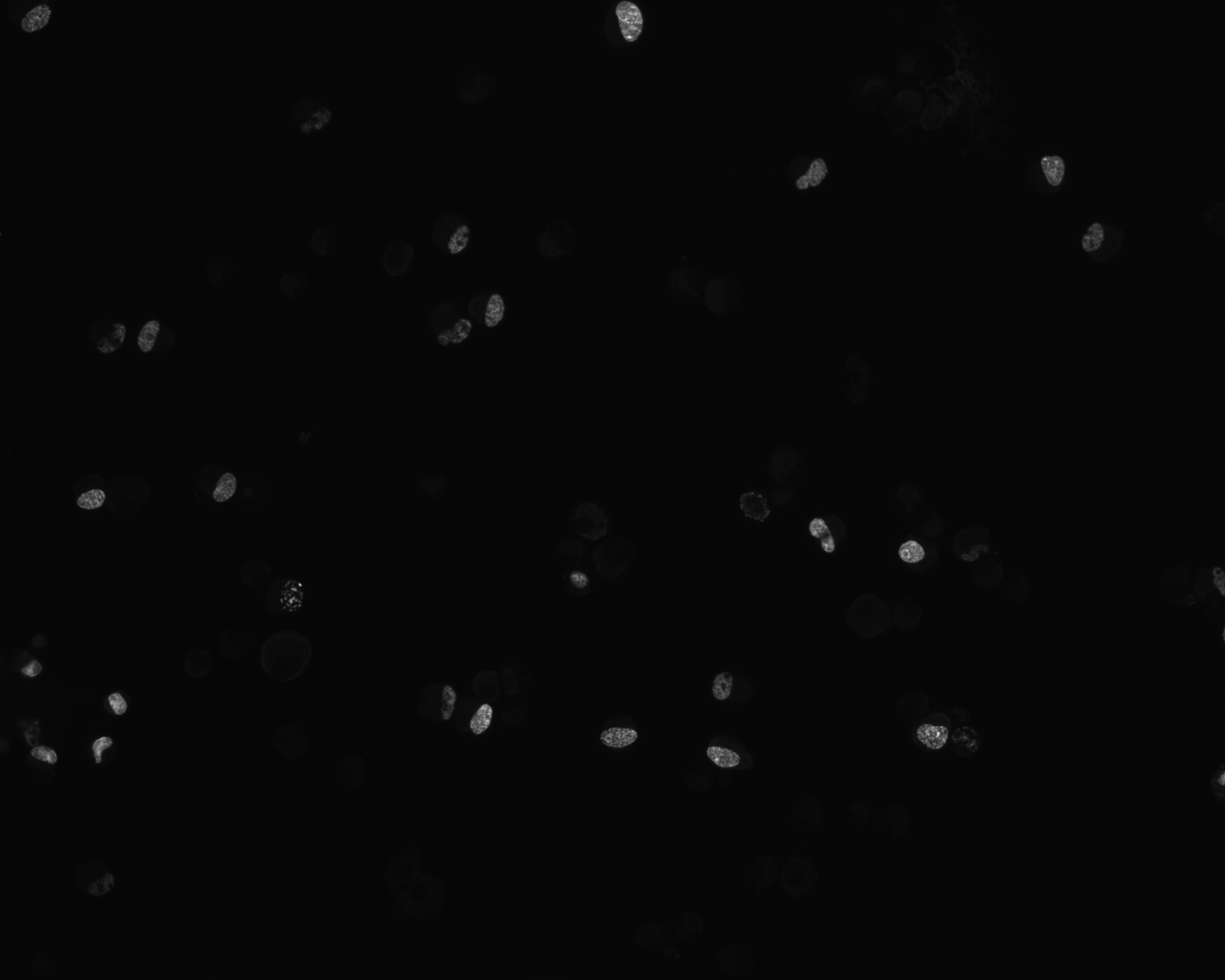

Supplement: S3 Data — (ZIP) [file pone.0233647.s006.zip › PACE Corrected/rSC51_SOX10AF488_EdUAF555_VIMEDL650_DAPI_20x_3_EdU 4.tif]

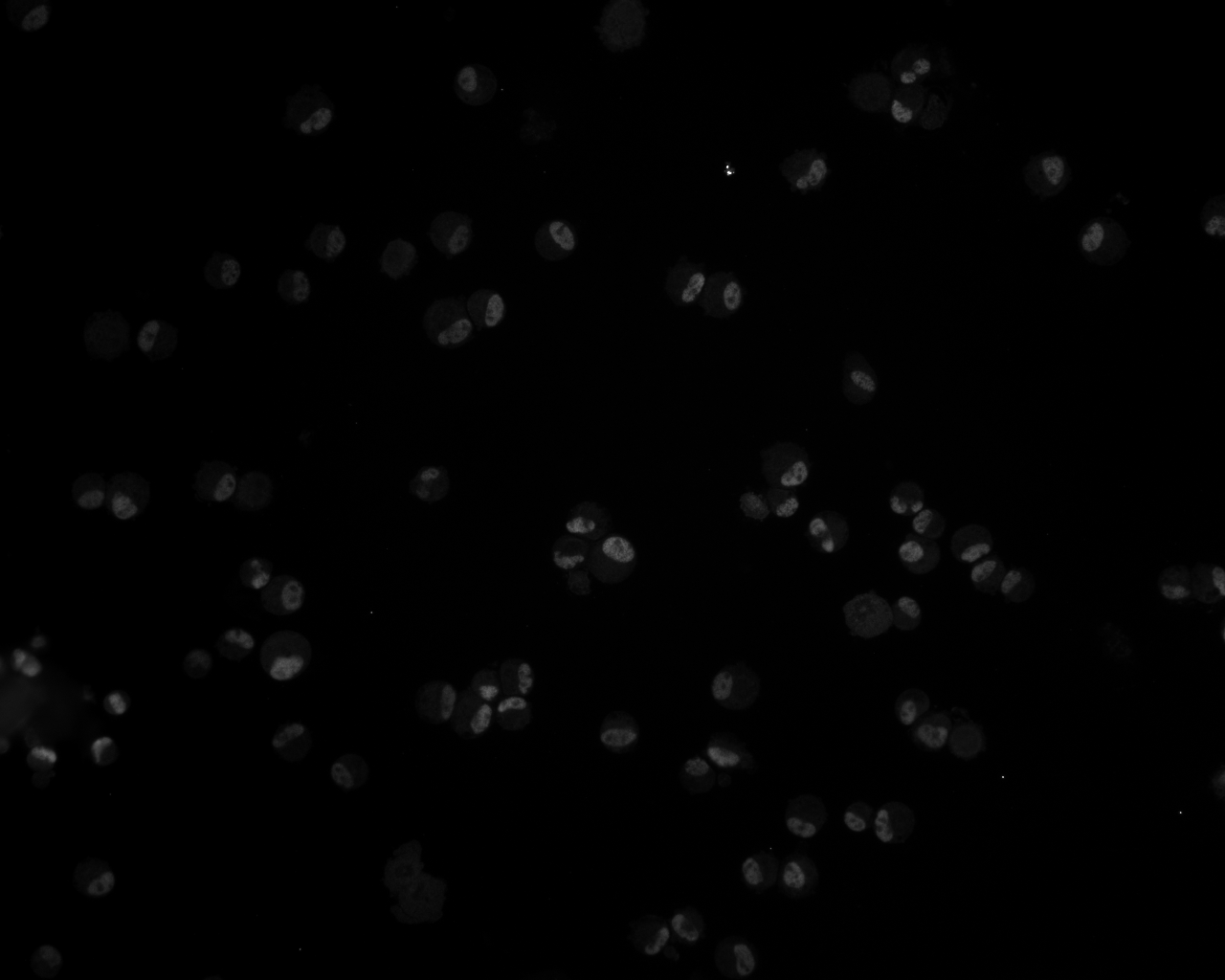

Supplement: S3 Data — (ZIP) [file pone.0233647.s006.zip › PACE Corrected/rSC51_SOX10AF488_EdUAF555_VIMEDL650_DAPI_20x_3_SOX10 2.tif]

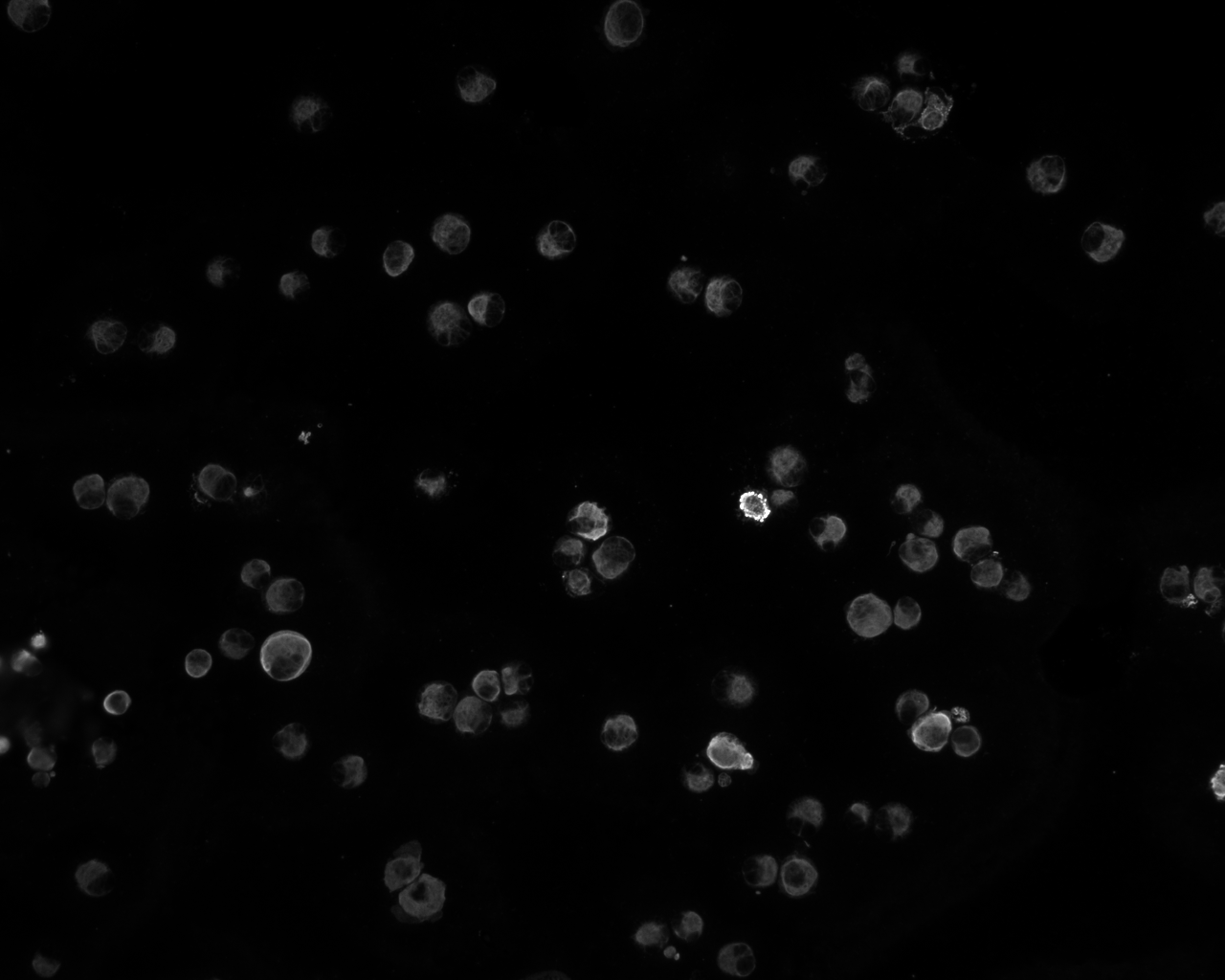

Supplement: S3 Data — (ZIP) [file pone.0233647.s006.zip › PACE Corrected/rSC51_SOX10AF488_EdUAF555_VIMEDL650_DAPI_20x_3_VIME 3.tif]

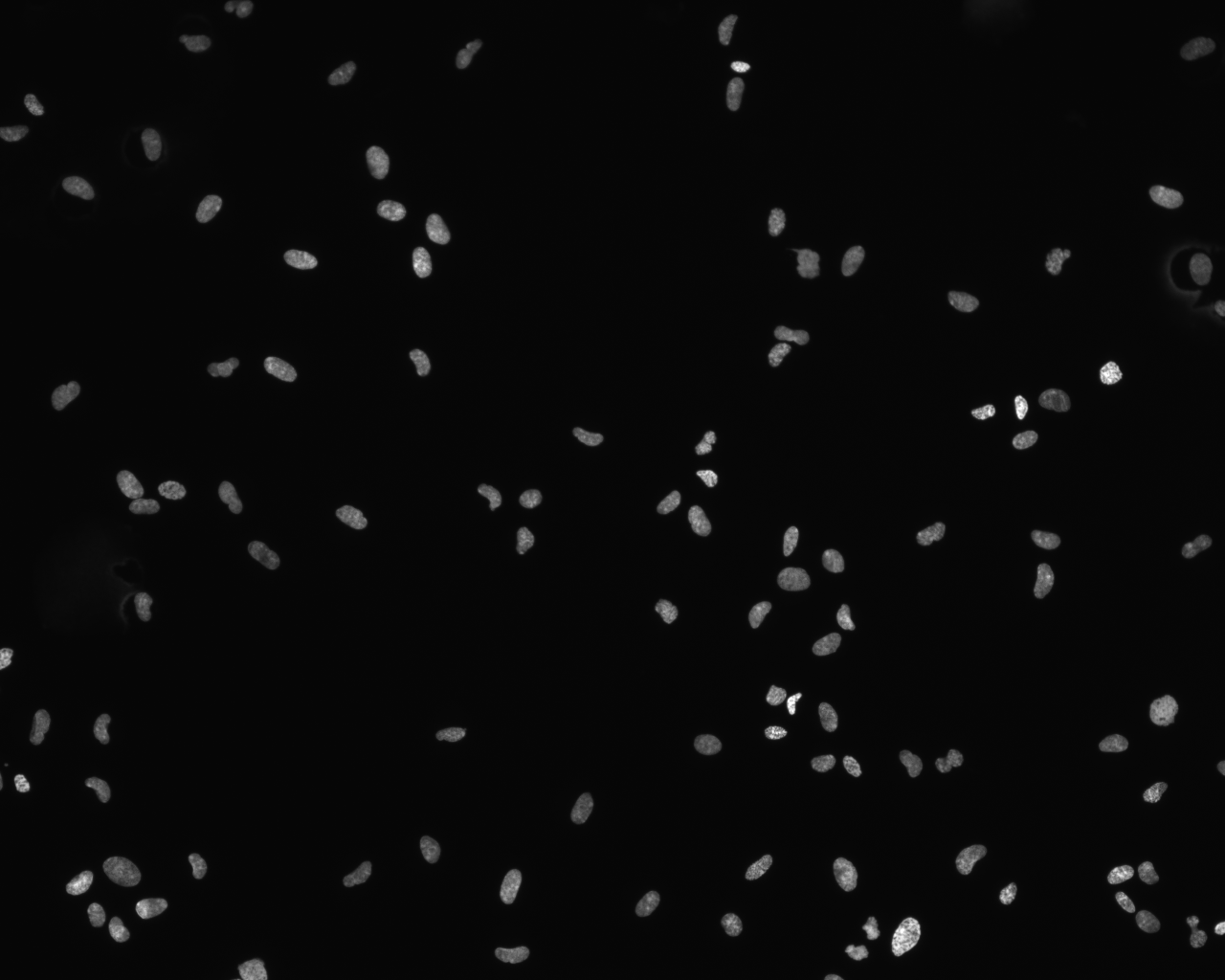

Supplement: S3 Data — (ZIP) [file pone.0233647.s006.zip › PACE Corrected/rSC51_SOX10AF488_EdUAF555_VIMEDL650_DAPI_20x_4_DAPI 1.tif]

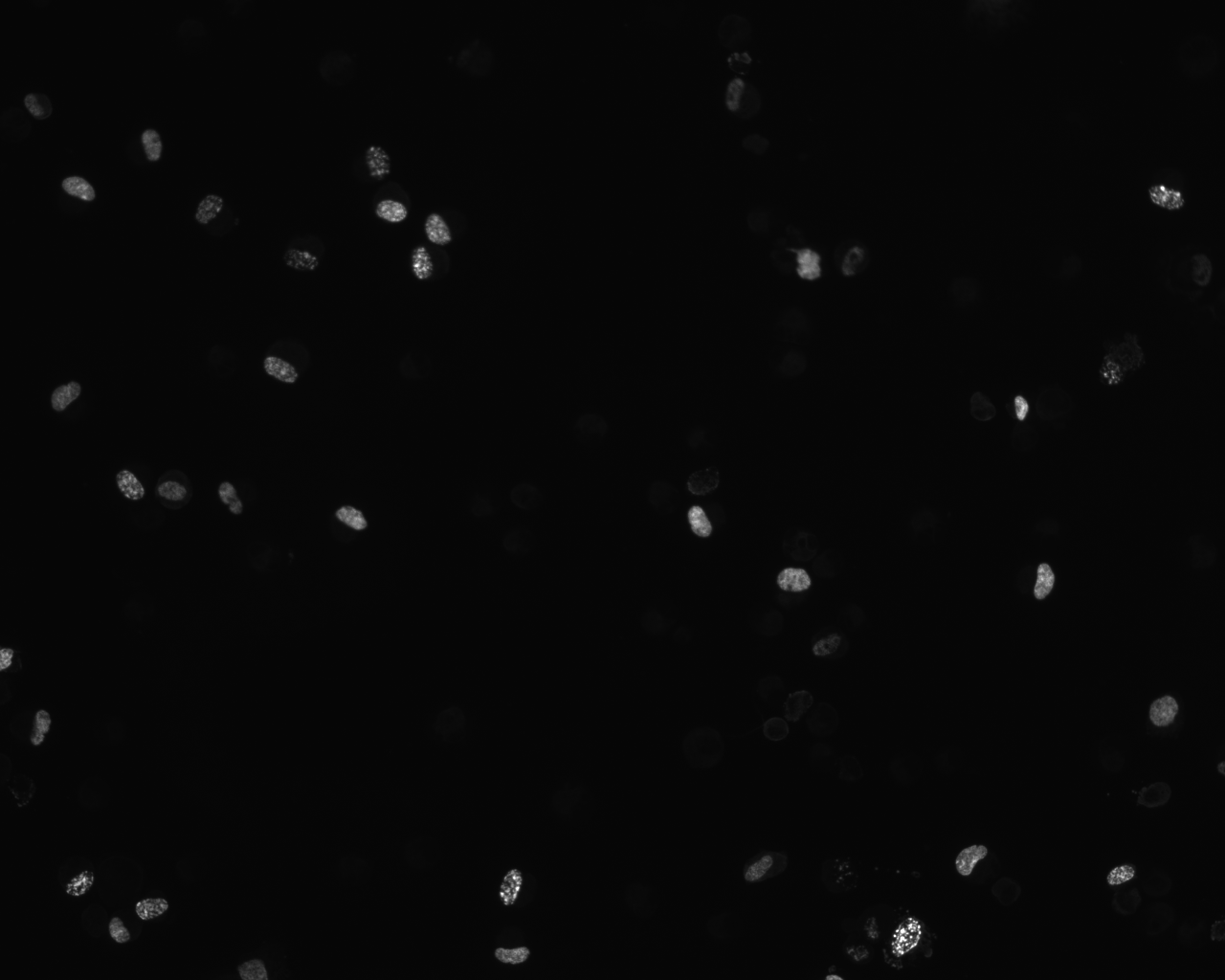

Supplement: S3 Data — (ZIP) [file pone.0233647.s006.zip › PACE Corrected/rSC51_SOX10AF488_EdUAF555_VIMEDL650_DAPI_20x_4_EdU 4.tif]

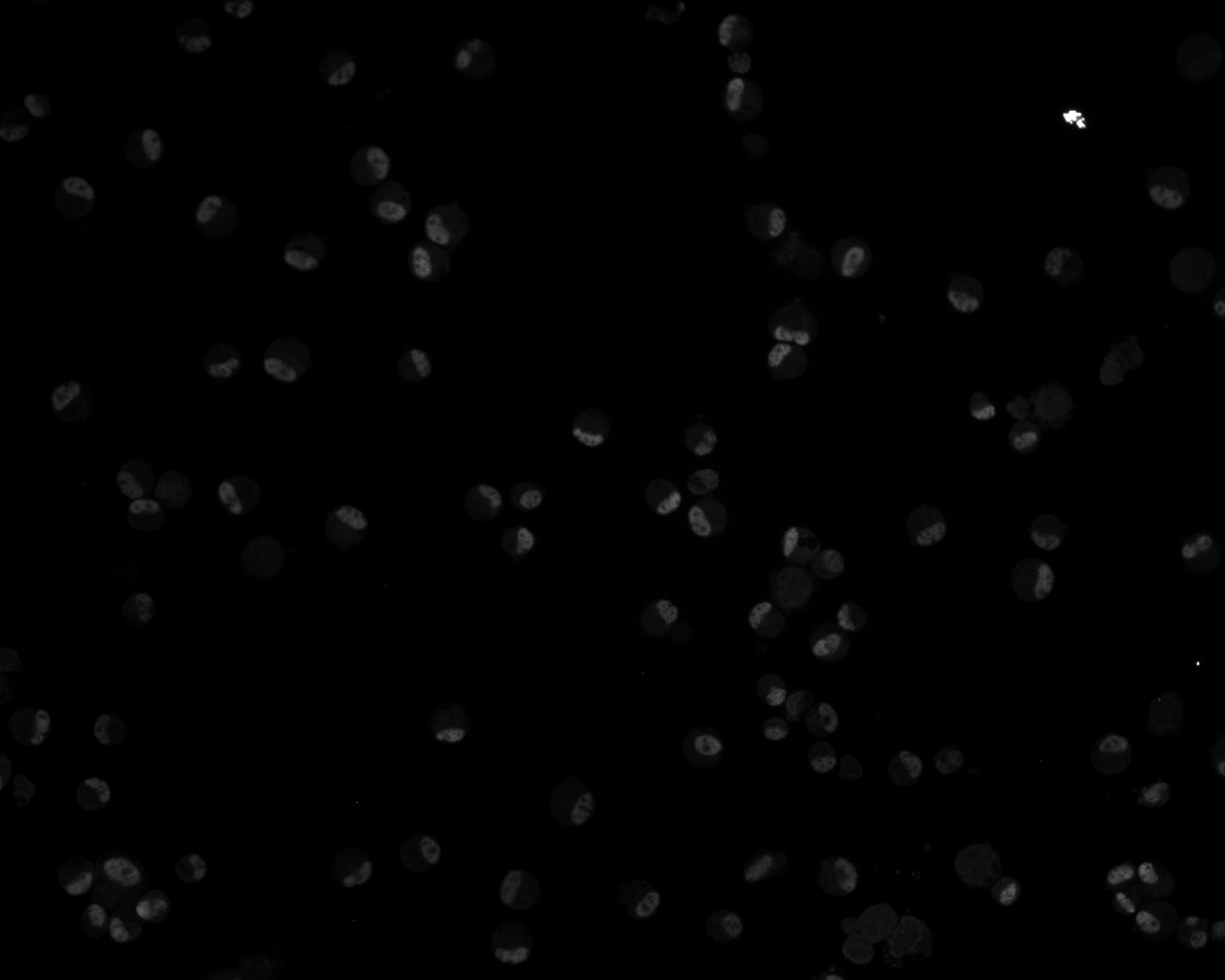

Supplement: S3 Data — (ZIP) [file pone.0233647.s006.zip › PACE Corrected/rSC51_SOX10AF488_EdUAF555_VIMEDL650_DAPI_20x_4_SOX10 2.tif]

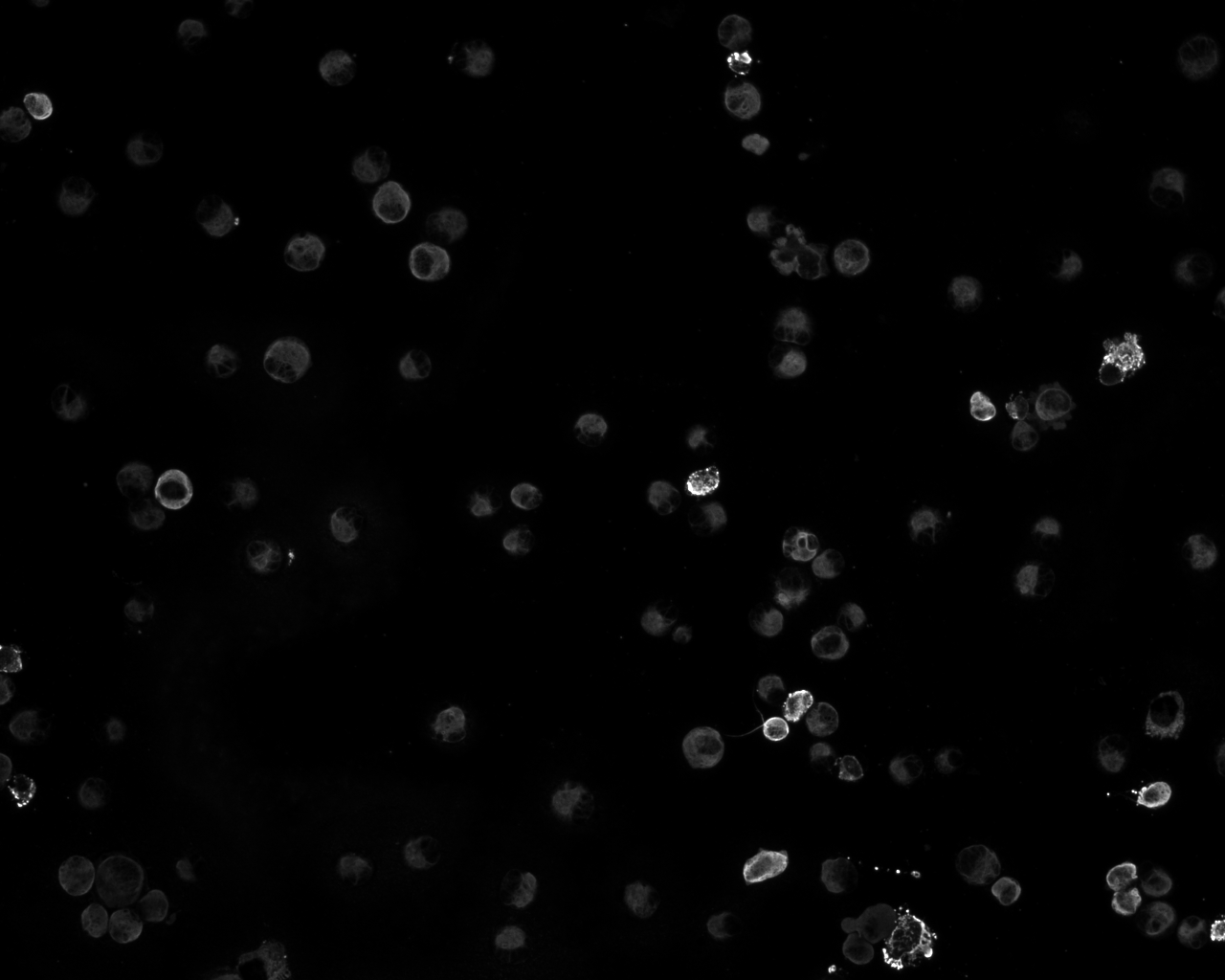

Supplement: S3 Data — (ZIP) [file pone.0233647.s006.zip › PACE Corrected/rSC51_SOX10AF488_EdUAF555_VIMEDL650_DAPI_20x_4_VIME 3.tif]

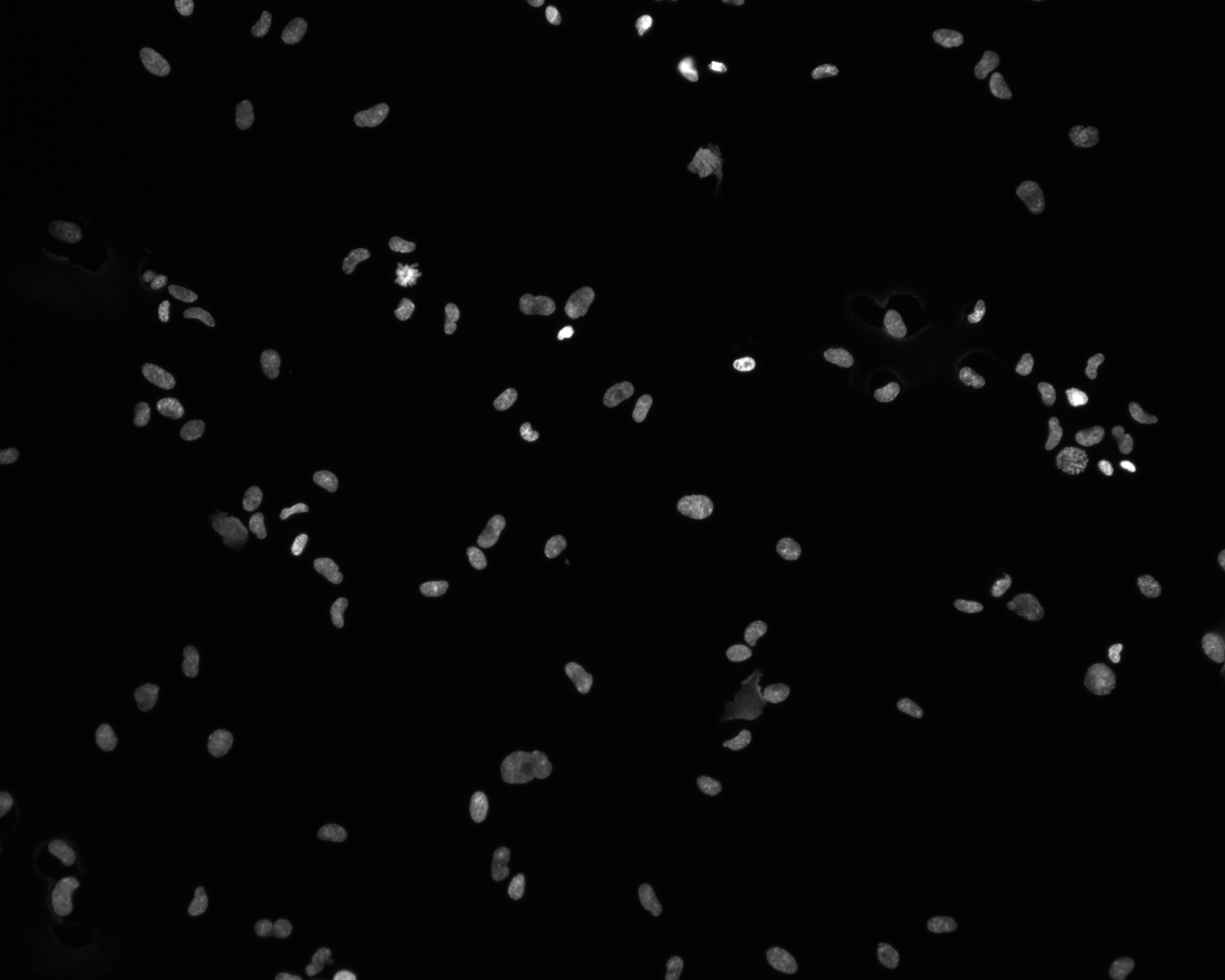

Supplement: S3 Data — (ZIP) [file pone.0233647.s006.zip › PACE Corrected/rSC51_SOX10AF488_EdUAF555_VIMEDL650_DAPI_20x_5_DAPI 1.tif]

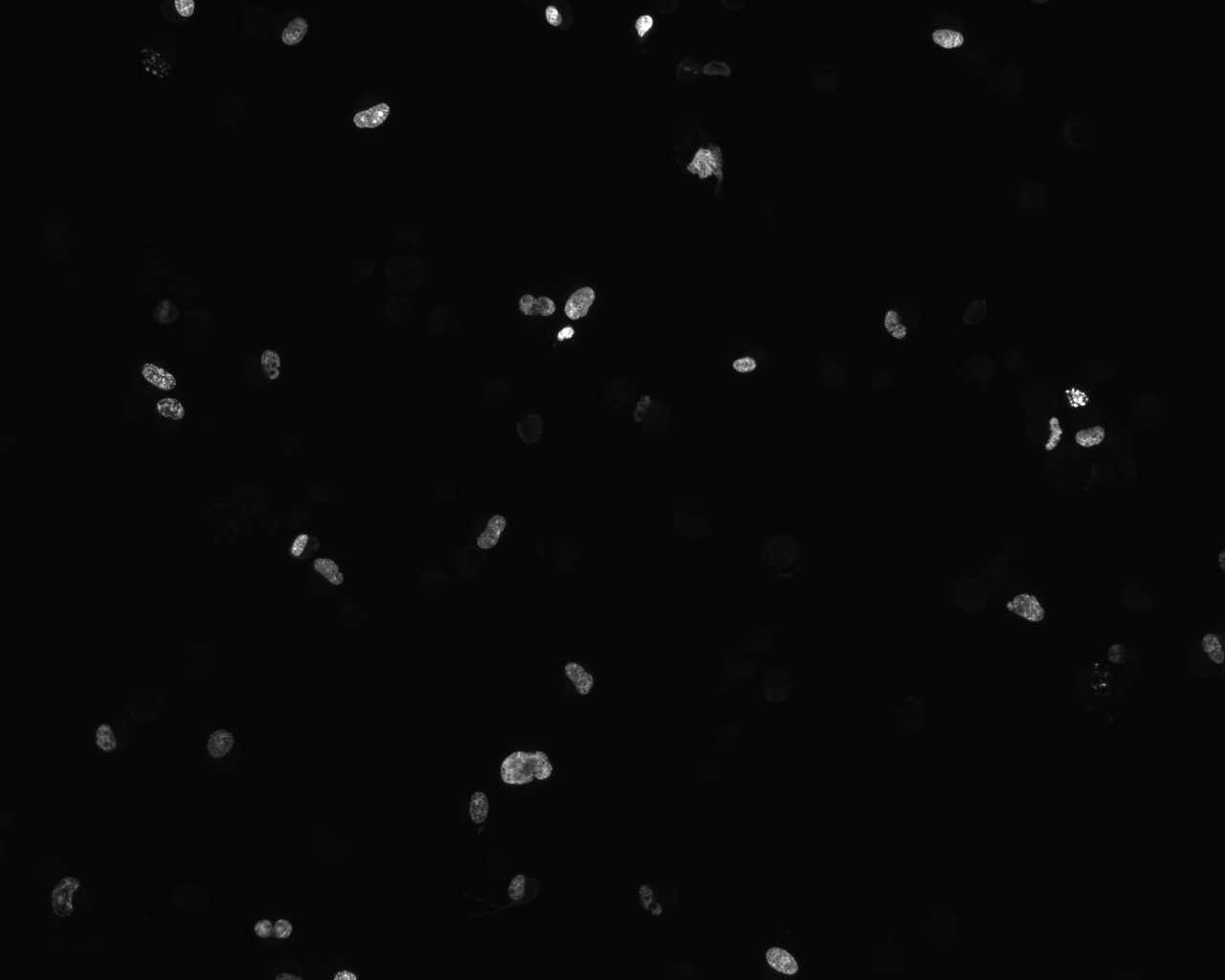

Supplement: S3 Data — (ZIP) [file pone.0233647.s006.zip › PACE Corrected/rSC51_SOX10AF488_EdUAF555_VIMEDL650_DAPI_20x_5_EdU 4.tif]

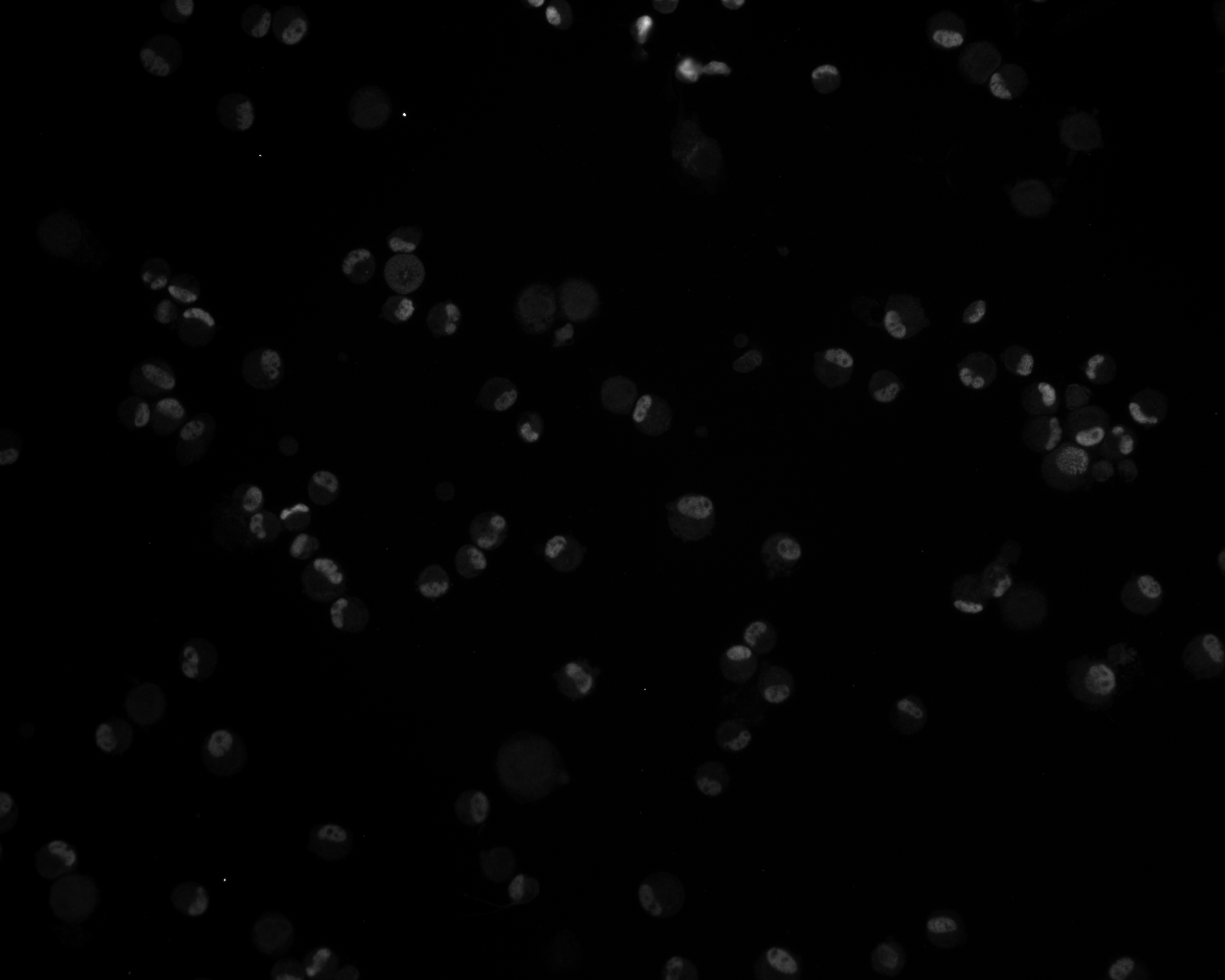

Supplement: S3 Data — (ZIP) [file pone.0233647.s006.zip › PACE Corrected/rSC51_SOX10AF488_EdUAF555_VIMEDL650_DAPI_20x_5_SOX10 2 2.tif]

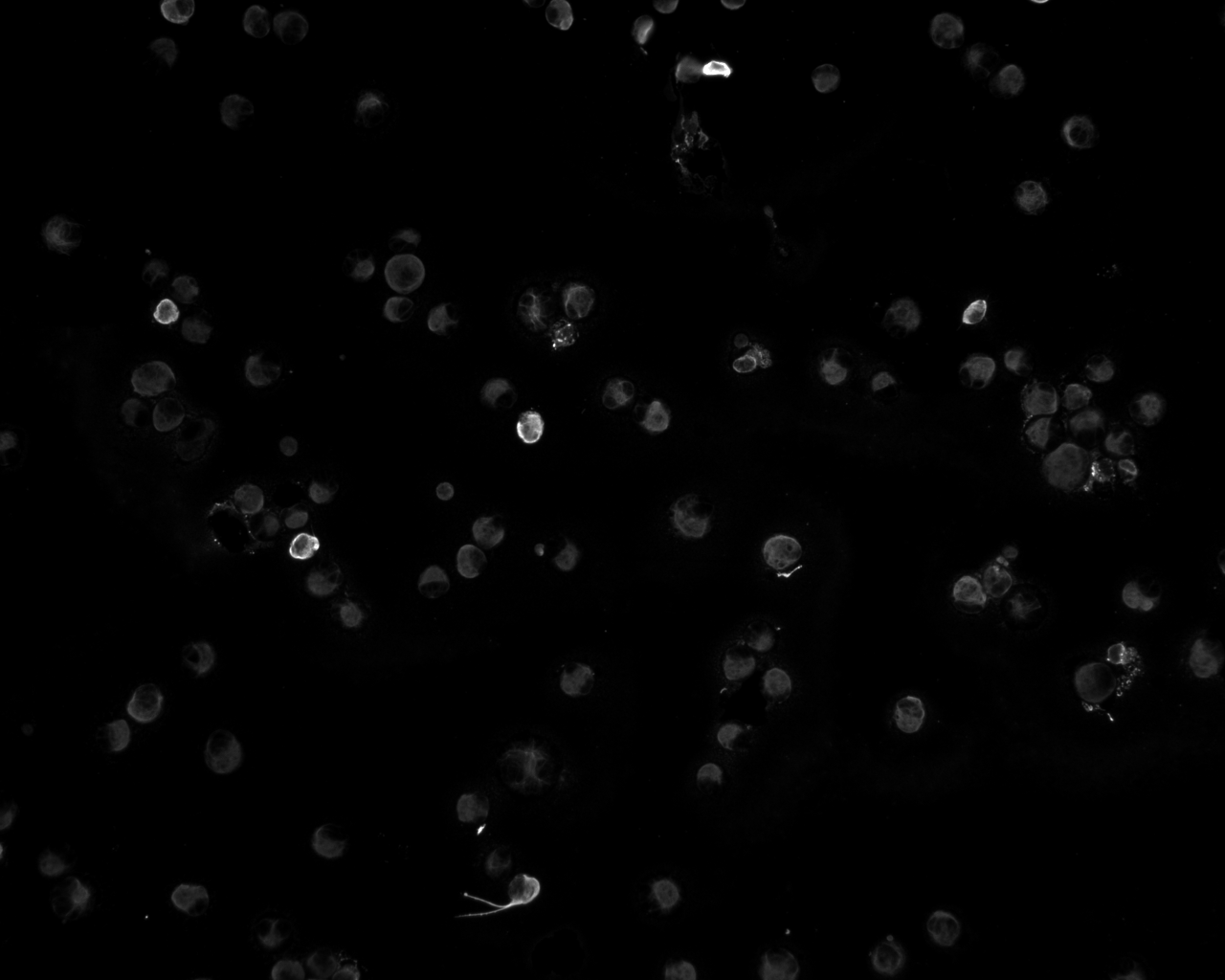

Supplement: S3 Data — (ZIP) [file pone.0233647.s006.zip › PACE Corrected/rSC51_SOX10AF488_EdUAF555_VIMEDL650_DAPI_20x_5_VIME 3.tif]

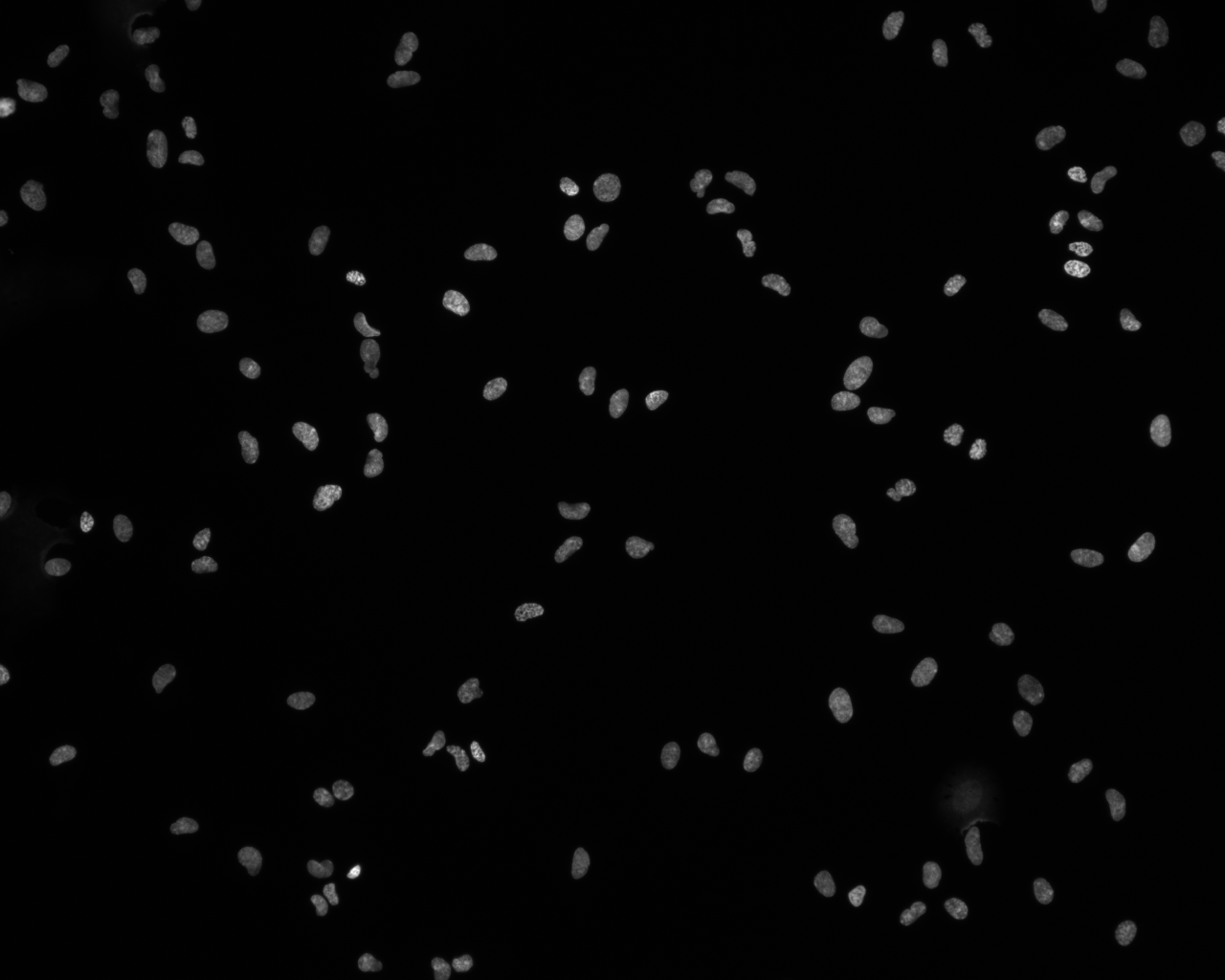

Supplement: S3 Data — (ZIP) [file pone.0233647.s006.zip › PACE Corrected/rSC51_SOX10AF488_EdUAF555_VIMEDL650_DAPI_20x_6_DAPI 1.tif]

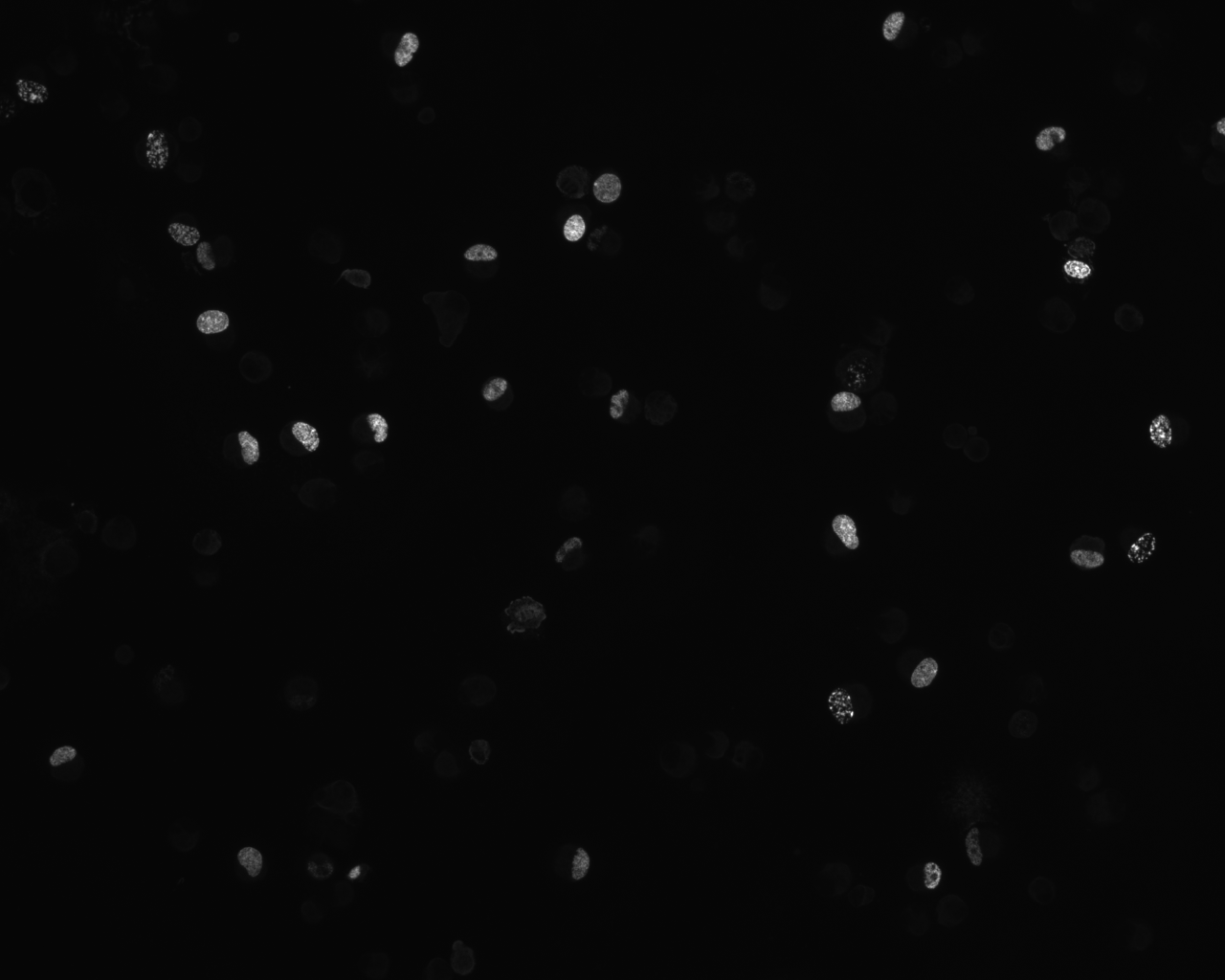

Supplement: S3 Data — (ZIP) [file pone.0233647.s006.zip › PACE Corrected/rSC51_SOX10AF488_EdUAF555_VIMEDL650_DAPI_20x_6_EdU 4.tif]

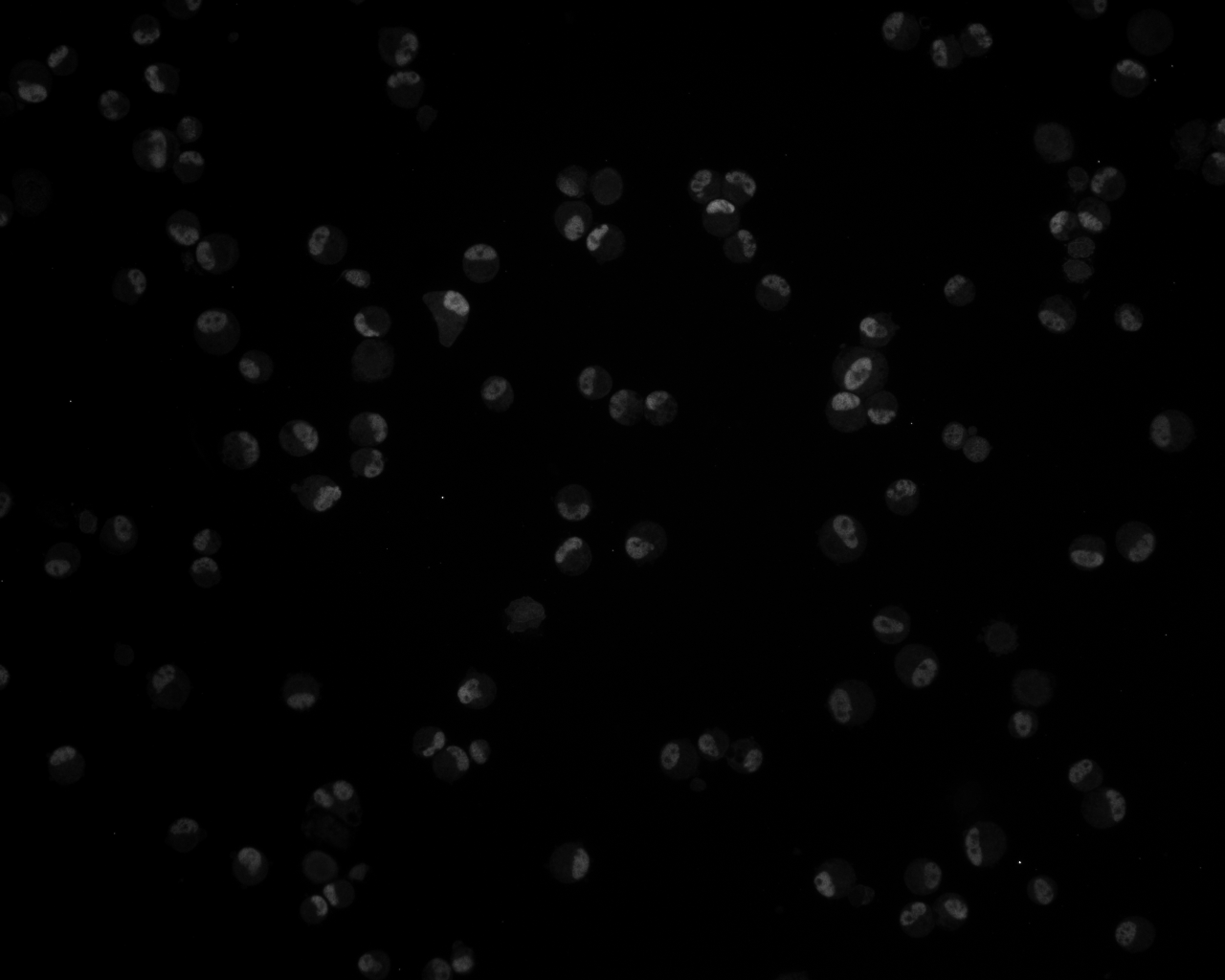

Supplement: S3 Data — (ZIP) [file pone.0233647.s006.zip › PACE Corrected/rSC51_SOX10AF488_EdUAF555_VIMEDL650_DAPI_20x_6_SOX10 2.tif]

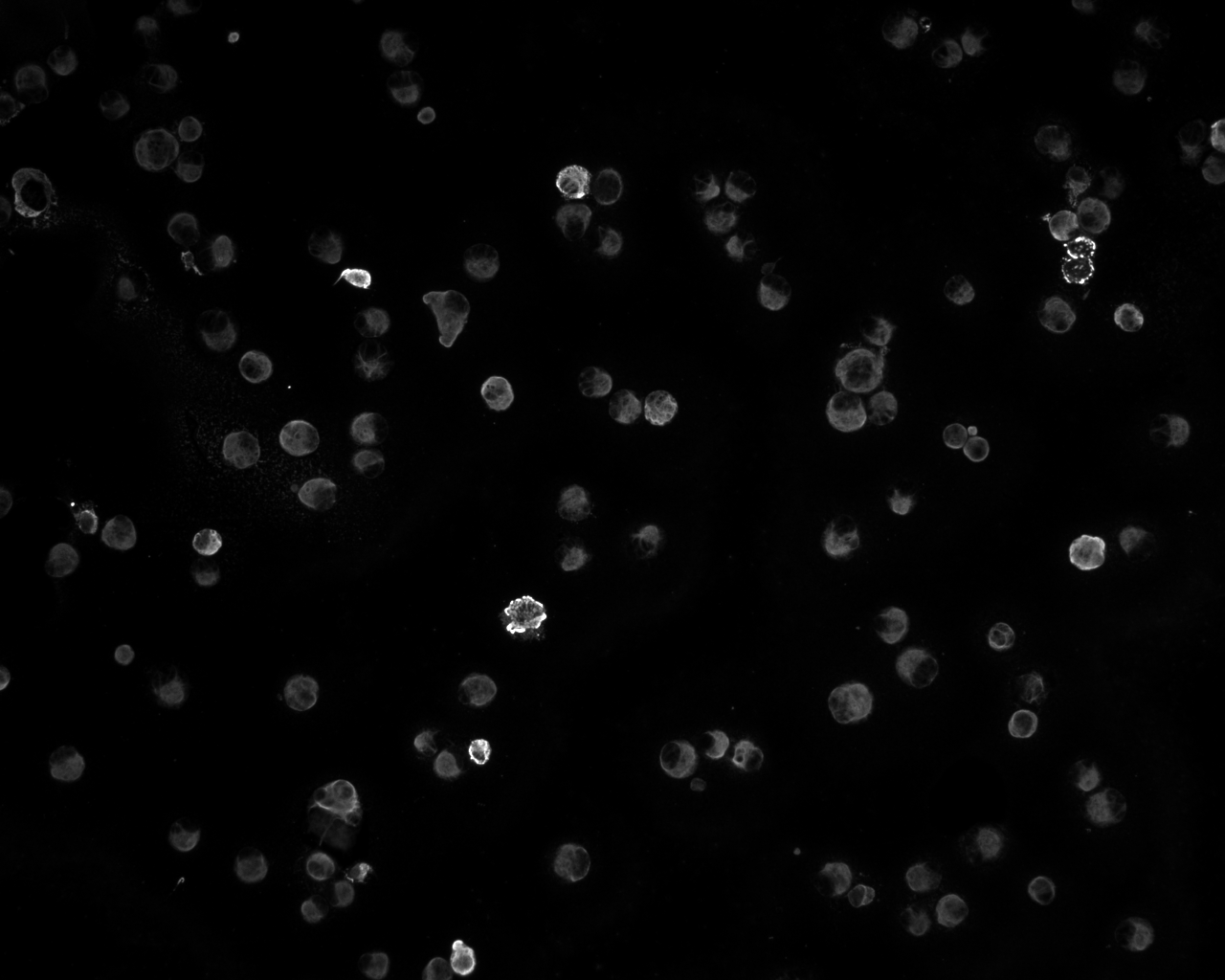

Supplement: S3 Data — (ZIP) [file pone.0233647.s006.zip › PACE Corrected/rSC51_SOX10AF488_EdUAF555_VIMEDL650_DAPI_20x_6_VIME 3.tif]

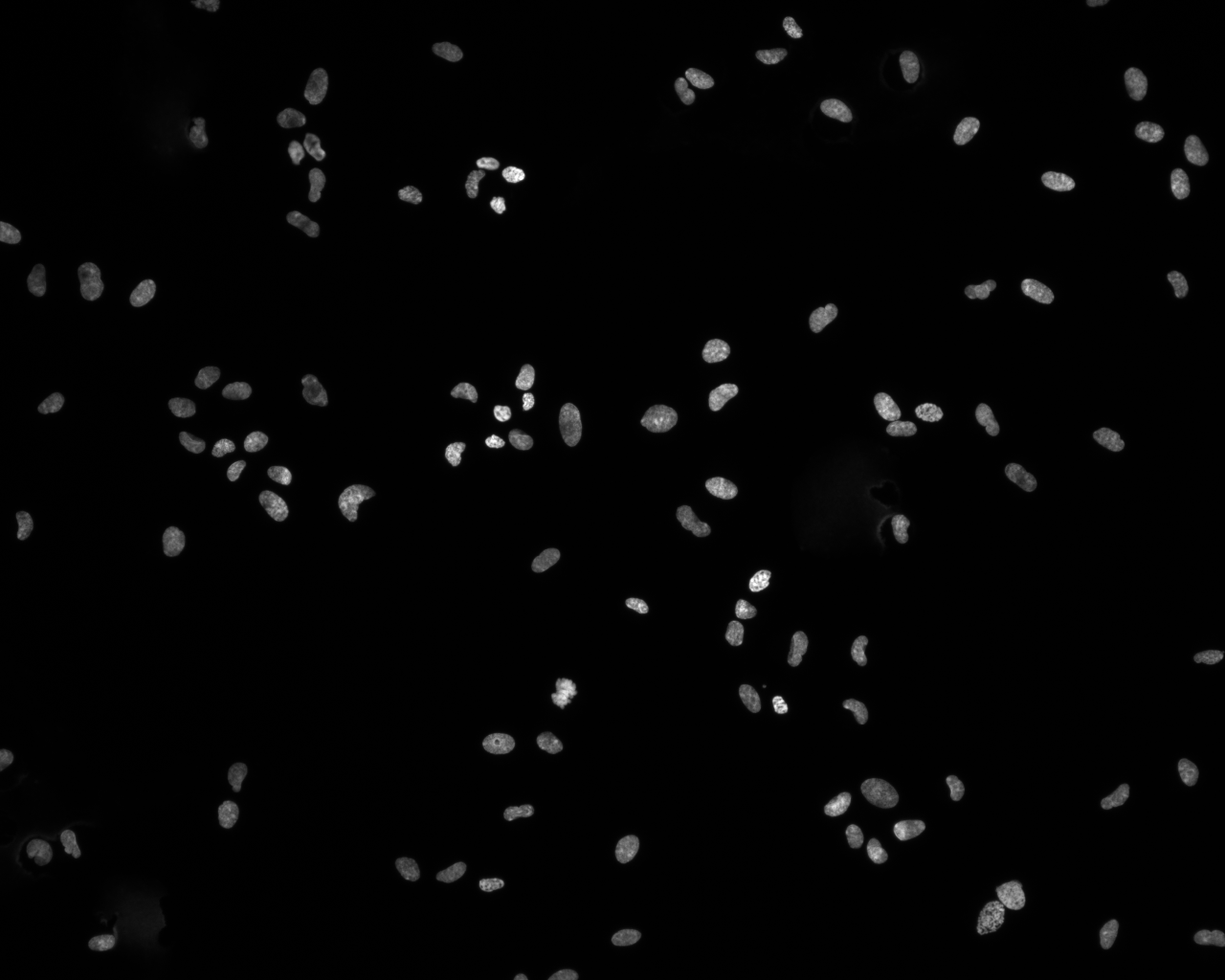

Supplement: S3 Data — (ZIP) [file pone.0233647.s006.zip › PACE Corrected/rSC51_SOX10AF488_EdUAF555_VIMEDL650_DAPI_20x_8_DAPI 1.tif]

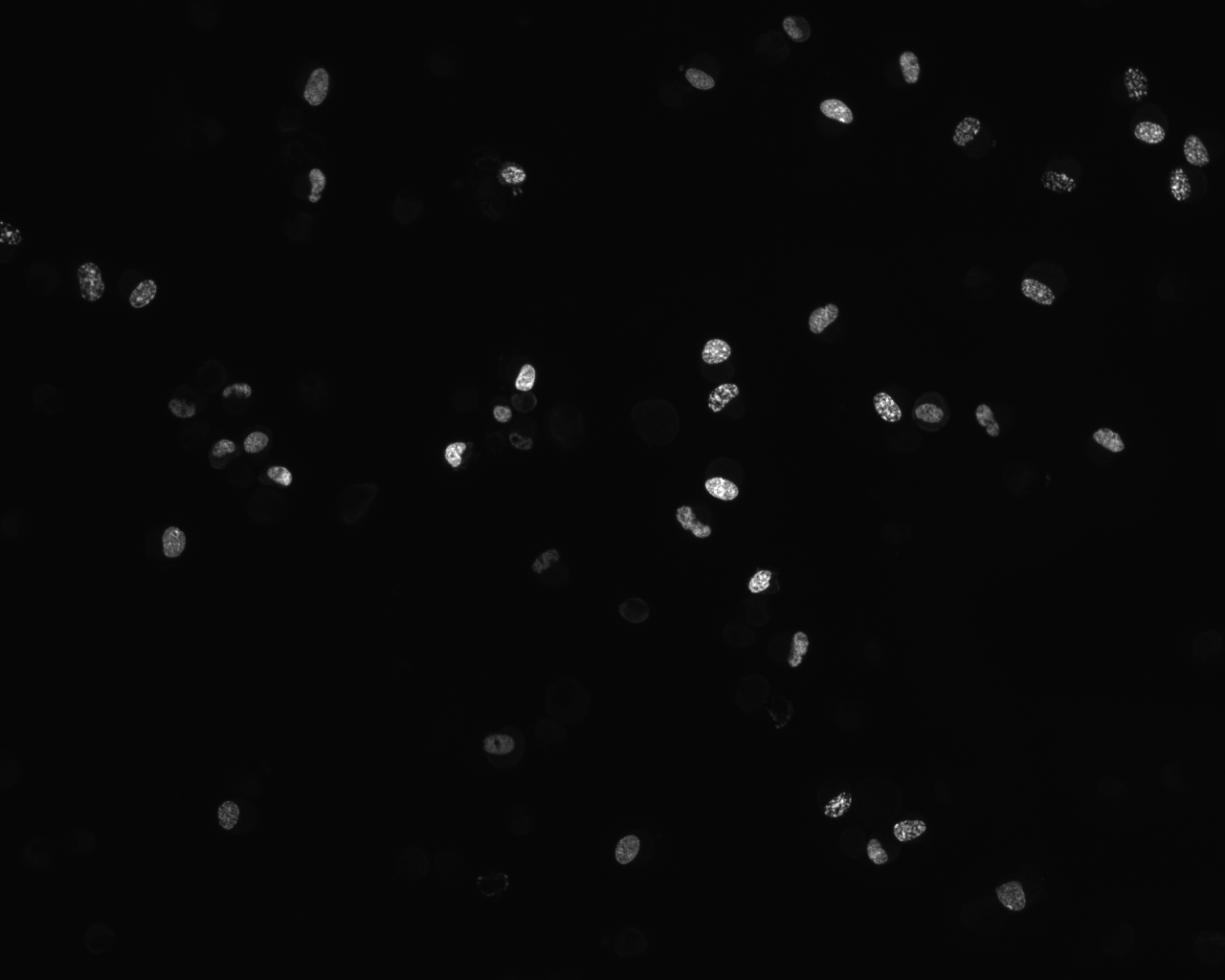

Supplement: S3 Data — (ZIP) [file pone.0233647.s006.zip › PACE Corrected/rSC51_SOX10AF488_EdUAF555_VIMEDL650_DAPI_20x_8_EdU 4.tif]

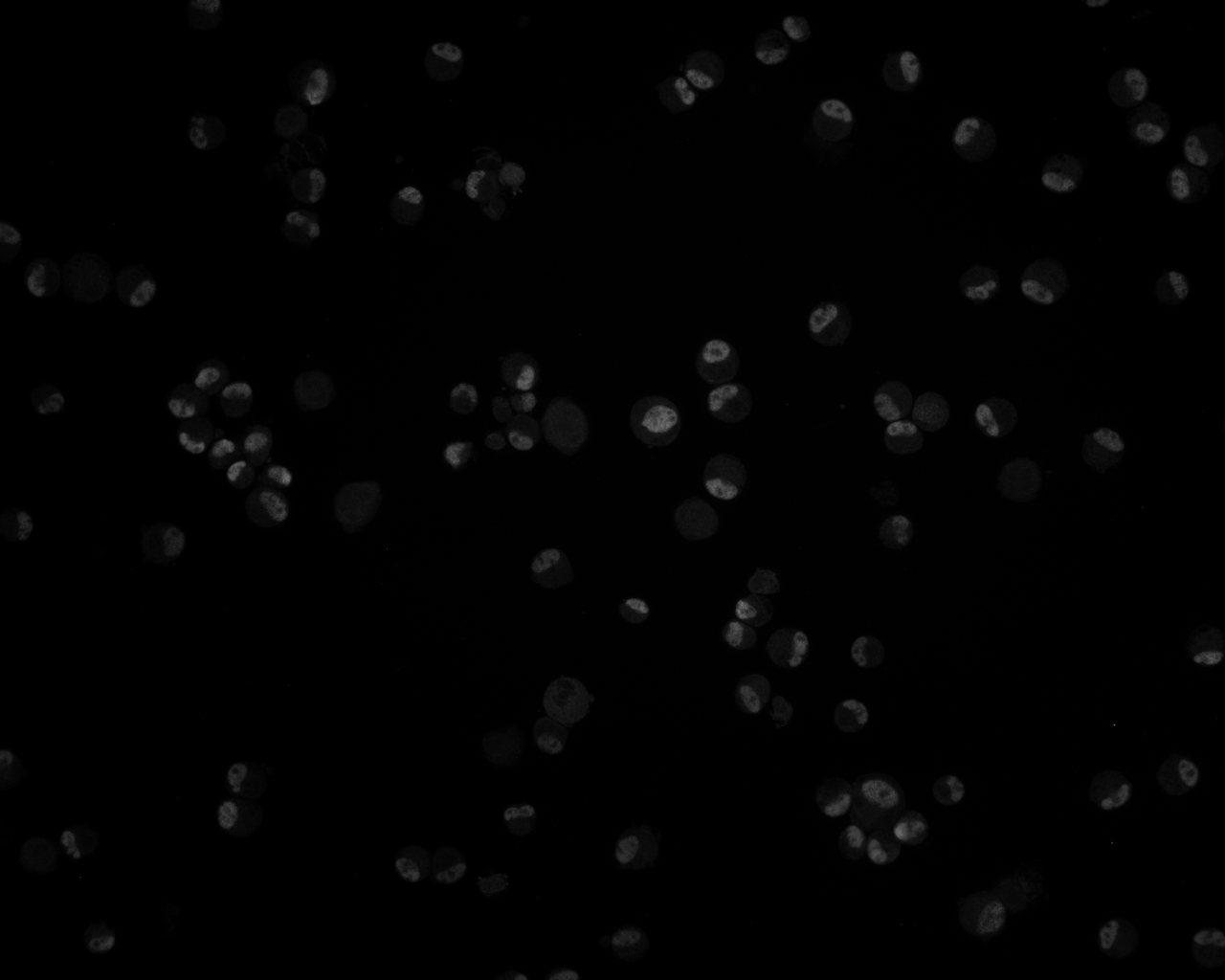

Supplement: S3 Data — (ZIP) [file pone.0233647.s006.zip › PACE Corrected/rSC51_SOX10AF488_EdUAF555_VIMEDL650_DAPI_20x_8_SOX10 2.tif]

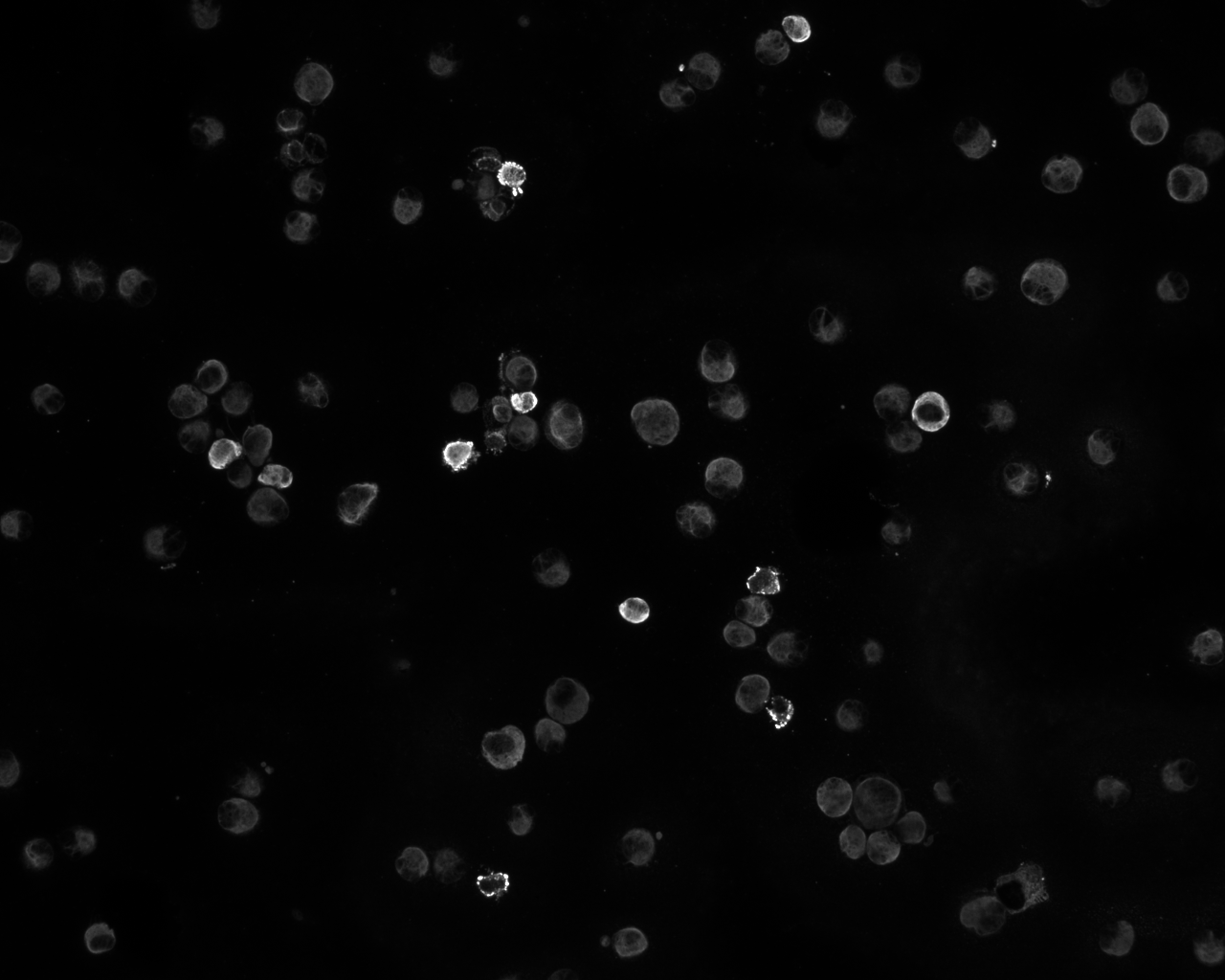

Supplement: S3 Data — (ZIP) [file pone.0233647.s006.zip › PACE Corrected/rSC51_SOX10AF488_EdUAF555_VIMEDL650_DAPI_20x_8_VIME 3.tif]

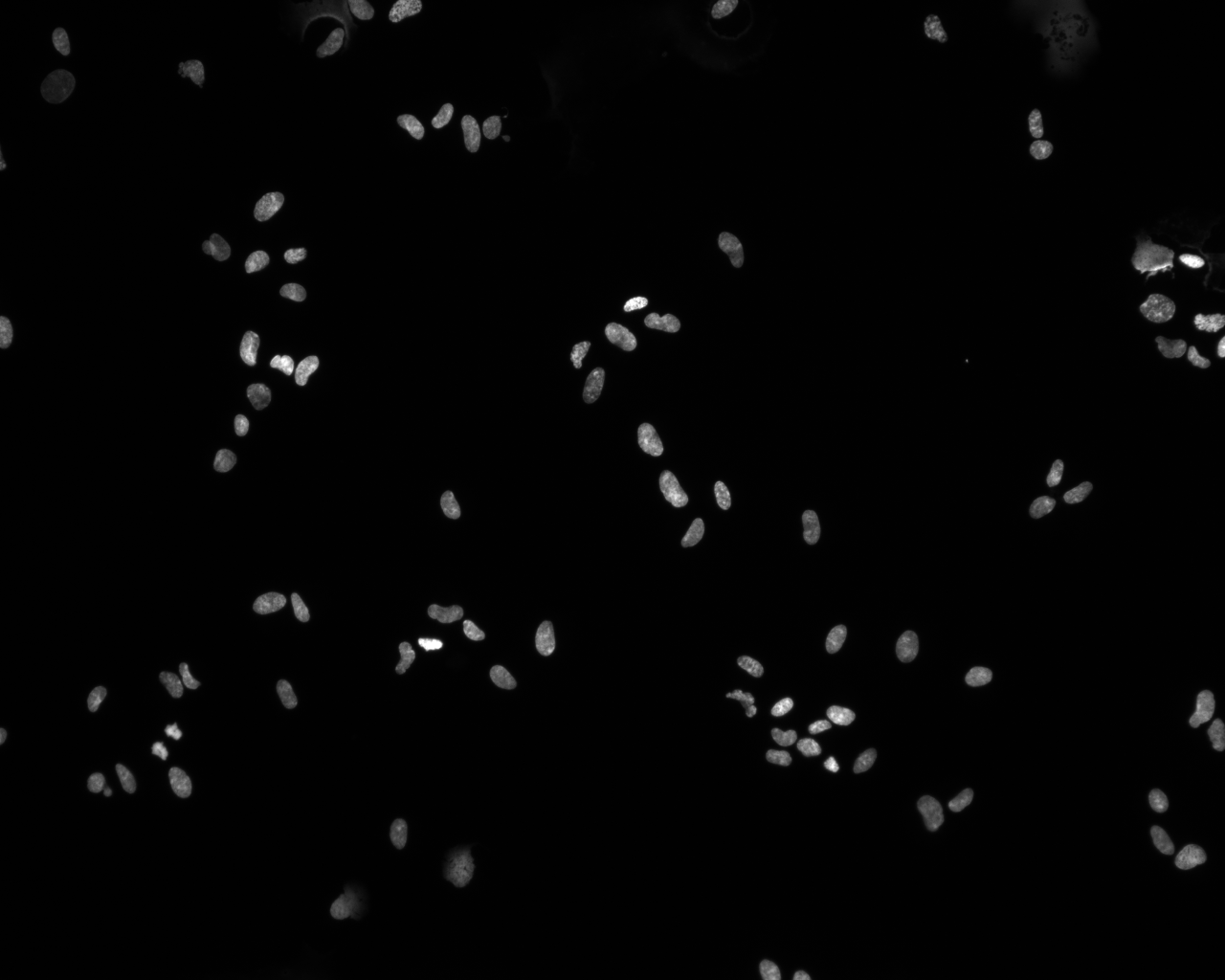

Supplement: S3 Data — (ZIP) [file pone.0233647.s006.zip › PACE Corrected/rSC51_SOX10AF488_EdUAF555_VIMEDL650_DAPI_20x_1_DAPI 1.tif]

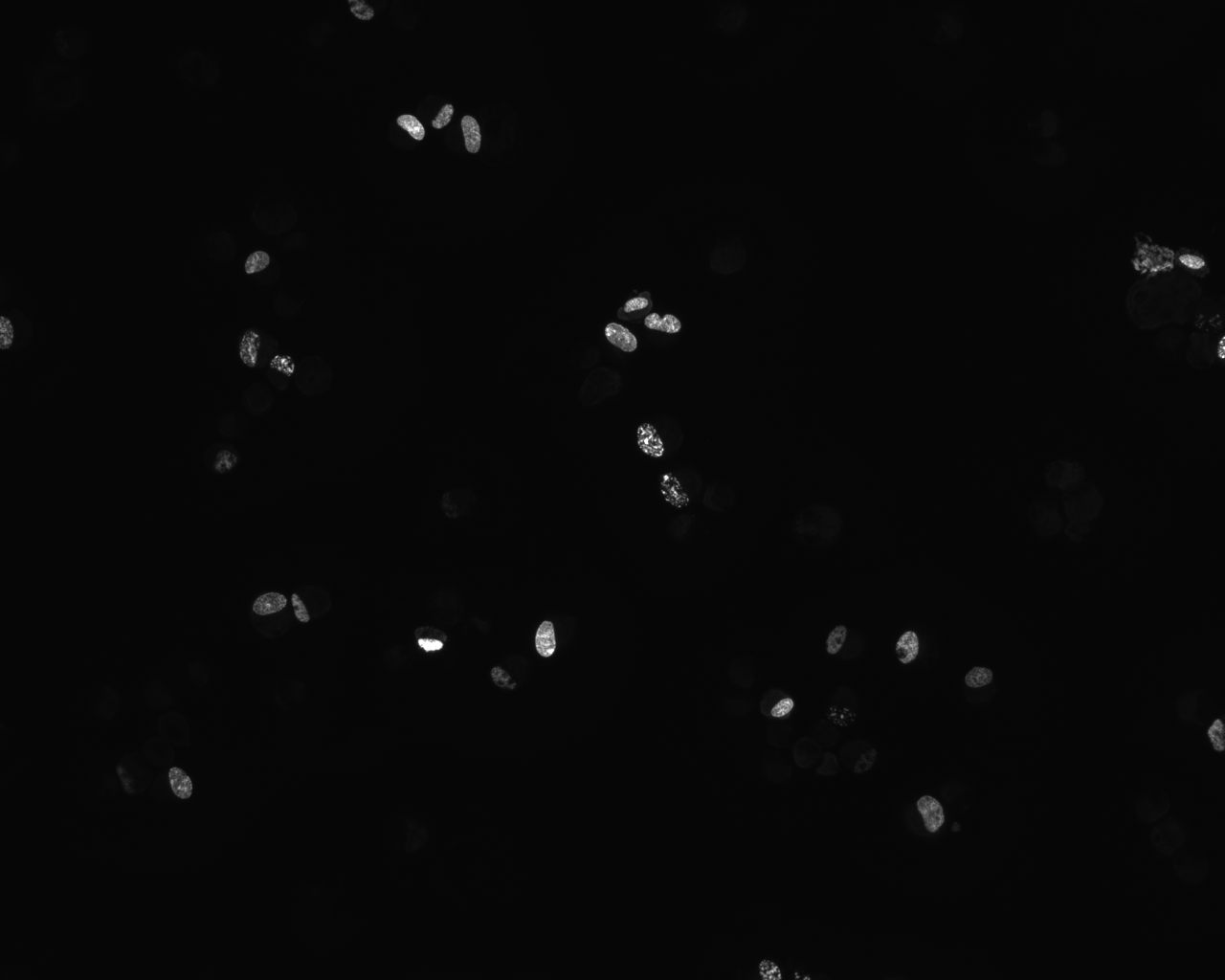

Supplement: S3 Data — (ZIP) [file pone.0233647.s006.zip › PACE Corrected/rSC51_SOX10AF488_EdUAF555_VIMEDL650_DAPI_20x_1_EdU 4.tif]

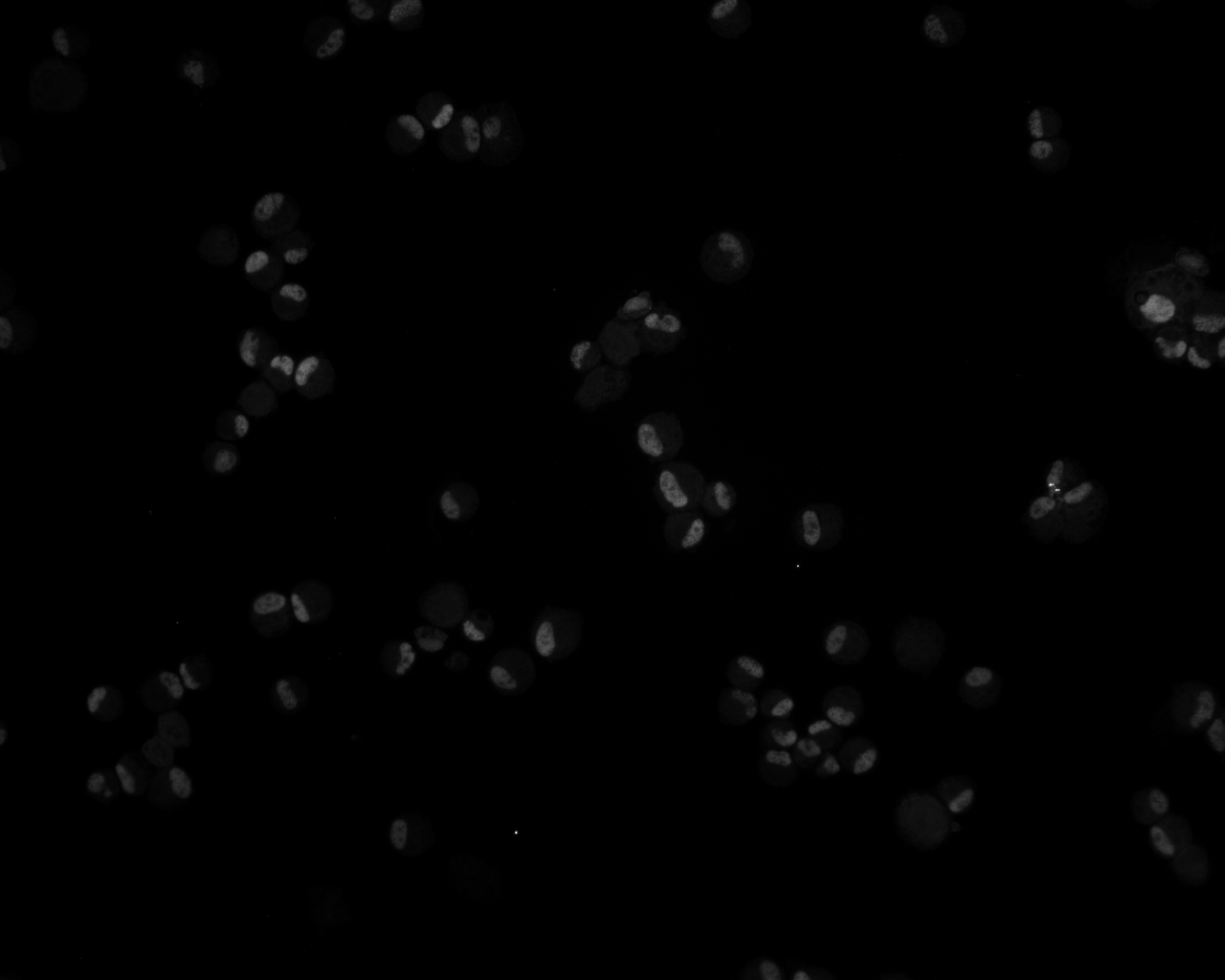

Supplement: S3 Data — (ZIP) [file pone.0233647.s006.zip › PACE Corrected/rSC51_SOX10AF488_EdUAF555_VIMEDL650_DAPI_20x_1_SOX10 2.tif]

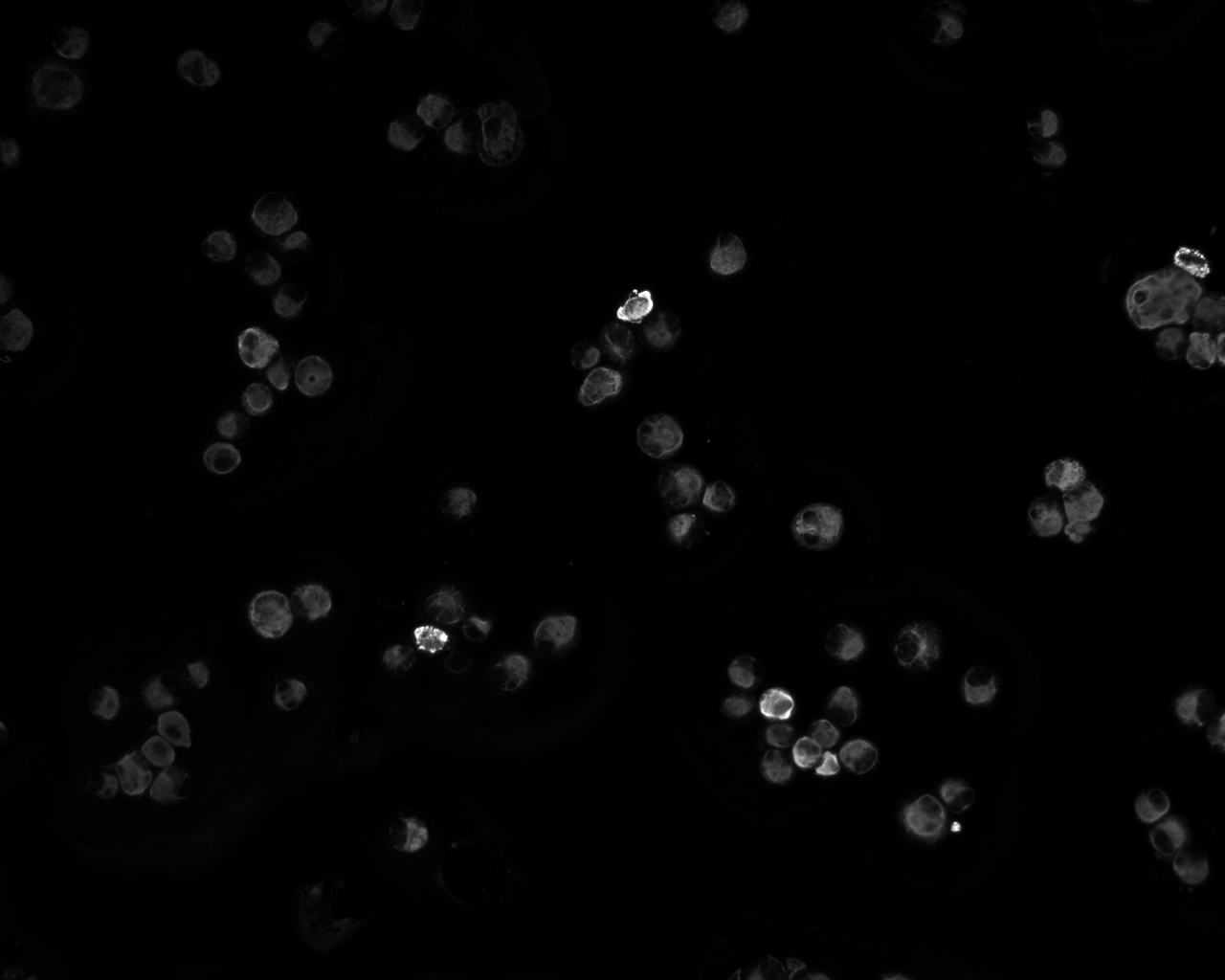

Supplement: S3 Data — (ZIP) [file pone.0233647.s006.zip › PACE Corrected/rSC51_SOX10AF488_EdUAF555_VIMEDL650_DAPI_20x_1_VIME 3.tif]

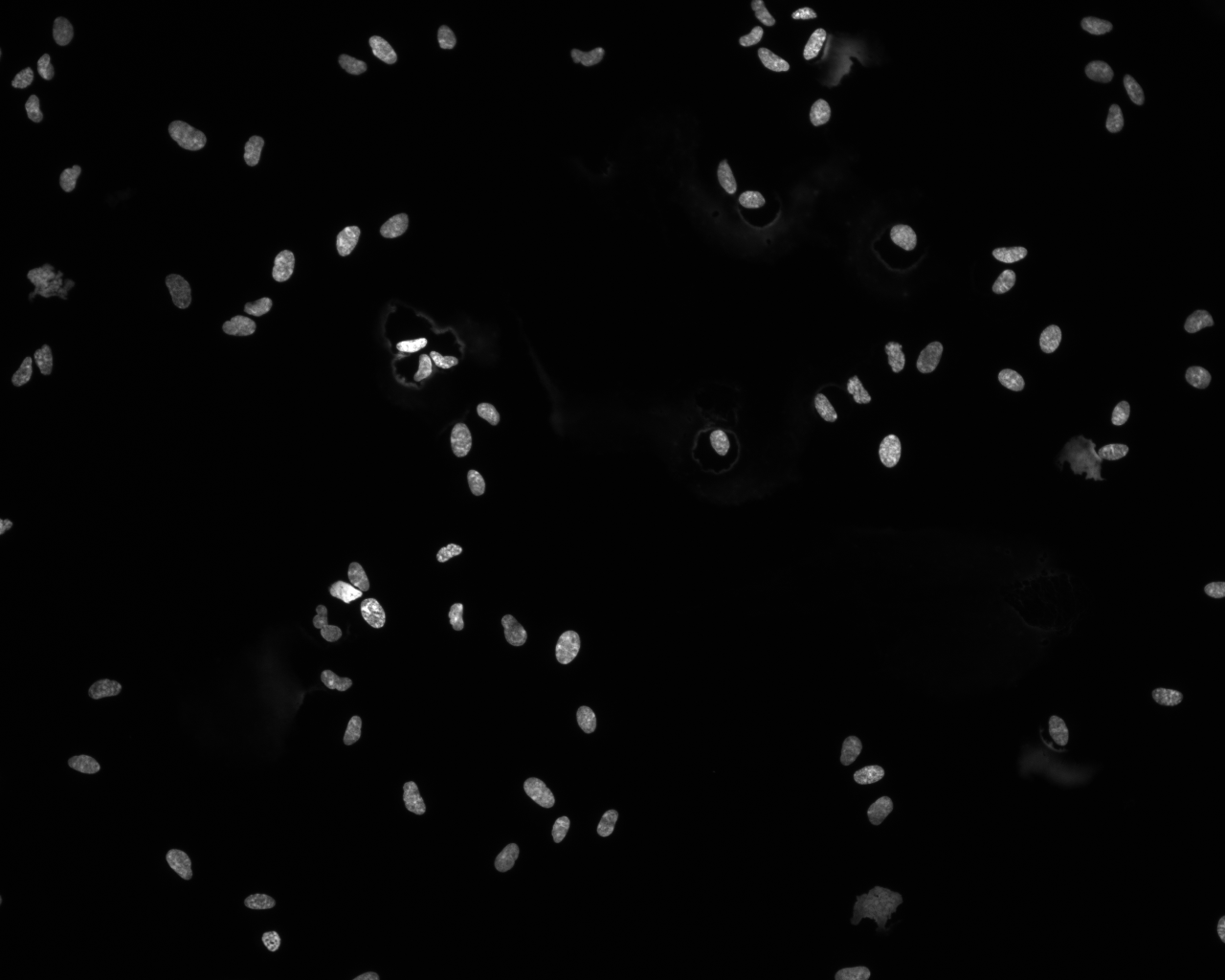

Supplement: S3 Data — (ZIP) [file pone.0233647.s006.zip › PACE Corrected/rSC51_SOX10AF488_EdUAF555_VIMEDL650_DAPI_20x_2_DAPI 1.tif]

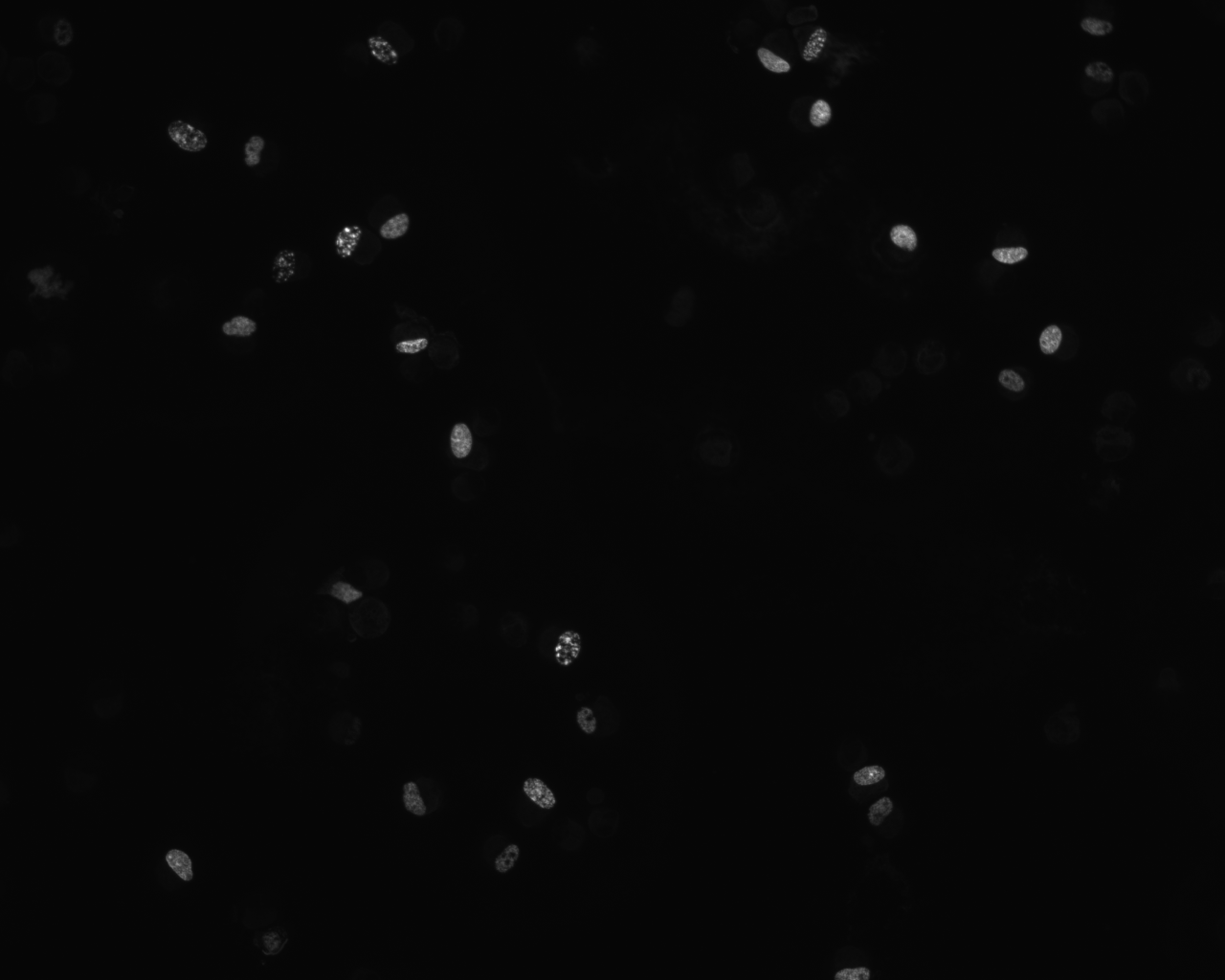

Supplement: S3 Data — (ZIP) [file pone.0233647.s006.zip › PACE Corrected/rSC51_SOX10AF488_EdUAF555_VIMEDL650_DAPI_20x_2_EdU 4.tif]

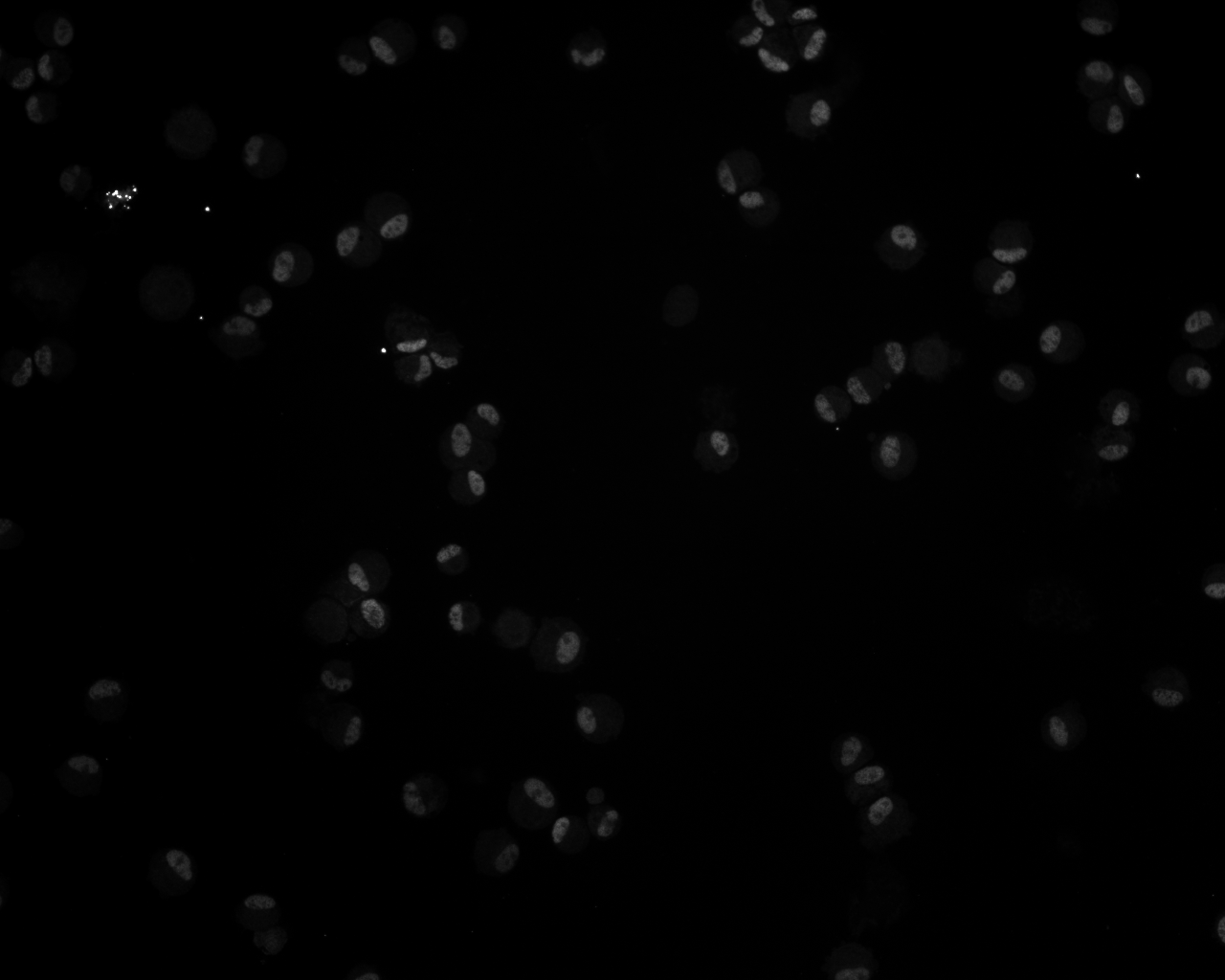

Supplement: S3 Data — (ZIP) [file pone.0233647.s006.zip › PACE Corrected/rSC51_SOX10AF488_EdUAF555_VIMEDL650_DAPI_20x_2_SOX10 2.tif]

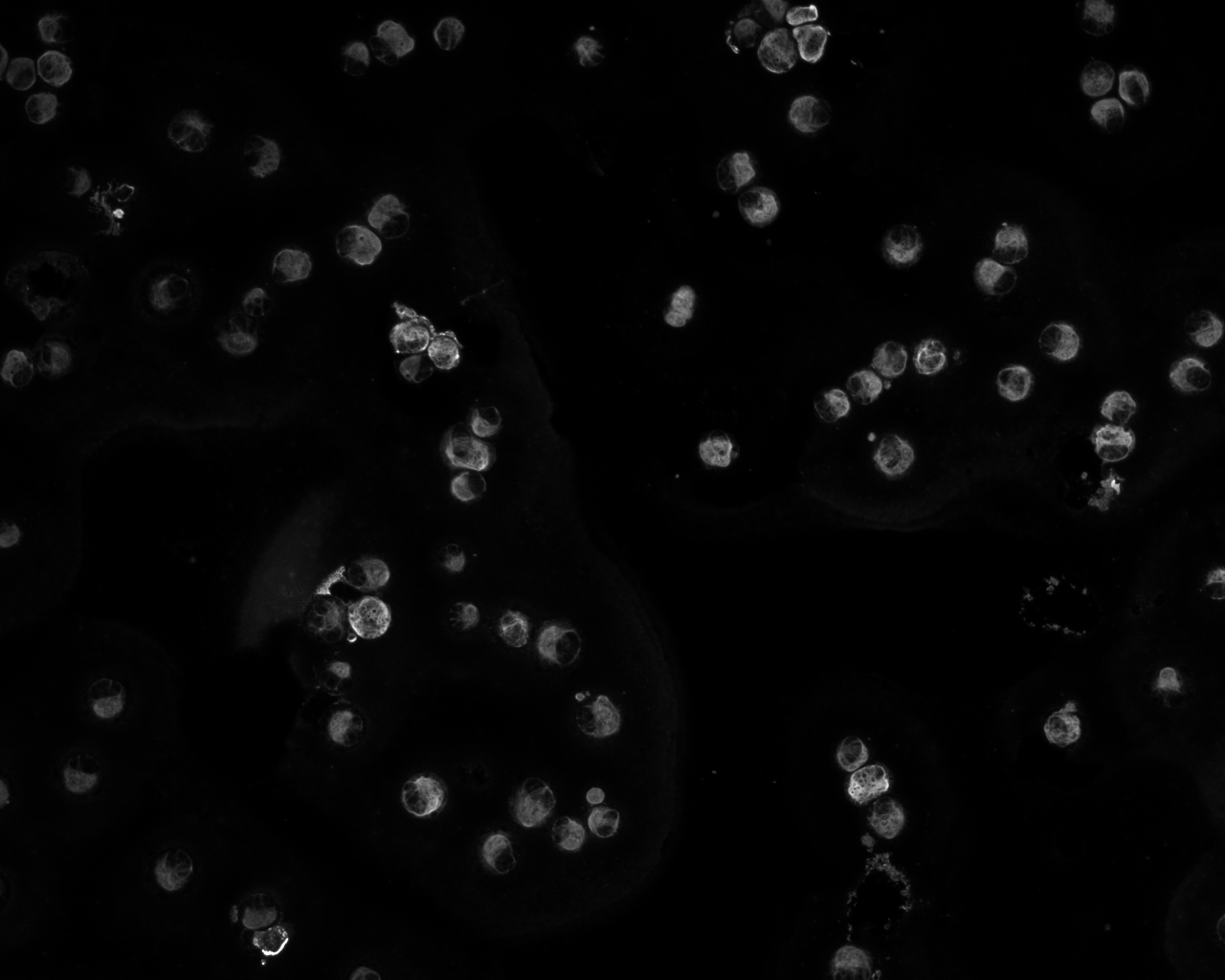

Supplement: S3 Data — (ZIP) [file pone.0233647.s006.zip › PACE Corrected/rSC51_SOX10AF488_EdUAF555_VIMEDL650_DAPI_20x_2_VIME 3.tif]
